# ^18^F-Difluoromethyl(ene) Motifs via Oxidative Fluorodecarboxylation with [^18^F]Fluoride

**DOI:** 10.1021/acs.orglett.4c03611

**Published:** 2024-10-23

**Authors:** Sebastiano Ortalli, Joseph Ford, Robert Szpera, Barbara Stoessel, Andrés A. Trabanco, Matthew Tredwell, Véronique Gouverneur

**Affiliations:** † Chemistry Research Laboratory, University of Oxford, 12 Mansfield Road, Oxford OX1 3TA, United Kingdom; ‡ Global Discovery Chemistry, Therapeutics Discovery, Johnson & Johnson Innovative Medicine, Janssen-Cilag, S.A., E-45007 Toledo, Spain; § Wales Research and Diagnostic PET Imaging Centre, Cardiff University, University Hospital of Wales, Heath Park, Cardiff CF14 4XN, United Kingdom; ∥ School of Chemistry, Cardiff University, Main Building, Park Place, Cardiff CF10 3AT, United Kingdom

## Abstract

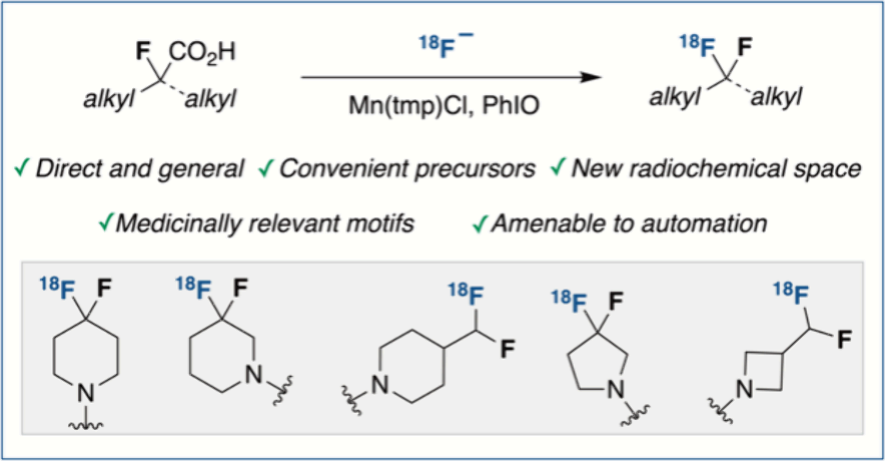

Herein, we report that α-fluorocarboxylic acids undergo manganese-mediated oxidative ^18^F-fluorodecarboxylation with [^18^F]fluoride affording biologically relevant ^18^F-difluoromethyl(ene)-containing molecules. This no-carrier added process provides a solution to a known challenge in radiochemistry and expands the radiochemical space available for positron emission tomography (PET) ligand discovery. Scalability on a fully automated radiosynthetic platform is exemplified with the production of [^18^F]4,4-difluoropiperidine that, we demonstrate, is amenable to postlabeling functionalization including *N*-heteroarylation and amide as well as sulfonamide bond formation.

The geminal difluoro motif is highly prevalent in pharmaceuticals, agrochemicals and functional materials (Figure 1).^1^ Its incorporation into bioactive compounds has been demonstrated to impart profound pharmacokinetic and physicochemical effects, including modulation of lipophilicity, metabolic stability, and the p*K*_a_ of adjacent functional groups.^2^ The unique properties of the *gem*-difluoro group also render it an ideal bioisostere of various functionalities, such as carbonyl, sulfonyl, and oxygen atoms.^3^ More specifically, the difluoromethyl (CF_2_H) group is able to exert conformational effects and engage in hydrogen-bonding interactions, providing opportunities for the enhancement of drug potency and selectivity.^4^ These unique characteristics have encouraged the development of numerous synthetic routes to *gem*-difluoroalkanes. Beyond the well-established deoxyfluorination of carbonyl groups, novel and orthogonal methodologies have been successfully pursued.^5^

**Figure 1:**
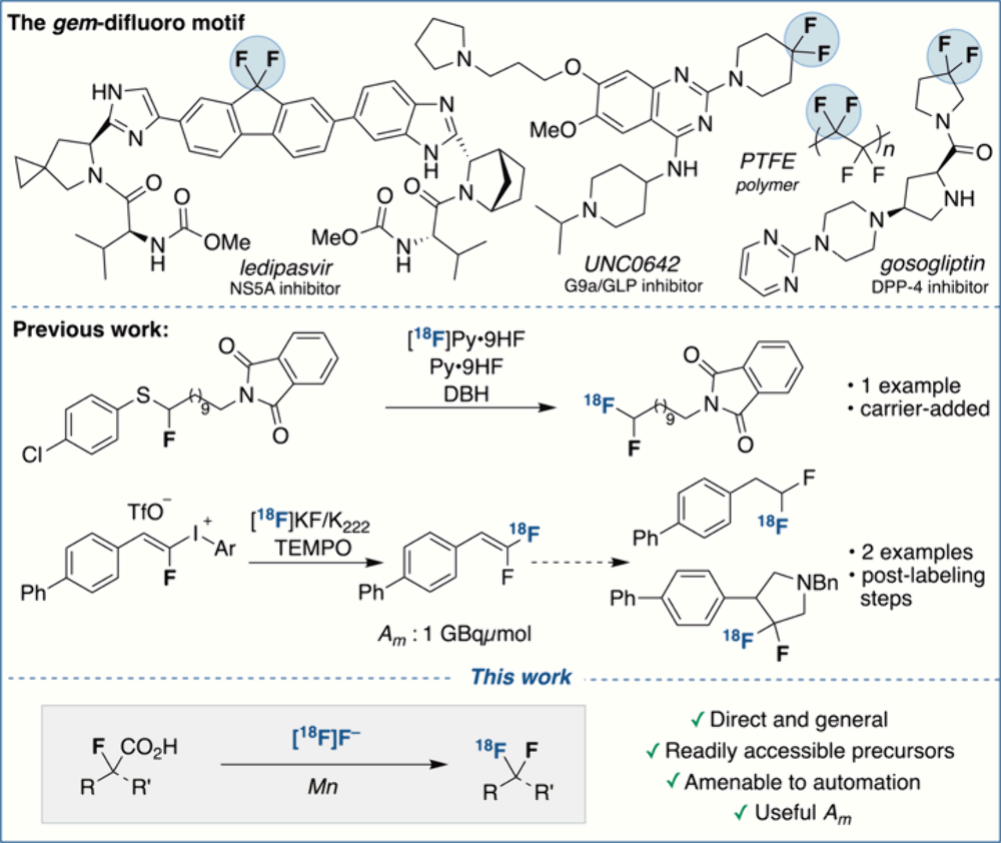
Prevalence of the *gem*-difluoro motif, previous methods for the synthesis of the ^18^F-difluoromethyl(ene) motif, and this work: synthesis of ^18^F-difluoromethyl(ene) motifs via fluorodecarboxylation with [^18^F]fluoride.

Positron emission tomography (PET) imaging is a highly sensitive, quantitative imaging technology that can greatly accelerate drug development.^6^ The technology hinges on the detection of γ rays generated by the decay of a positron (β^+^)-emitting radionuclide incorporated within a radiotracer and administered to patients. Fluorine-18 is ideally suited to this application due to its outstanding properties (e.g., 97% β^+^ decay, 0.635 MeV β^+^ energy) and is consequently routinely deployed in the clinic.^7^ Furthermore, its half-life of 109.8 min permits the multistep radiosynthesis of radiopharmaceuticals and their transportation to satellite imaging centers. While the synthesis of aryl and α-heteroatom ^18^F-difluoromethyl compounds has been well explored, access to molecules featuring the *gem*-^18^F-difluoromethyl(ene) motif at less activated positions remains a challenge in radiochemistry.^8^ At present, the synthesis of *gem*-^18^F-difluoroalkanes is indeed limited to two examples. In 2010, Haufe and co-workers disclosed a protocol for the synthesis of terminal *gem*-difluoroalkanes via a desulfurization-difluorination reaction of thioethers in the presence of pyridine polyhydrofluoride (Py·9HF) and 1,3-dibromo-5,5-dimethylhydantoin serving as an oxidant.^9^ Translation of this methodology to the radiofluorination of an α-fluorinated thioether with no-carrier-added [^18^F]KF proved unsuccessful, but the use of carrier-added [^18^F]Py·HF led to a single example of a *gem*-^18^F-difluoroalkane in 9% RCY (Figure 1). More recently, Tredwell and co-workers reported the synthesis of *gem*-^18^F-difluoroalkenes with [^18^F]KF, using fluoroalkenyl-(aryl)iodonium triflates, a class of substrates accessible from aldehydes in three steps.^10^ Molar activity (*A*_m_) reached 1 GBq/μmol. Further product derivatization reactions, including reduction and 1,3-dipolar cycloaddition, enabled the radiosynthesis of two *gem*-^18^F-difluoroalkanes (Figure 1).

Our aim was to develop a protocol, ideally amenable to automation, for the synthesis of geminal ^18^F-difluoro(cyclo)alkanes from easily accessible precursors and [^18^F]fluoride. We envisioned subjecting a monofluorinated substrate class to ^18^F-fluorination in order to avoid postlabeling ^19^F-fluorination or the requirement to prepare preformed ^18^F-difluoromethylene transfer reagents.^8,11^ This direct approach allows for a shorter radiosynthesis time, a key benefit in ^18^F-radiochemistry due to the loss of radioactivity due to the decay of fluorine-18 (*t*_1/2_ = 109.8 min). In terms of reaction design, traditional two-electron pathways featuring the displacement of leaving groups such as (pseudo)halides by [^18^F]fluoride pose significant challenges, due to the diminished reactivity of fluorinated carbon centers toward nucleophilic substitution reactions.^8,12^ Instead, we selected α-fluorocarboxylic acids guided by a previous report in our group describing the beneficial effect of α-fluoro substitution for a ^18^F-fluorodecarboxylative process leading to ^18^F-difluoromethyl arenes.^13^ α-Fluorocarboxylic acid precursors present the additional advantage of being commercially available or easily accessible from ubiquitous esters via electrophilic fluorination followed by hydrolysis, among other methods,^14^ minimizing synthetic bottlenecks.

Preliminary experiments focused on assessing the reactivity of 1-benzoyl-4-fluoropiperidine-4-carboxylic acid **1** toward ^18^F-fluorodecarboxylation with [^18^F]TEAF (Table 1). Pleasingly, in the presence of Mn(tmp)Cl (**3**) and the oxidant PhIO, the desired radiolabeled *gem*-difluorinated product [^18^F]**2** was obtained in 46% RCY upon heating the reaction mixture at 80 °C over 20 min in 1,2-DCE (Table 1, entry 1). Alternative solvents, such as DMF and CHCl_3_, were also compatible albeit slightly less suitable for this transformation (Table 1, entries 2, 3). Performing the reaction at lower temperatures, e.g., 50 °C instead of 80 °C, still enabled the formation of the desired radiofluorinated product [^18^F]**2** in lower RCY (Table 1, entry 4). Different loadings of the Mn species **3** and the oxidant PhIO led to [^18^F]**2** in similar or diminished RCY (Table 1, entries 5–8).

**Table 1:**
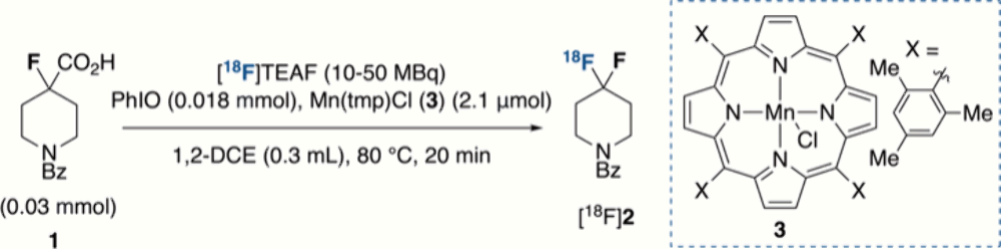
Optimization of the Reaction Conditions^a^

entry	deviation from standard conditions	RCY (%)
1	none	46 ± 14_*n*=4_
2	DMF as solvent	44_*n*=1_
3	CHCl_3_ as solvent	40 _*n*=1_
4	50 °C instead of 80 °C	27_*n*=1_
5	9 μmol PhIO	16_*n*=1_
6	36 μmol PhIO	49_*n*=1_
7	1 μmol **3**	32_*n*=1_
8	4 μmol **3**	26_*n*=1_

a [^18^F]TEAF was prepared with a NEt_4_HCO_3_ (9 mg) elution protocol. Bz: benzoyl. RCY: radiochemical yield, determined by radioHPLC analysis of the crude reaction mixture.

With the optimized conditions in hand, the scope of our ^18^F-fluorodecarboxylative protocol was investigated next (Scheme 1). In line with the outcome of a preliminary robustness screen (Figure s1),^15,16^ the reaction was found to be compatible with numerous functional groups, such as amide ([^18^F]**2**), carbamate ([^18^F]**4**, [^18^F]**6**, [^18^F]**8**, [^18^F]**9**), sulfonamide ([^18^F]**7**), aryl chloride ([^18^F]**10**), and phthalimide ([^18^F]**13**). The geminal ^18^F-difluoro motif was successfully installed within the ubiquitous piperidine scaffold,^17^ both at the 4- ([^18^F]**2**, ([^18^F]**4**) and 3-positions ([^18^F]**6**), as well as exocyclically ([^18^F]**7**). Additional *N*-heterocyclic geminal difluorides, such as Boc-protected pyrrolidine ([^18^F]**8**) and azetidine ([^18^F]**9**), were radiolabeled in moderate RCY. Furthermore, a cyclohexane derivative underwent ^18^F-fluorodecarboxylation in good RCY ([^18^F]**10**). Notably, these structures feature prominently in compounds of biological interest.^2a,2b,18^ Beyond cyclic substrates, fluorine-18 was successfully introduced within alkyl chains of varying lengths and substitution patterns ([^18^F]**11**–**13**). The adamantane moiety, often considered as a lead structure in the development of novel pharmaceuticals,^19^ was also compatible with our conditions, furnishing [^18^F]**14** in 38% RCY. The reaction was suitable for ^18^F-labeling at the benzylic position, enabling the radiolabeling of 9,9-difluoro-9*H*-fluorene ([^18^F]**15**), a motif contained in the core structure of the antiviral ledipasvir (Figure 1). Last, the ^18^F-trifluoromethyl group was also within reach of this transformation, as exemplified by the radiosynthesis of [^18^F]**16**. Unsuccessful substrates can be found in the Supporting Information (Figure S8).

**Scheme 1:**
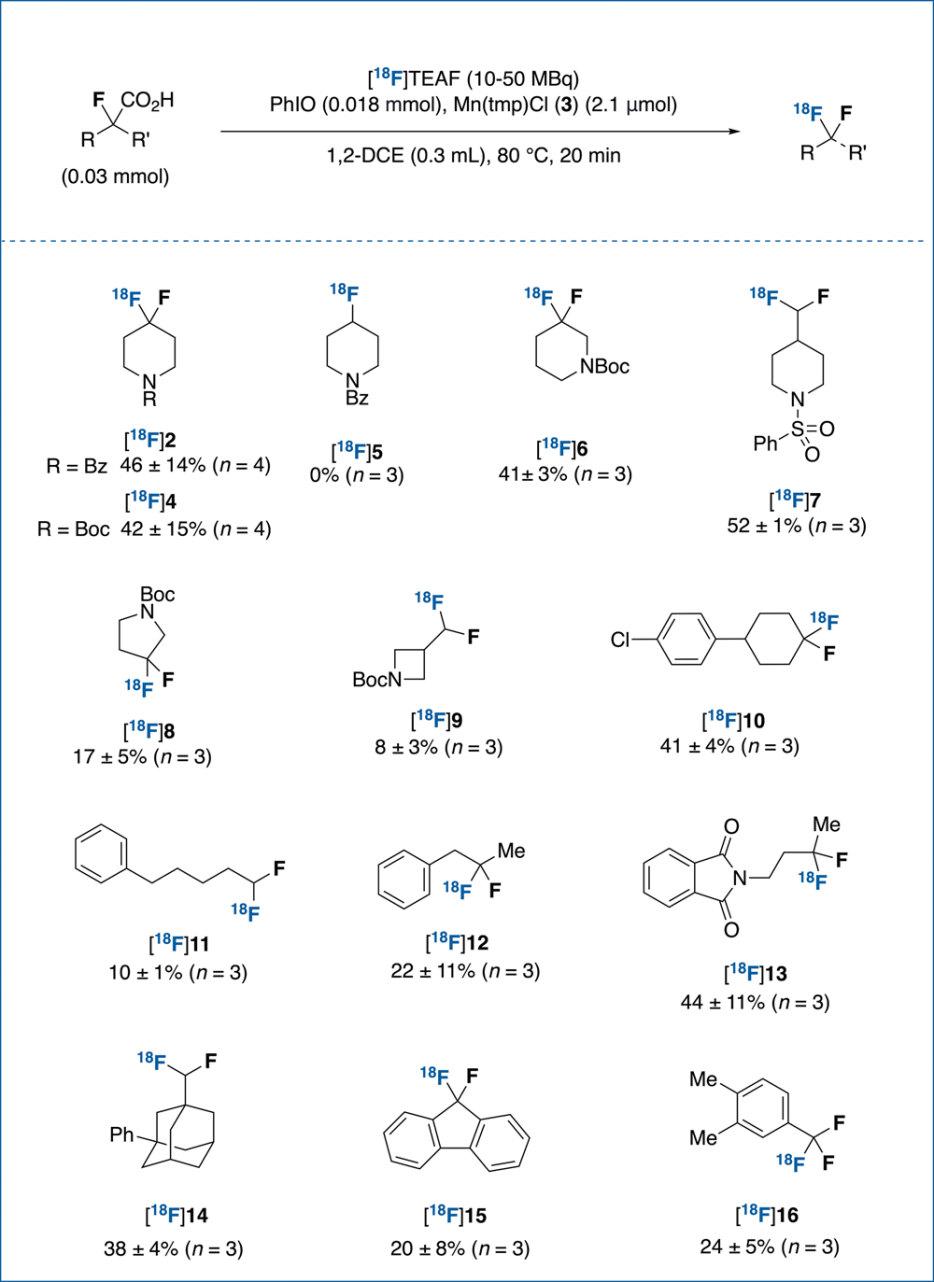
Scope of the ^18^F-Fluorodecarboxylation Reaction [^18^F]TEAF was prepared with a NEt_4_HCO_3_ (9 mg) elution protocol. Bz: benzoyl. RCY: radiochemical yield determined by radioHPLC analysis of the crude reaction mixture.

Similarly to a seminal report by Groves and co-workers on the Mn-mediated ^18^F-fluorodecarboxylation of carboxylic acids,^13b^ we suggest that the reaction proceeds via ^18^F-fluorine atom transfer between a ^18^F-fluoromanganese(IV) complex and an α-fluoroalkyl radical formed upon decarboxylation of the α-fluorocarboxylic acid substrate.

In line with previous mechanistic observations,^13a^ the presence of a fluorine atom at the α-carbonyl position was found to be crucial for reactivity, likely providing stabilization of the resulting carbon-centered radical via resonance effects.^20^ In the absence of α-fluoro substitution, an analogue of model substrate **1** led to no ^18^F-incorporation ([^18^F]**5**) under the standard reaction conditions (Scheme 1).

For (pre)clinical applications, automation of the reaction on a commercial platform is crucial as it permits the safe and reliable synthesis of radiotracers on multi-GBq scale. Hence, we set out to explore the feasibility of performing our ^18^F-fluorodecarboxylation protocol with a TRASIS AllinOne synthesizer. For the efficient transfer of the reaction mixture within the radiosynthetic module, soluble PhI(OAc)_2_ was selected in place of PhIO. In addition, manganese species **3** was directly employed for the elution of [^18^F]fluoride from the ion exchange cartridge to afford [^18^F]Mn(tmp)F with an elution efficiency of 94%.^13^

An automated program inclusive of semipreparative HPLC purification thus enabled the radiosynthesis of [^18^F]**4** in an AY of 1.74 GBq from 50 GBq starting activity and high chemical and radiochemical purity (>99%) with a total synthesis time of 78 min (Scheme 2). *A*_m_ reached 6.35 GBq/μmol (d.c. EOS). An added advantage, in contrast to previous reports of ^18^F-fluorodecarboxylative strategies, is the direct use of an α-fluorocarboxylic acid substrate, bypassing the time-consuming requirement for the isolation of an activated preformed iodine(III) complex.^13^ The residual amount of manganese in the purified sample of [^18^F]**4** was measured by ICP-MS and was found to be 4.1 μg/L, thereby meeting the ICH guidelines for pharmaceuticals destined for human use.^15^

**Scheme 2:**
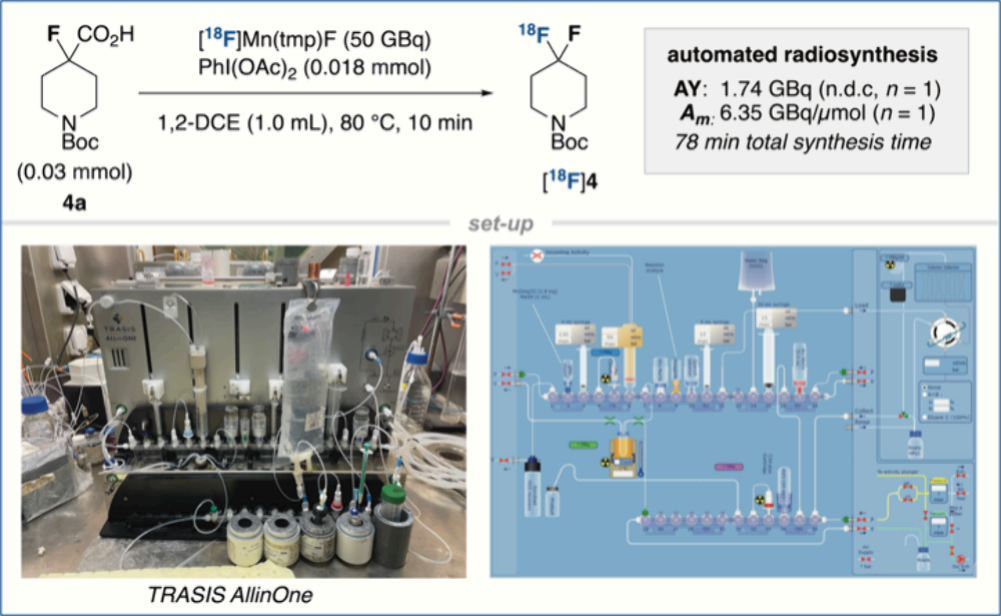
Automation of the the ^18^F-Fluorodecarboxylation Reaction AY (n.d.c.): activity yield (non decay-corrected); *A*_*m*_ (d.c. EOS): molar activity (decay-corrected to the end-of-synthesis). Automated radiosynthesis was achieved using a Trasis AllinOne platform.

With an automated radiosynthetic protocol secured for [^18^F]**4**, we set out to demonstrate its value as a versatile building block in radiochemistry, encouraged by the privileged role of piperidine in medicinal chemistry campaigns, as highlighted by a recent study identifying it as the most common *N*-heterocycle in small-molecule pharmaceuticals.^17a^ [^18^F]4,4-Difluoropiperidine ([^18^F]**18**) was expediently accessed upon Boc-deprotection of [^18^F]**4** and engaged in a series of diversification reactions (Scheme 3).^15^ For example, treatment of [^18^F]**18** with 6-chloro-9-ethyl-9*H*-purine afforded the S_N_Ar product [^18^F]**19** in excellent RCY. An amide coupling protocol with a *N*-hydroxysuccinimide ester was also successful, providing a radiofluorinated derivative of the antiepileptic agent ilepcimide ([^18^F]**20**) in 65% RCY. Last, sulfonamide [^18^F]**21**, displaying PDE5 inhibitory activity, was prepared in 92% RCY via condensation of [^18^F]**18** with the requisite sulfonyl chloride precursor.^21^

**Scheme 3:**
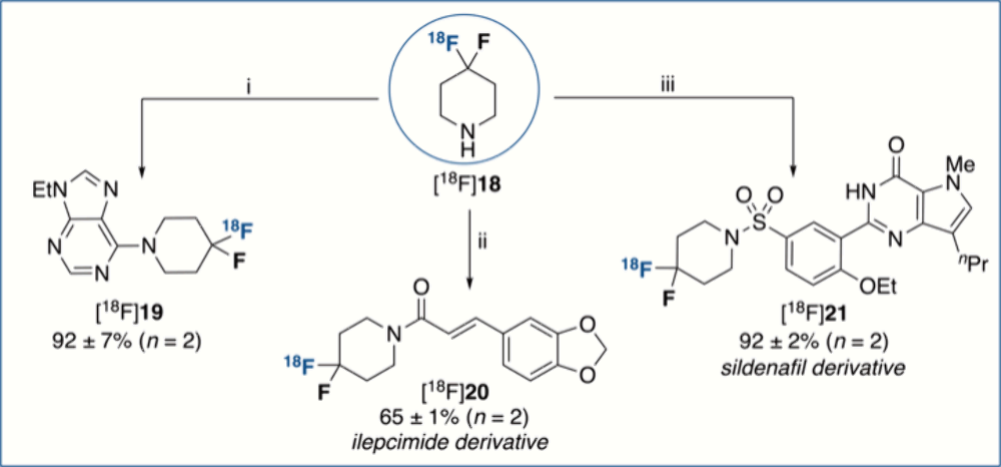
Derivatization Reactions of [^18^F]4,4-Difluoropiperidine RCY: radiochemical yield determined by radioHPLC analysis of the crude reaction mixture. (i) 6-Chloro-9-ethyl-9*H*-purine (0.05 mmol), K_2_CO_3_ (0.05 mmol), DMSO (0.5 mL), 110 °C, 20 min; (ii) 2,5-dioxopyrrolidin-1-yl (*E*)-3-(benzo[*d*][1,3]dioxol-5-yl)acrylate (0.05 mmol), K_2_CO_3_ (0.05 mmol), DMA (0.5 mL), 40 °C, 20 min; (iii) 4-ethoxy-3-(1-methyl-7-oxo-3-propyl-6,7-dihydro-1*H*-pyrazolo[4,3-*d*]pyrimidin-5-yl)benzene-1-sulfonyl chloride (0.05 mmol), NEt_3_ (0.1 mmol), and THF (0.5 mL), 40 °C, 20 min.

In conclusion, we have developed a protocol for the synthesis of geminal ^18^F-difluoroalkanes via manganese-mediated ^18^F-fluorodecarboxylation of easily accessible α-fluorocarboxylic acids with [^18^F]fluoride. This first direct radiosynthesis of the geminal ^18^F-difluoromethyl(ene) motif under no-carrier added conditions was applied to various scaffolds, including medicinally relevant cyclic amines. Scalability and translation to a fully automated radiosynthesis platform were also demonstrated, furnishing radiolabeled products in useful AY and *A*_m_. The value of [^18^F]4,4-difluoropiperidine as a versatile building block is further demonstrated with the assembly of complex and biorelevant radiolabeled scaffolds. Given the significance of the geminal difluoro motif in functional materials as well as medicinal and agrochemistry, we anticipate that the novel radiochemical space accessible with this technology will spark meaningful innovation in the development of radiotracers for applications in PET imaging.

## Data Availability

The data underlying this study are available in the published article and its Supporting Information.

## Supporting Information Available

Preparation of starting materials, labeling precursors and reference materials; radiofluorination methods; (radio)HPLC traces; NMR spectra (PDF)
