# Supplementary material for: 18F-Difluoromethyl(ene) Motifs via Oxidative Fluorodecarboxylation with [18F]Fluoride
Source: Org Lett. 2024 Oct 23;26(43):9368–72. doi: 10.1021/acs.orglett.4c03611 (PMC11536415; doi:10.1021/acs.orglett.4c03611)
Supplement: Supplementary file 1 — ol4c03611_si_001.pdf [file ol4c03611_si_001.pdf]

Supporting Information for

^{18}F-Difluoromethyl(ene) Motifs *via* Oxidative Fluorodecarboxylation with [^{18}F]Fluoride

Sebastiano Ortalli[a], Joseph Ford[a], Robert Szpera[a], Barbara Stoessel[a], Andrés A. Trabanco[b], Matthew Tredwell[c,d] and Véronique Gouverneur[a,*]

[a] *Department of Chemistry, Chemistry Research Laboratory, University of Oxford, Mansfield Road, Oxford, OX1 3TA, U.K.*

[b] *Discovery Chemistry, Janssen Research and Development, Janssen-Cilag, S.A., E-45007, Toledo, Spain.*

[c] *Wales Research and Diagnostic PET Imaging Centre, Cardiff University, University Hospital of Wales, Heath Park, Cardiff, CF14 4XN, U.K.*

[d] *School of Chemistry, Cardiff University, Main Building, Park Place, Cardiff, CF10 3AT, U.K.*

[*]*Correspondence should be addressed to veronique.gouverneur@chem.ox.ac.uk*

Table of contents

General information……………………………………………………………s3

Non-radioactive experiments……………………………………………………s4

Synthesis of starting materials and reference compounds ……………………s4

Radiochemistry……………………………………………………………………s15

General experimental details…………………………………………………s15

Optimisation of reaction conditions……………………………………………s17

Robustness screen………………………………………………………………s19

Radiotrace overlays and radiochemical yields…………………………………s21

Automation ………………………………………………………………………s35

ICP-MS……………………………………………………………………………s43

Synthesis of [^{18}F]4,4-difluoropiperidine …………………………………s44

Post-labeling reactions…………………………………………………………s46

Unsuccessful substrates…………………………………………………………s49

References ………………………………………………………………………s50

NMR spectra ……………………………………………………………………s51

General information

Dry solvents were purchased from commercial suppliers or dried on a column of alumina. Reactions were monitored by thin-layer chromatography (TLC) on silica gel pre-coated aluminium sheets (Merck Kieselgel 60 F254 plates). Visualization was accomplished by irradiation with UV light at 254 nm, and/or potassium permanganate stain. Column chromatography was performed on Merck silica gel (60, particle size 0.040-0.063 mm). Preparative thin layer chromatography was performed on Analtech silica gel GF 20x20 cm 500-1500 micron plates. Heating of reactions was accomplished using oil baths. Heating of radiochemical reactions was accomplished using aluminium heating blocks. All NMR spectra were recorded on Bruker AVIIIHD 400, Bruker NEO 400, AVIIIHD 500, Bruker AVIII 600, or Bruker NEO 600. ^{1}H and ^{13}C NMR spectral data are reported as chemical shifts (δ) in parts per million (ppm) relative to the solvent peak using the Bruker internal referencing procedure (edlock). ^{19}F NMR spectra are referenced relative to $CFCl_3$. Coupling constants, J, are reported in Hz to the nearest 0.1 Hz. Unless otherwise stated, ^{13}C spectra are ^{1}H decoupled and reported coupling constants for ^{13}C spectra correspond to ^{19}F–^{13}C heteronuclear coupling. Data are reported as follows: chemical shift, multiplicity (s = singlet, d = doublet, t = triplet, q = quartet, pent = pentet, hept = heptet, br = broad, m = multiplet), coupling constants (Hz) and integration. NMR spectra were processed with MestReNova 14.2.0. Structural assignments were made with additional information from gCOSY, gHSQC, and gHMBC experiments. High resolution mass spectra were determined on a Thermo Exactive mass spectrometer, for electrospray ionization (ESI-TOF), or an Agilent 7200 Accurate Mass Q-TOF GC-MS connected to a 7890 GC system, for electron ionization (GC-EI). Infrared spectra were recorded as the neat compound or as an evaporated solution using a Bruker Tensor 27 FTIR spectrometer. Absorptions are reported in wavenumber (cm^{-1}). Melting points of solids were measured on a Stuart SMP20 melting point apparatus and are uncorrected. IUPAC names were obtained using ChemDraw 19.1.1.32. Weighing was performed with a 4 or 5 decimal place balance. All commercially available chemicals were purchased from commercial suppliers or otherwise synthesized according to literature. For manual radiochemistry experiments: [^{18}F]Fluoride was produced by Invicro (UK) or PETIC (UK) *via* the ^{18}O(p,n)^{18}F reaction and delivered as [^{18}F]fluoride in [^{18}O]water. Radiosynthesis and azeotropic drying were performed on a NanoTek® automated microfluidic device (Advion) or on an AllinOne radiosynthesizer (TRASIS). For automated radiochemistry experiments: [^{18}F]Fluoride was produced in an IBA Cyclon 18/9 cyclotron using the ^{18}O(p,n)^{18}F reaction in PETIC (UK). All experiments were performed on an AllinOne radiosynthesizer (TRASIS). All isolated activity yields are non-decay-corrected (n.d.c.). All molar activities are decay-corrected to the end of synthesis (EOS), unless stated otherwise.

Non-radioactive experiments

Synthesis of starting materials and reference compounds

(1*R*,2*R*)-1-fluoro-2-phenylcyclopropane-1-carboxylic acid, (2*E*)-3-(1,3-benzodioxol-5-yl)-2-propenoic acid, (3-phenyl-1-adamantyl)acetic acid, (diacetoxyiodo)benzene (PIDA), 1,2-dimethyl-4-(trifluoromethyl)benzene (**16**), 1-(*tert*-butoxycarbonyl)-3-fluoropiperidine-3-carboxylic acid, 1-(*tert*-butoxycarbonyl)-3-fluoropyrrolidine-3-carboxylic acid, 1-(*tert*-butoxycarbonyl)-4-fluoropiperidine-4-carboxylic acid (**4a**), 1-[(*tert*-butoxy)carbonyl]-3-fluoroazetidine-3-carboxylic acid, 1-benzoylpiperidine-4-carboxylic acid, (1-fluoro-4-oxo-cyclohexanecarboxylic acid, 2-(3,4-dimethylphenyl)-2,2-difluoroacetic acid, 2-[(*tert*-butoxy)carbonyl]-6-fluoro-2-azaspiro[3.3]heptane-6-carboxylic acid, 2-fluoro-2-(1*H*-imidazol-1-yl)acetic acid, 3-(difluoromethyl)azetidine hydrochloride, 4,4-difluoropiperidine hydrochloride, 4,4-difluoropiperidine, 4-(difluoromethyl)piperidine hydrochloride, 4-ethoxy-3-(1-methyl-7-oxo-3-propyl-6,7-dihydro-1*H*-pyrazolo[4,3-*d*]pyrimidin-5-yl)benzene-1-sulfonyl chloride, 5,10,15,20-tetrakis-(2,4,6-trimethylphenyl)-porphine-Mn(III) chloride (Mn(tmp)Cl) (**3**), 6-chloro-9-ethyl-9*H*-purine, butyl 1-fluorocyclohex-3-enecarboxylate, iodosylbenzene, methyl 2-(piperidin-4-yl)acetate hydrochloride, *tert*-butyl 3,3-difluoropiperidine-1-carboxylate (**6**), *tert*-butyl 3,3-difluoropyrrolidine-1-carboxylate (**8**), and *tert*-butyl 4,4-difluoropiperidine-1-carboxylate (**4**) were purchased from commercial suppliers (Fluorochem, Sigma Aldrich, TCI, BLDpharm, Apollo Scientific, Ambeed, Manchester Organics, Biosynth) and used as received, without further purification.

(2,2-difluoropropyl)benzene (**12**),[1] (4,4-difluoropiperidin-1-yl)(phenyl)methanone (**2**),[2] (5,5-difluoropentyl)benzene (**11**),[3] 1-benzoyl-4-fluoropiperidine-4-carboxylic acid (**1**),[1] 1-chloro-4-(4,4-difluorocyclohexyl)benzene (**10**),[1] 2-(3,3-difluorobutyl)isoindoline-1,3-dione (**13**),[1] 2-(5-((4,4-difluoropiperidin-1-yl)sulfonyl)-2-ethoxyphenyl)-5-methyl-7-propyl-3,4a,5,7a-tetrahydro-4*H*-pyrrolo[3,2-*d*]pyrimidin-4-one (**21**),[4] 2-fluoro-2-methyl-3-phenylpropanoic acid ,[1] 4-(1,3-dioxoisoindolin-2-yl)-2-fluoro-2-methylbutanoic acid,[1] 9-fluoro-9*H*-fluorene-9-carboxylic acid,[5] and 9,9-difluoro-9*H*-fluorene (**15**)[5] were synthesized according to literature procedures. 4-(4-chlorophenyl)-1-fluorocyclohexane-1-carboxylic acid was synthesized according to literature procedure and the major diastereomer was used as radiolabelling precursor.[1] All spectroscopic data were in accordance with the literature.

2-Fluoro-2-(1-(phenylsulfonyl)piperidin-4-yl)acetic acid (**7a**)

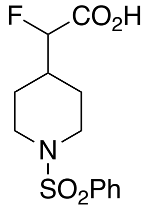

To a round bottom flask equipped with a magnetic stirrer bar was added methyl 2-fluoro-2-(1-(phenylsulfonyl)piperidin-4-yl)acetate (**7b**) (520 mg, 1.65 mmol, 1.0 equiv), followed by THF (4.2 mL) and a solution of $LiOH \cdot H_2O$ (208 mg, 4.95 mmol, 3.0 equiv) in H_2O (4.2 mL). The reaction mixture was stirred at room temperature for 16 h, then acidified to pH ≈ 1 by the addition of an aqueous 6 M HCl solution and extracted with EtOAc three times. The combined organic layers were dried over Na_2SO_4 and evaporated *in vacuo*. The crude product was purified by silica gel column chromatography (CH_2Cl_2/EtOAc/formic acid, 99:0:1 to 5:94:1) to give the product as a white solid (222 mg, 0.74 mmol, 45%). **^{1}H NMR** (400 MHz, DMSO-*d*$_6$) δ 7.77–7.68 (m, 3H), 7.67–7.61 (m, 2H), 4.84 (dd, *J* = 48.8, 3.9 Hz, 1H), 3.75–3.66 (m, 2H), 2.23–2.19 (m, 2H), 1.92–1.68 (m, 2H), 1.62–1.53 (m, 1H), 1.38 (qd, *J* = 12.6, 4.4 Hz, 2H); **^{19}F NMR** (377 MHz, DMSO-*d*$_6$) δ -198.07 (dd, *J* = 48.9, 24.6 Hz); **^{13}C NMR** (101 MHz, DMSO-*d*$_6$) δ 170.0 (d, *J* = 24.0 Hz), 135.8, 133.1, 129.4, 127.3, 90.6 (d, *J* = 182.7 Hz), 45.5 (d, *J* = 7.6 Hz), 36.9, 26.6 (d, *J* = 3.3 Hz), 24.8 (d, *J* = 4.7 Hz) (*note*: extra signal observed due to the presence of piperidine rotamers); **HRMS** (ESI-TOF) m/z: $[M - H]^-$ Calcd for $C_{13}H_{15}O_4NFS$ 300.0711; Found 300.0708; **m.p.**: 143–145 °C; **IR** (neat): 3206, 2954, 2926, 2849, 2359, 2342, 1743, 1473, 1448, 1402, 1329, 1312, 1248, 1219, 1157, 1129, 1112, 1090, 1054, 996, 964, 929, 887, 821, 759, 737, 685, 660.

Methyl 2-fluoro-2-(1-(phenylsulfonyl)piperidin-4-yl)acetate (**7b**)

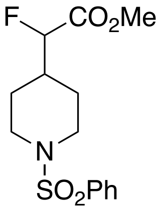

To a flame-dried round bottom flask equipped with a magnetic stirrer bar was added anhydrous THF (4.0 mL) followed by anhydrous $^{i}Pr_2NH$ (882 μL, 6.29 mmol, 1.3 equiv) under N_2. The flask was cooled to -78 °C prior to the dropwise addition of $^{n}BuLi$ (2.5 M solution in THF, 2.32 mL, 5.81 mmol, 1.2 equiv). The reaction mixture was stirred at -78 °C for 20 min, then at 0 °C for 20 min. A solution of methyl 2-(1-(phenylsulfonyl)piperidin-4-yl)acetate (**7c**) (1.439 g, 4.84 mmol, 1.0 equiv) in anhydrous THF (16.0 mL) was then added dropwise at -78 °C (*note*: the solution was gently heated under N_2 to ensure complete solubilization) and the reaction mixture was stirred at -78 °C for 20 min, then at 0 °C for 30 min. A solution of NFSI (1.984 g, 6.29 mmol, 1.3 equiv) in anhydrous THF (10.0 mL) was then added dropwise at -78 °C and the reaction mixture stirred at -78 °C for 30 min, then at room temperature for 16 h. Subsequently, the reaction mixture was diluted with H_2O and extracted with CH_2Cl_2 three times. The combined organic layers were washed with a saturated aqueous NaCl solution, dried over Na_2SO_4 and evaporated *in vacuo*. The crude product was purified by silica gel column chromatography (pentane/EtOAc, 100:0 to 70:30) to give the product as a clear oil (520 mg, 1.65 mmol, 34%). **^{1}H NMR** (400 MHz, $CDCl_3$) δ 7.78–7.74 (m, 2H), 7.63–7.58 (m, 1H), 7.57–7.50 (m, 2H), 4.71 (dd, J = 48.5, 4.5 Hz, 1H), 3.92–3.84 (m, 2H), 3.77 (s, 3H), 2.35–2.19 (m, 2H), 1.92–1.56 (m, 5H); **^{19}F NMR** (377 MHz, $CDCl_3$) δ -198.31 (dd, J = 48.6, 21.7 Hz); **^{13}C NMR** (101 MHz, $CDCl_3$) δ 169.2 (d, J = 24.0 Hz), 136.3, 133.0, 129.2, 127.8, 91.3 (d, J = 188.2 Hz), 52.5, 45.9 (d, J = 5.1 Hz), 38.2 (d, J = 20.3 Hz), 27.0 (d, J = 3.3 Hz), 25.6 (d, J = 4.7 Hz) (*note*: extra signal observed due to the presence of piperidine rotamers); **HRMS** (ESI-TOF) m/z: $[M + H]^+$ Calcd for $C_{14}H_{19}O_4NFS$ 316.1013; Found 316.1016; **IR** (thin layer film): 2956, 2923, 2854, 2360, 2341, 1762, 1699, 1653, 1559, 1541, 1507, 1448, 1341, 1287, 1251, 1221, 1171, 1132, 1095, 1056, 1021, 936, 741, 693, 668, 648; **m.p.**: 80–82 °C.

Methyl 2-(1-(phenylsulfonyl)piperidin-4-yl)acetate (**7c**)

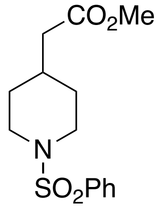

To a flame-dried round bottom flask equipped with a magnetic stirrer bar was added methyl 2-(piperidin-4-yl)acetate hydrochloride (999 mg, 5.16 mmol, 1.0 equiv). The flask was evacuated

and backfilled with N_2 three times prior to the addition of anhydrous CH_2Cl_2 (16 mL, 0.32 M) and anhydrous $^{i}Pr_2NEt$ (2.25 mL, 12.9 mmol, 2.5 equiv). Benzene sulfonyl chloride (0.79 mL, 6.19 mmol, 1.2 equiv) was then added dropwise at 0 °C. The reaction mixture was stirred at room temperature under N_2 for 16 h, then washed with H_2O. The organic layer was dried over Na_2SO_4 and evaporated *in vacuo*. The crude product was purified by silica gel column chromatography (pentane/CH_2Cl_2, 100:0 to 70:30) to give the product as a clear oil (1.438 g, 4.84 mmol, 94%). **^{1}H NMR** (400 MHz, $CDCl_3$) δ 7.78–7.73 (m, 2H), 7.83–7.57 (m, 1H), 7.56–7.50 (m, 2H), 3.82–3.74 (m, 2H), 3.64 (s, 3H), 2.28 (td, J = 12.0, 2.6 Hz, 2H), 2.22 (d, J = 6.9 Hz, 2H), 1.82–1.60 (m, 3H), 1.42–1.28 (m, 2H); **^{13}C NMR** (101 MHz, $CDCl_3$) δ 172.7, 136.3, 132.8, 129.1, 127.8, 51.7, 46.3, 40.4, 32.2, 31.3; **HRMS** (ESI-TOF) m/z: $[M + H]^+$ Calcd for $C_{14}H_{20}O_4NS$ 298.1108; Found: 298.1104; **IR** (neat): 2959, 2842, 2360, 2342, 1733, 1450, 1415, 1368, 1350, 1332, 1276, 1252, 1219, 1181, 1165, 1147, 1126, 1094, 1049, 1015, 978, 957, 939, 906, 885, 824, 765, 739, 694, 611; **m.p.**: 135–138 °C.

2-Fluoro-6-phenylhexanoic acid (11a)

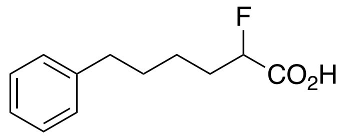

Synthesized according to literature procedure[6] from diethyl benzylmalonate and 1-bromo-4-phenylbutane to give the product as a white solid (828 mg, 3.94 mmol, 9% over 4 steps). **^{1}H NMR** (400 MHz, $CDCl_3$) δ 10.69 (brs, 1H), 7.32–7.26 (m, 2H), 7.23–7.16 (m, 3H), 5.03 (dd, J = 7.1, 4.8 Hz, 0.5H), 4.91 (dd, J = 6.8, 5.0 Hz, 0.5 H), 2.65 (t, J = 7.6 Hz, 2H), 2.07–1.90 (m, 2H), 1.77–1.64 (m, 2H), 1.62–1.51 (m, 2H); **^{19}F NMR** (377 MHz, $CDCl_3$) δ -192.41 (dt, J = 49.9, 25.7 Hz); **^{13}C NMR** (101 MHz, $CDCl_3$) δ 176.0 (d, J = 24.7 Hz), 142.1, 128.5, 126.0, 88.4 (d, J = 185.3 Hz), 35.7, 32.2 (d, J = 20.3 Hz), 31.0, 24.2 (d, J = 2.9 Hz); **HRMS** (ESI-TOF) m/z: $[M - H]^-$ Calcd for $C_{12}H_{14}FO_2$ 210.1062; Found 210.1010; **IR** (neat): 3029, 2939, 2862, 2574, 1726, 1712, 1604, 1497, 1441, 1303, 1256, 1154, 1098, 1054, 1018, 973, 926, 874, 749, 698, 676; **m.p.**: 37–39 °C.

2-Fluoro-2-((1*s*,3*r*,5*R*,7*S*)-3-phenyladamantan-1-yl)acetic acid (14a)

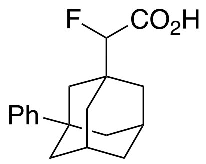

To a 7 mL glass vial equipped with a magnetic stirrer bar was added methyl 2-fluoro-2-((1*s*,3*r*,5*R*,7*S*)-3-phenyladamantan-1-yl)acetate (**14b**) (100 mg, 0.33 mmol, 1.0 equiv), followed by THF (0.8 mL) and a solution of LiOH·H_2O (69 mg, 1.65 mmol, 5.0 equiv) in H_2O (0.8 mL). The reaction mixture was stirred at room temperature for 16 h, then diluted with H_2O and washed with Et_2O twice. The aqueous layer was acidified to pH ≈ 1 by the addition of an aqueous 6 M HCl solution and extracted with CH_2Cl_2 three times. The combined organic layers were dried over Na_2SO_4 and evaporated *in vacuo* to give the product in quantitative yield as a white solid, which was not subjected to further purification. **^{1}H NMR** (400 MHz, DMSO-d_6) δ 13.22 (br s, 1H), 7.37–7.28 (m, 4H), 7.21–7.15 (m, 1H), 4.52 (d, J = 49.1 Hz, 1H), 2.20–2.13 (m, 2H), 1.88–1.57 (m, 12H); **^{19}F NMR** (377 MHz, DMSO-d_6) δ -197.81 (d, J = 49.9 Hz); **^{13}C NMR** (101 MHz, DMSO-d_6) δ 169.3 (d, J = 25.1 Hz), 150.0, 128.2, 125.7, 124.6, 95.0 (d, J = 184.6 Hz), 42.7 (d, J = 3.6 Hz), 41.7, 36.7 (d, J = 19.3 Hz), 36.1 (d, J = 8.7 Hz), 36.1 (d, J = 9.8 Hz), 35.3, 28.0; **HRMS** (ESI-TOF) m/z: $[M - H]^-$ Calcd for $C_{18}H_{20}FO_2$ 287.1453; Found 287.1451; **IR** (neat): 2919, 2851, 1713, 1494, 1453, 1363, 1343, 1299, 1117, 1061, 1043, 1001, 975, 938, 813, 759, 732, 698, 647; **m.p.**: 143–145 °C.

Methyl 2-fluoro-2-((1*s*,3*r*,5*R*,7*S*)-3-phenyladamantan-1-yl)acetate (**14b**)

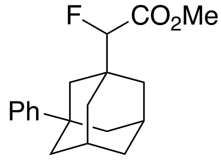

To a flame-dried round bottom flask equipped with a magnetic stirrer bar was added anhydrous THF (1.0 mL) followed by anhydrous ^{i}Pr_2NH (46 μL, 0.33 mmol, 1.3 equiv) under N_2. The flask was cooled to -78 °C prior to the dropwise addition of ^{n}BuLi (2.5 M solution in THF, 0.12 mL, 0.30 mmol, 1.2 equiv). The reaction mixture was stirred at -78 °C for 20 min, then at 0 °C for 20 min. A solution of methyl 2-((1*r*,3*s*,5*R*,7*S*)-3-phenyladamantan-1-yl)acetate (**14c**) (71 mg, 0.25 mmol, 1.0 equiv) in anhydrous THF (1.0 mL) was then added dropwise at -78 °C and the reaction mixture was stirred at -78 °C for 2 h. A solution of NFSI (120 mg, 0.38 mmol,

1.5 equiv) in anhydrous THF (1.0 mL) was then added dropwise at -78 °C and the reaction mixture stirred at -78 °C for 10 min, then at room temperature for 3 h. Subsequently, the reaction mixture was diluted with H_2O and extracted with EtOAc three times. The combined organic layers were washed with a saturated aqueous NaCl solution, dried over Na_2SO_4 and evaporated *in vacuo*. The crude product was purified by silica gel column chromatography (pentane/EtOAc, 100:0 to 95:5) to give the product as a clear oil (63 mg, 0.21 mmol, 83%). **^{1}H NMR** (400 MHz, $CDCl_3$) δ 7.39–7.29 (m, 4H), 7.23–7.17 (m, 1H), 4.49 (d, J = 48.7, 1H), 3.80 (s, 3H), 2.28–2.20 (m, 2H), 1.96–1.64 (m, 12H); **^{19}F NMR** (377 MHz, $CDCl_3$) δ -200.01 (d, J = 49.0 Hz); **^{13}C NMR** (101 MHz, $CDCl_3$) δ 168.9 (d, J = 25.4 Hz), 150.1, 128.4, 126.0, 125.0, 96.1 (d, J = 188.4 Hz), 52.0, 43.0 (d, J = 3.6 Hz), 42.4 (d, J = 7.5 Hz), 37.8 (d, J = 19.5 Hz), 36.9 (d, J = 4.0 Hz), 36.7 (d, J = 4.0 Hz), 35.9, 28.7 (d, J = 3.2 Hz); **HRMS** (ESI-TOF) m/z: $[M + Na]^+$ Calcd for $C_{19}H_{23}FO_2Na$ 325.1574; Found 325.1574; **IR** (neat): 2907, 2852, 2361, 2342, 1759, 1741, 1684, 1670, 1323, 1218, 1097, 1019, 912, 758, 733, 699.

Methyl 2-((1*r*,3*s*,5*R*,7*S*)-3-phenyladamantan-1-yl)acetate (**14c**)

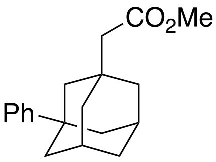

To a flame-dried Schlenk flask equipped with a magnetic stirrer bar was added (3-phenyl-1-adamantyl)acetic acid (100 mg, 0.37 mmol, 1.0 equiv) and the flask was evacuated and backfilled with N_2 three times prior to the addition of anhydrous CH_2Cl_2/DMF (9:1 v/v, 1 mL, 0.37 M). The reaction mixture was then cooled to 0 °C and oxalyl chloride (47 µL, 0.56 mmol, 1.5 equiv) was added dropwise under N_2. The reaction mixture was stirred at 0 °C for 1 h, then at room temperature for 2 h. Anhydrous pyridine (63 µL, 0.78 mmol, 2.1 equiv) was then added at 0 °C, followed by anhydrous MeOH (120 µL, 2.96 mmol, 8.0 equiv). The reaction mixture was stirred for 30 min at room temperature, then concentrated *in vacuo*. The residue was redissolved in Et_2O and washed with an aqueous saturated NaCl solution. The organic layer was dried over Na_2SO_4 and evaporated *in vacuo*. The crude product was purified by silica gel column chromatography (pentane/EtOAc, 100:0 to 95:5) to give the product as a clear oil (85 mg, 0.30 mmol, 81%). **^{1}H NMR** (400 MHz, $CDCl_3$) δ 7.40–7.29 (m, 4H), 7.23–7.16 (m, 1H), 3.66 (s, 3H), 2.27–2.14 (m, 4H), 1.94–1.81 (m, 4H), 1.77 (brs, 2H), 1.74–1.60 (m, 6H); **^{13}C**

NMR (101 MHz, $CDCl_3$) δ 172.2, 150.5, 128.3, 125.8, 125.0, 51.3, 48.6, 48.1, 42.4, 41.6, 37.1, 36.0, 33.8, 29.3; **HRMS** (ESI-TOF) m/z: $[M + H]^+$ Calcd for $C_{19}H_{25}O_2$ 285.1849; Found 285.1849; **IR** (neat): 3057, 3028, 2902, 2849, 2349, 1735, 1601, 1495, 1446, 1327, 1262, 1232, 1162, 1137, 1079, 1053, 1020, 904, 756, 699, 659, 628.

2,5-dioxopyrrolidin-1-yl (*E*)-3-(benzo[*d*][1,3]dioxol-5-yl)acrylate (20a)

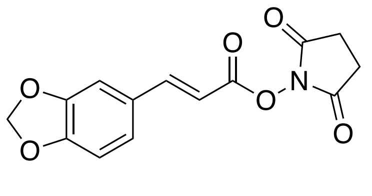

To a flame-dried round bottom flask equipped with a magnetic stirrer bar were added (2*E*)-3-(1,3-benzodioxol-5-yl)-2-propenoic acid (96 mg, 0.5 mmol, 1.0 equiv), EDC·HCl (115 mg, 0.6 mmol, 1.2 equiv), and *N*-hydroxysuccinimide (86 mg, 0.75 mmol, 1.5 equiv). The flask was evacuated and backfilled with N_2 three times prior to the addition of anhydrous CH_2Cl_2 (5.0 mL, 0.1 M) and anhydrous DIPEA (261 μL, 1.5 mmol, 3.0 equiv). The reaction mixture was stirred at room temperature under N_2 for 16 h, then diluted with EtOAc and washed with a saturated aqueous $NaHCO_3$ solution, an aqueous 1 M HCl solution, and a saturated aqueous NaCl solution. The organic layer was dried over Na_2SO_4, filtered over a short silica pad, eluting with EtOAc, and evaporated *in vacuo* to give the product as a white solid (131 mg, 0.45 mmol, 91%). **^{1}H NMR** (400 MHz, $CDCl_3$) δ 7.82 (d, J = 15.9 Hz, 1H), 7.08–7.05 (m, 2H), 6.84 (d, J = 8.5 Hz, 1H), 6.40 (d, J = 16.0 Hz, 1H), 6.04 (s, 2H), 2.87 (br s, 4H); **^{13}C NMR** (101 MHz, $CDCl_3$) δ 169.5, 162.4, 151.0, 149.9, 148.8, 128.1, 126.0, 109.4, 108.9, 106.9, 102.0, 25.8; **HRMS** (ESI-TOF) m/z: $[M + Na]^+$ Calcd for $C_{14}H_{11}NO_6Na$ 312.0479; Found 312.0476; **IR** (neat): 1758, 1733, 1627, 1599, 1504, 1492, 1448, 1423, 1365, 1310, 1265, 1209, 1089, 1069, 1036, 981, 957, 931, 910, 866, 849, 813, 792, 702, 653; **m.p.**: 163–165 °C.

4-(Difluoromethyl)-1-(phenylsulfonyl)piperidine (7)

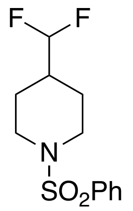

To a flame-dried round bottom flask equipped with a magnetic stirrer bar was added 4-(difluoromethyl)piperidine hydrochloride (172 mg, 1.0 mmol, 1.0 equiv) and the flask was evacuated and backfilled with N_2 three times prior to the addition of anhydrous CH_2Cl_2 (5.0 mL, 0.2 M) and anhydrous NEt_3 (418 µL, 3.0 mmol, 3.0 equiv). Benzene sulfonyl chloride (191 µL, 1.5 mmol, 1.5 equiv) was then added dropwise and the reaction mixture was stirred at room temperature under N_2 for 16 h, then diluted with EtOAc and washed with a saturated aqueous $NaHCO_3$ solution, an aqueous 1 M HCl solution, and a saturated aqueous NaCl solution. The organic layer was dried over Na_2SO_4 and evaporated *in vacuo*. The crude product was purified by silica gel column chromatography (pentane/EtOAc, 100:0 to 70:30) to give the product as a white solid (240 mg, 0.87 mmol, 87%). **^{1}H NMR** (400 MHz, $CDCl_3$) δ 7.79–7.73 (m, 2H), 7.64–7.58 (m, 1H), 7.57–7.50 (m, 2H), 5.55 (td, J = 56.5, 4.5 Hz, 1H), 3.93–3.84 (m, 2H), 2.27 (td, J = 11.9, 2.6 Hz, 2H), 1.87–1.78 (m, 2H), 1.77–1.62 (m, 1H), 1.62–1.48 (m, 2H); **^{19}F NMR** (377 MHz, $CDCl_3$) δ -123.20 (dd, J = 56.7, 12.6 Hz); **^{13}C NMR** (101 MHz, $CDCl_3$) δ 136.3, 133.0, 129.2, 127.7, 117.9 (t, J = 242.0 Hz), 45.5, 39.5 (t, J = 20.5 Hz), 24.4 (t, J = 4.7 Hz); **HRMS** (ESI-TOF) m/z: $[M + H]^+$ Calcd for $C_{12}H_{16}O_2NF_2S$ 276.0864; Found 276.0866; **IR** (neat): 2967, 2360, 2341, 1698, 1477, 1448, 1414, 1393, 1336, 1253, 1165, 1096, 1051, 1039, 988, 947, 926, 861, 759, 742, 690, 668, 647; **m.p.**: 90-93 °C.

***tert*-Butyl 3-(difluoromethyl)azetidine-1-carboxylate (9)**

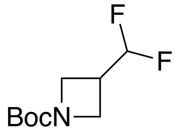

To a flame-dried round bottom flask equipped with a magnetic stirrer bar were added 3-(difluoromethyl)azetidine hydrochloride (72 mg, 0.5 mmol, 1.0 equiv), DMAP (6 mg, 0.05 mmol, 0.1 equiv), and di-*tert*-butyl dicarbonate (164 mg, 0.75 mmol, 1.5 equiv). The flask was evacuated and backfilled with N_2 three times prior to the addition of anhydrous CH_2Cl_2 (2.5 mL, 0.2 M) and anhydrous NEt_3 (139 µL, 1.0 mmol, 2.0 equiv). The reaction mixture was stirred at room temperature under N_2 for 16 h, then washed with a saturated aqueous NaCl solution. The organic layer was dried over Na_2SO_4 and evaporated *in vacuo*. The crude product was purified by silica gel column chromatography (pentane/EtOAc, 100:0 to 90:10) to give the product as a clear oil (67 mg, 0.32 mmol, 65%). **^{1}H NMR** (400 MHz, $CDCl_3$) δ 5.93 (td, J =

56.4, 4.5 Hz, 1H), 4.01 (t, J = 9.1 Hz, 2H), 3.89 (dd, J = 9.2, 5.4 Hz, 2H), 3.00–2.83 (m, 1H), 1.43 (s, 9H); **^{19}F NMR** (377 MHz, $CDCl_3$) δ -124.21 (m); **^{13}C NMR** (101 MHz, $CDCl_3$) δ 156.2, 116.2 (t, J = 240.3 Hz), 80.1, 48.5, 31.7 (t, J = 23.3 Hz), 28.4; **HRMS** (ESI-TOF) m/z: $[M + H]^+$ Calcd for $C_9H_{16}F_2NO_2$ 208.1144; Found 208.1141. All spectroscopic data were in accordance with the literature.[7]

(1*s*,3*r*,5*R*,7*S*)-1-(Difluoromethyl)-3-phenyladamantane (14)

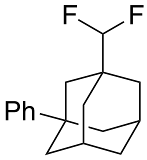

To a falcon tube equipped with a magnetic stirrer was added 2-fluoro-2-((1*s*,3*r*,5*R*,7*S*)-3-phenyladamantan-1-yl)acetic acid (**14a**) (43 mg, 0.15 mmol, 1.0 equiv), followed by anhydrous CH_2Cl_2 (1.5 mL, 0.1 M), and XeF_2 as a solid (51 mg, 0.3 mmol, 2.0 equiv). The reaction mixture was stirred at room temperature for 16 h with the cap sealed loosely. Subsequently, the reaction mixture was washed with an aqueous saturated $NaHCO_3$ solution, dried over Na_2SO_4, evaporated *in vacuo,* and purified by preparative thin layer chromatography (pentane/Et_2O, 99:1) to give the product as a clear film (8 mg, 0.031 mmol, 20%). **^{1}H NMR** (400 MHz, $CDCl_3$) δ 7.39–7.29 (m, 4H), 7.23–7.16 (m, 1H), 5.32 (t, J = 57.1 Hz, 1H), 2.29–2.22 (m, 2H), 1.97–1.83, m, 4H), 1.82–1.63 (m, 8H); **^{19}F NMR** (376 MHz, $CDCl_3$) δ -132.36 (d, J = 56.9 Hz); **^{13}C NMR** (126 MHz, $CDCl_3$) δ 150.0, 128.4, 126.1, 125.0, 120.6 (t, J = 244.3 Hz), 42.6, 40.6 (t, J = 3.4 Hz), 38.3 (t, J = 18.8 Hz), 36.2, 36.1, 34.5 (t, J = 3.6 Hz), 28.3; **HRMS** (ESI-TOF) m/z: $[M + H]^+$ Calcd for $C_{17}H_{21}F_2$ 263.1606; Found 263.1582; **IR** (thin layer film): 2928, 2854, 2363, 2342, 1734, 1601, 1497, 1452, 1387, 1349, 1296, 1165, 1072, 1056, 1002, 971, 841, 755, 718, 699, 670, 637.

6-(4,4-Difluoropiperidin-1-yl)-9-ethyl-9*H*-purine (19)

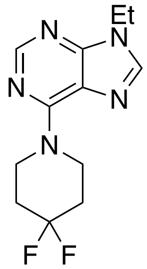

To a flame-dried Schlenk flask equipped with a magnetic stirrer bar were added 6-chloro-9-ethyl-9*H*-purine (55 mg, 0.3 mmol, 1.0 equiv), 4,4-difluoropiperidine hydrochloride (95 mg, 0.6 mmol, 2.0 equiv), and Cs_2CO_3 (293 mg, 0.9 mmol, 3.0 equiv). The flask was evacuated and backfilled with N_2 three times prior to the addition of anhydrous MeCN (3.0 mL, 0.1 M). The reaction mixture was stirred at 80 °C in an oil bath under N_2 for 16 h, then diluted with EtOAc and washed with H_2O and a saturated aqueous NaCl solution. The organic layer was dried over Na_2SO_4 and evaporated *in vacuo*. The crude product was purified by silica gel column chromatography (pentane/EtOAc, 100:0 to 60:40) to give the product as a white solid in quantitative yield. **^{1}H NMR** (400 MHz, $CDCl_3$) δ 8.37 (s, 1H) 7.77 (s, 1H), 4.42 (brs, 4H), 4.24 (q, J = 7.3 Hz, 2H), 2.16–2.00 (m, 4H), 1.51 (t, J = 7.3 Hz, 3H); **^{19}F NMR** (376 MHz, $CDCl_3$) δ -96.92 (p, J = 14.1 Hz); **^{13}C NMR** (151 MHz, $CDCl_3$) δ 153.6, 152.2, 151.0, 138.4, 122.1 (t, J = 242.0 Hz), 120.3, 42.3 (br), 39.0, 34.4 (t, J = 22.9 Hz), 15.6; **HRMS** (ESI-TOF) m/z: $[M + H]^+$ Calcd for $C_{12}H_{16}F_2N_5$ 268.1368; Found 268.1362; **IR** (neat): 2947, 2360, 1590, 1571, 1487, 1466, 1355, 1252, 1144, 1117, 1091, 1059, 937, 902, 804, 790, 744, 647; **m.p.**: 84-86 °C.

(*E*)-3-(Benzo[*d*][1,3]dioxol-5-yl)-1-(4,4-difluoropiperidin-1-yl)prop-2-en-1-one (20)

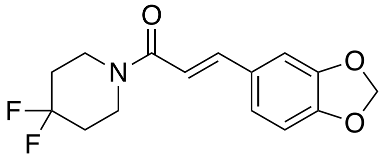

To a flame-dried round bottom flask equipped with a magnetic stirrer bar were added (2*E*)-3-(1,3-benzodioxol-5-yl)-2-propenoic acid (96 mg, 0.5 mmol, 1.0 equiv) and EDC·HCl (115 mg, 0.6 mmol, 1.2 equiv). The flask was evacuated and backfilled with N_2 three times prior to the addition of anhydrous CH_2Cl_2 (5.0 mL, 0.1 M), anhydrous DIPEA (261 µL, 1.5 mmol, 3.0 equiv) and 4,4-difluoropiperidine (83 µL, 0.75 mmol, 1.5 equiv). The reaction mixture was stirred at room temperature under N_2 for 16 h, then diluted with EtOAc and washed with a saturated aqueous $NaHCO_3$ solution, an aqueous 1 M HCl solution, and a saturated aqueous NaCl solution. The organic layer was dried over Na_2SO_4, filtered over a short silica pad, eluting with EtOAc, and evaporated *in vacuo* to give the product as a white solid (117 mg, 0.40 mmol, 79%). **^{1}H NMR** (600 MHz, $CDCl_3$) δ 7.60 (d, J = 15.3 Hz, 1H), 7.03 (d, J = 1.6 Hz, 1H), 7.00 (dd, J = 8.1, 1.7 Hz, 1H), 6.80 (d, J = 8.0 Hz, 1H), 6.70 (d, J = 15.3 Hz, 1H), 5.99 (s, 2H), 3.77

(brs, 4H), 2.07–1.97 (m, 4H); **^{19}F NMR** (565 MHz, $CDCl_3$) δ -97.73 (p, J = 13.8 Hz); **^{13}C NMR** (151 MHz, $CDCl_3$) δ 165.8, 149.4, 148.4, 143.6, 129.5, 124.1, 121.7 (t, J = 242.2 Hz), 114.4, 108.7, 106.5, 101.6, 42.7 (br), 39.3 (br), 35.0 (br), 34.0 (br) (*note*: extra signals observed due to the presence of piperidine rotamers); **HRMS** (ESI-TOF) m/z: $[M + H]^+$ Calcd for $C_{15}H_{16}F_2NO_3$ 296.1093; Found 296.1090; **IR** (neat): 2926, 1645, 1621, 1590, 1501, 1486, 1456, 1432, 1378, 1356, 1254, 1238, 1220, 1120, 1098, 1046, 984, 952, 928, 844, 811, 761, 630; **m.p.**: 107–109 °C.

1-Fluorocyclohex-3-ene-1-carboxylic acid (22a)

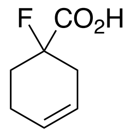

To a 7 mL glass vial equipped with a magnetic stirrer was added butyl 1-fluorocyclohex-3-encarboxylate (70 mg, 0.35 mmol, 1.0 equiv), followed by THF (1.0 mL) and a solution of $LiOH\cdot H_2O$ (88 mg, 2.1 mmol, 6.0 equiv) in H_2O (1.0 mL). The reaction mixture was stirred at room temperature for 16 h, then diluted with H_2O and washed with Et_2O twice. The aqueous layer was acidified to pH ≈ 1 by the addition of an aqueous 6 M HCl solution and extracted with EtOAc three times. The combined organic layers were dried over Na_2SO_4 and evaporated *in vacuo* to give the product as a white solid (45 mg, 0.31 mmol, 89%). **^{1}H NMR** (400 MHz, DMSO-d_6) δ 5.79–5.70 (m, 1H), 5.63–5.55 (m, 1H), 2.63–2.45 (m, 1H), 2.35–2.21 (m, 1H), 2.15–2.05 (m, 2H), 2.03–1.77 (m, 2H); **^{19}F NMR** (376 MHz, DMSO-d_6) δ -160.60 (tdd, J = 36.8, 17.0, 7.3 Hz); **^{13}C NMR** (101 MHz, DMSO-d_6) δ 172.9 (d, J = 25.1 Hz), 126.0, 122.1, 91.9 (d, J = 182.4 Hz), 32.2 (d, J = 24.0 Hz), 28.2 (d, J = 22.9 Hz), 20.6 (d, J = 4.4 Hz); **HRMS** (ESI-TOF) m/z: $[M - H]^-$ Calcd for $C_7H_8FO_2$ 143.0514; Found 143.0505; **IR** (neat): 3181, 3035, 2919, 2849, 1741, 1707, 1658, 1438, 1421, 1401, 1372, 1315, 1269, 1249, 1214, 1082, 972, 940, 880, 832, 773, 727, 649, 632; **m.p.**: 46–48 °C.

Radiochemistry

General experimental details

For manual radiochemistry experiments:

[^{18}F]Fluoride was produced by Alliance Medical (UK), Invicro (UK) or PETIC (UK) *via* the ^{18}O(p,n)^{18}F reaction and delivered as [^{18}F]fluoride in [^{18}O]water. Radiosynthesis and azeotropic drying were performed on a NanoTek® automated microfluidic device (Advion) or AllinOne synthesizer (TRASIS).

For automated radiochemistry experiments:

[^{18}F]Fluoride was produced in an IBA Cyclon 18/9 cyclotron using the ^{18}O(p,n)^{18}F reaction. All experiments were performed on a TRASIS AllinOne radiosynthesizer (TRASIS).

HPLC eluent systems and columns:

Analytical HPLC runs were performed either with a Dionex Ultimate 3000 dual channel HPLC system equipped with shared autosampler, parallel UV-detectors and LabLogic NaI/PMT-radiodetectors with Flow-RAM analog output (Conditions A, B, approximate radio-UV detector offset = 0.1 min (pump 1) or 0.3 min (pump 2)), or on an Agilent 1200 equipped with a UV detector and LabLogic gamma-RAM Model 4 detector (Conditions C: approximate radio-UV detector offset = 0.1 min). Semi-preparative purification of radiolabeled products was achieved using the integrated HPLC system (including UV and radio detectors) of a Trasis AllInOne synthesizer (conditions D).

Conditions A: analytical
Flow rate = 1.0 mL/min; temperature = 25 °C; wavelength = 220 nm (unless otherwise specified); column: Phenomenex Gemini 5 μm C18 110 Å 250 x 4.6 mm; HPLC gradient: H_2O/MeCN, 0-5 min (5% MeCN) isocratic, 5-7.5 min (5% MeCN to 95% MeCN) linear increase, 7.5-14.5 min (95% MeCN) isocratic, 14.5-15.5 min (95% MeCN to 5% MeCN) linear decrease, 15.5-18 min (5% MeCN) isocratic.

Conditions B: analytical

Flow rate = 1.0 mL/min; temperature = 25 °C; wavelength = 220 nm (unless otherwise specified); column: Phenomenex Synergi^{TM} 4 µm Hydro RP 80 Å 150 x 4.6 mm LC column; HPLC gradient: H_2O/MeCN, 0-1 min (25% MeCN) isocratic, 1-10 min (25% MeCN to 95% MeCN) linear increase, 10-14 min (95% MeCN) isocratic, 14-17 min (95% MeCN to 25% MeCN) linear decrease, 17-19.4 min (25% MeCN) isocratic.

Conditions C: analytical

Flow rate = 1.0 mL/min; temperature = 25 °C; wavelength = 220 nm (unless otherwise specified); column: Agilent C18 Eclipse Plus 80Å 150 x 4.6 mm LC column; HPLC gradient: H_2O/MeCN, 0-1 min (25% MeCN) isocratic, 1-10 min (25% MeCN to 95% MeCN) linear increase, 10-16 min (95% MeCN) isocratic, 16-18 min (95% MeCN to 25% MeCN) linear decrease, 18-20 min (25% MeCN) isocratic.

Conditions D: semi-preparative purification

Flow rate = 4.0 mL/min; temperature = room temperature; wavelength = 200 nm; column: Phenomenex Gemini 5 µm 250 x 10 mm LC column; isocratic: water/MeCN.

Optimisation of reaction conditions

Procedure for the ^{18}F-fluorodecarboxylation of **1**

Manual [^{18}F]KF elution and drying: [^{18}F]Fluoride was separated from ^{18}O-enriched-water using an anion exchange cartridge (Waters Sep-Pak AccellPlus QMA Carbonate Plus Light Cartridge), activated with H_2O (10 mL) prior to use and released with a solution of Et_4NHCO_3 (9 mg) in MeCN (0.8 mL) and H_2O (0.2 mL), which was concentrated over a period of 20 min by azeotropic drying using dry MeCN (3 x 0.7 mL) under a flow of N_2 at 110 °C.

^{18}F-fluorination: An oven-dried 3 mL v-vial equipped with a magnetic stirrer bar and capped with a septum was charged with Mn(tmp)Cl, PhIO, and 1-benzoyl-4-fluoropiperidine-4-carboxylic acid (7.5 mg, 0.03 mmol). [^{18}F]TEAF (10-50 MBq) in MeCN (approximately 50 μL) was subsequently added to the vial and MeCN was removed by drying under a flow of N_2 at 80 °C. Solvent (0.3 mL) was then added to the vial and the reaction mixture was stirred at 80 °C for 20 min. The reaction mixture was then diluted with EtOH/H_2O (0.3 mL, 9:1 v/v) and an aliquot was filtered and analyzed by radioHPLC. The radiochemical yield (RCY) was determined by integration of the ^{18}F-product relative to the total peak area for all radioactive species observed.

Table s1: Optimisation of reaction conditions for the ^{18}F-fluorodecarboxylation of **1**.

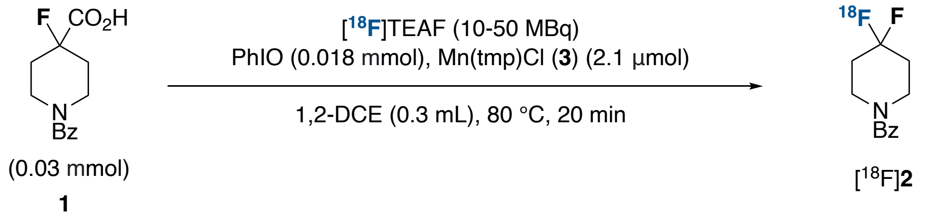

Entry	Deviation from standard conditions	RCY (%)
**1**	none	46 ± 14 $_{n = 4}$
**2**	DMF as solvent	44% $_{n = 1}$
**3**	$CHCl_3$ as solvent	40% $_{n = 1}$
**4**	1,4-dioxane as solvent	36% $_{n = 1}$
**5**	50 °C instead of 80 °C	27% $_{n = 1}$
**6**	9 µmol PhIO	16% $_{n = 1}$
**7**	36 µmol PhIO	49% $_{n = 1}$
**8**	no PhIO	traces $_{n = 1}$
**9**	Mn(tmp)OTf instead of **3**	40% $_{n = 1}$
**10**	1 µmol **3**	32% $_{n = 1}$
**11**	4 µmol **3**	26% $_{n = 1}$
**12**	no Mn(tmp)Cl	traces $_{n = 1}$
**13**	1 min reaction time	47% $_{n = 1}$

Robustness screen

Procedure for the ^{18}F-fluorodecarboxylation of α-fluoro carboxylic acids

Manual [^{18}F]KF elution and drying: [^{18}F]Fluoride was separated from ^{18}O-enriched-water using an anion exchange cartridge (Waters Sep-Pak AccellPlus QMA Carbonate Plus Light Cartridge), activated with H_2O (10 mL) prior to use and released with a solution of Et_4NHCO_3 (9 mg) in MeCN (0.8 mL) and H_2O (0.2 mL), which was concentrated over a period of 20 min by azeotropic drying using dry MeCN (3 x 0.7 mL) under a flow of N_2 at 110 °C.

^{18}F-fluorination: An oven-dried 3 mL v-vial equipped with a magnetic stirrer bar and capped with a septum was charged with Mn(tmp)Cl (1.8 mg, 2.1 μmol), PhIO (4 mg, 0.018 mmol), and 1-benzoyl-4-fluoropiperidine-4-carboxylic acid (7.5 mg, 0.03 mmol). [^{18}F]TEAF (10-50 MBq) in MeCN (approximately 50 μL) was subsequently added to the vial and MeCN was removed by drying under a flow of N_2 at 80 °C. 1,2-DCE (0.3 mL) and additive (0.03 mmol) were then added to the vial and the reaction mixture was stirred at 80 °C for 20 min. The reaction mixture was then diluted with EtOH/H_2O (0.3 mL, 9:1 v/v) and an aliquot was filtered and analyzed by radioHPLC. The radiochemical yield (RCY) was determined by integration of the ^{18}F-product relative to the total peak area for all radioactive species observed.

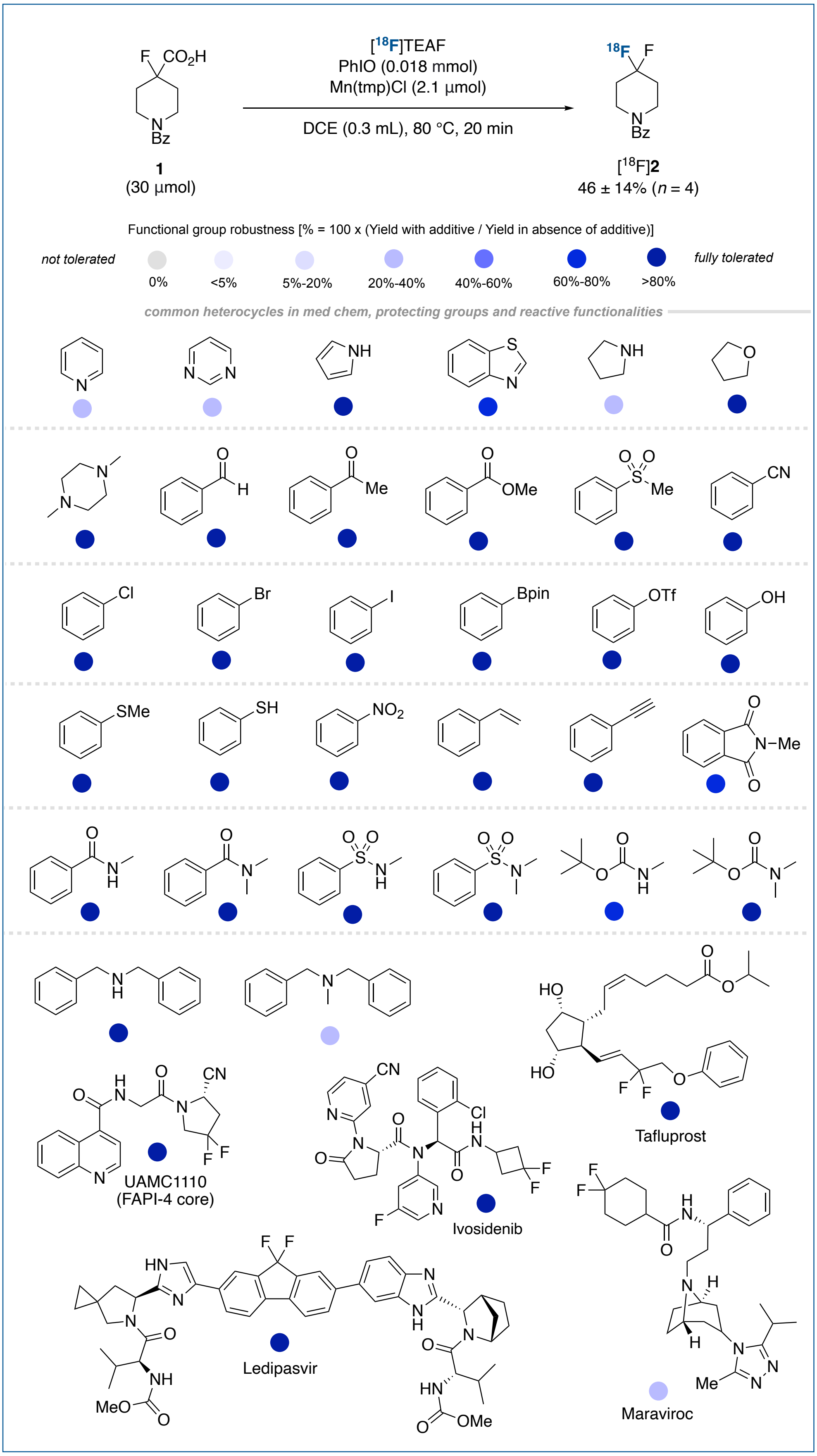

Figure s1: Outcome of the robustness screen expressed as colour-coded robustness factor for each additive.

Radiotrace overlays and radiochemical yields

General procedure for the ^{18}F-fluorodecarboxylation of α-fluoro carboxylic acids

Manual [^{18}F]KF elution and drying: [^{18}F]Fluoride was separated from ^{18}O-enriched-water using an anion exchange cartridge (Waters Sep-Pak AccellPlus QMA Carbonate Plus Light Cartridge), activated with H_2O (10 mL) prior to use and released with a solution of Et_4NHCO_3 (9 mg) in MeCN (0.8 mL) and H_2O (0.2 mL), which was concentrated over a period of 20 min by azeotropic drying using dry MeCN (3 x 0.7 mL) under a flow of N_2 at 110 °C.

^{18}F-fluorination: An oven-dried 3 mL v-vial equipped with a magnetic stirrer bar and capped with a septum was charged with Mn(tmp)Cl (1.8 mg, 2.1 μmol), PhIO (4 mg, 0.018 mmol), and carboxylic acid substrate (0.03 mmol). [^{18}F]TEAF (10-50 MBq) in MeCN (approximately 50 μL) was subsequently added to the vial and MeCN was removed by drying under a flow of N_2 at 80 °C. 1,2-DCE (0.3 mL) was then added to the vial and the reaction mixture was stirred at 80 °C for 20 min. The reaction mixture was then diluted with EtOH/H_2O (0.3 mL, 9:1 v/v) and an aliquot was filtered and analyzed by radioHPLC. The radiochemical yield (RCY) was determined by integration of the ^{18}F-product relative to the total peak area for all radioactive species observed.

[18F](4,4-Difluoropiperidin-1-yl)(phenyl)methanone ([18F]2)

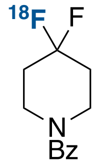

Entry	RCY (%)
**1**	55
**2**	63
**3**	40
**4**	26

Average RCY: 46 ± 14% (*n* = 4)

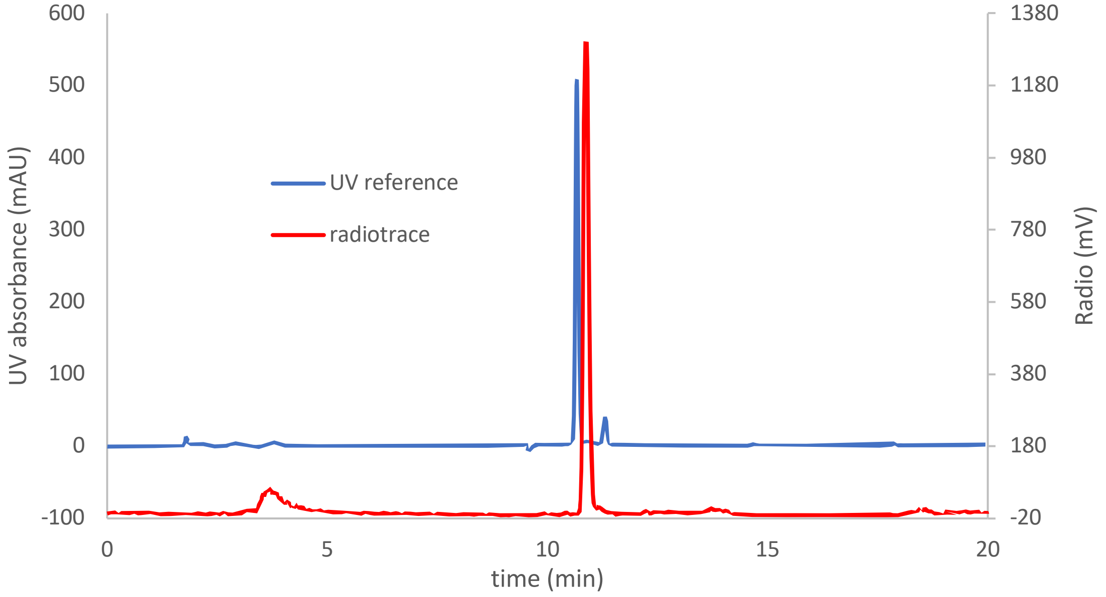

HPLC conditions A

[^{18}F]*tert*-Butyl 4,4-difluoropiperidine-1-carboxylate ([^{18}F]4)

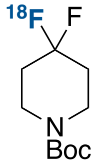

Entry	RCY (%)
**1**	67
**2**	34
**3**	36
**4**	29

Average RCY: 42 ± 15% (n = 4)

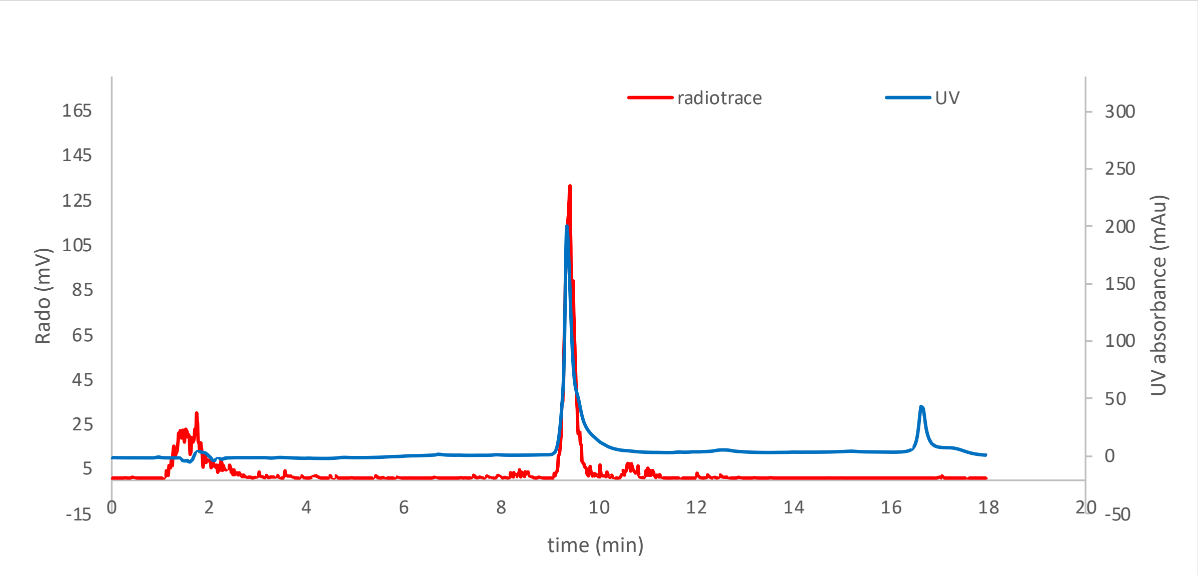

Note: for this run, the wavelength was adjusted to 207 nm.

HPLC conditions B

[18F]*tert*-Butyl 3,3-difluoropiperidine-1-carboxylate ([18F]6)

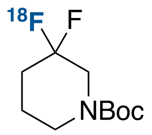

Entry	RCY (%)
**1**	38
**2**	44
**3**	42

Average RCY: 41 ± 3% (n = 3)

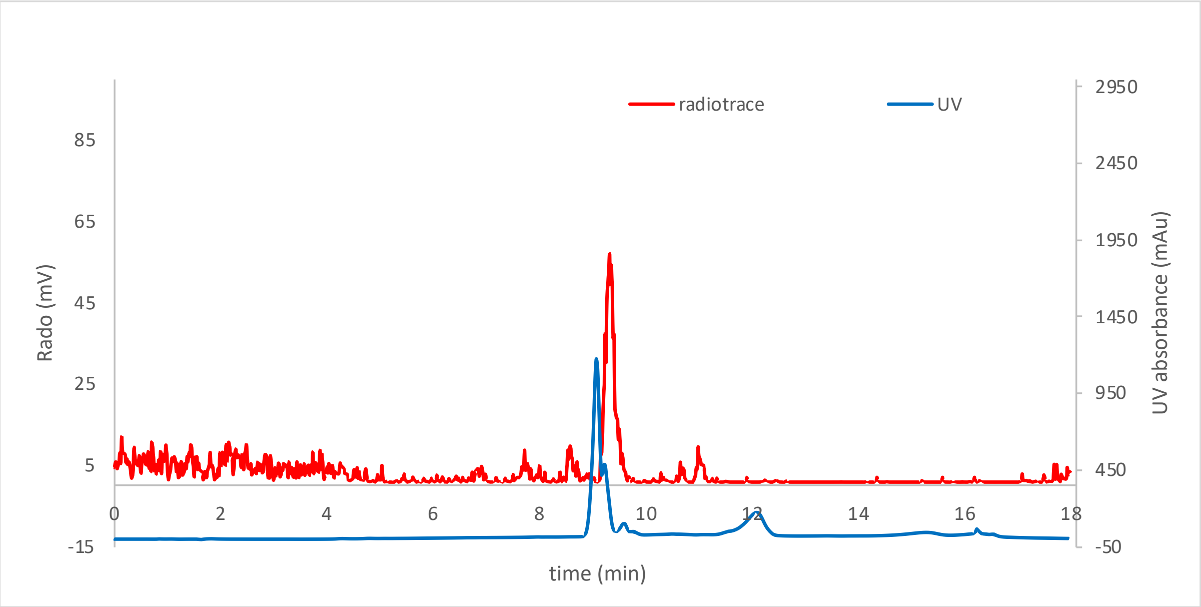

Note: for this run, the wavelength was adjusted to 200 nm.

HPLC conditions B

[^{18}F]4-(Difluoromethyl)-1-(phenylsulfonyl)piperidine ([^{18}F]7)

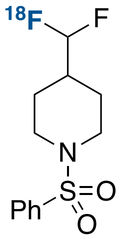

Entry	RCY (%)
**1**	51
**2**	52
**3**	52

Average RCY: 52 ± 1% (n = 3)

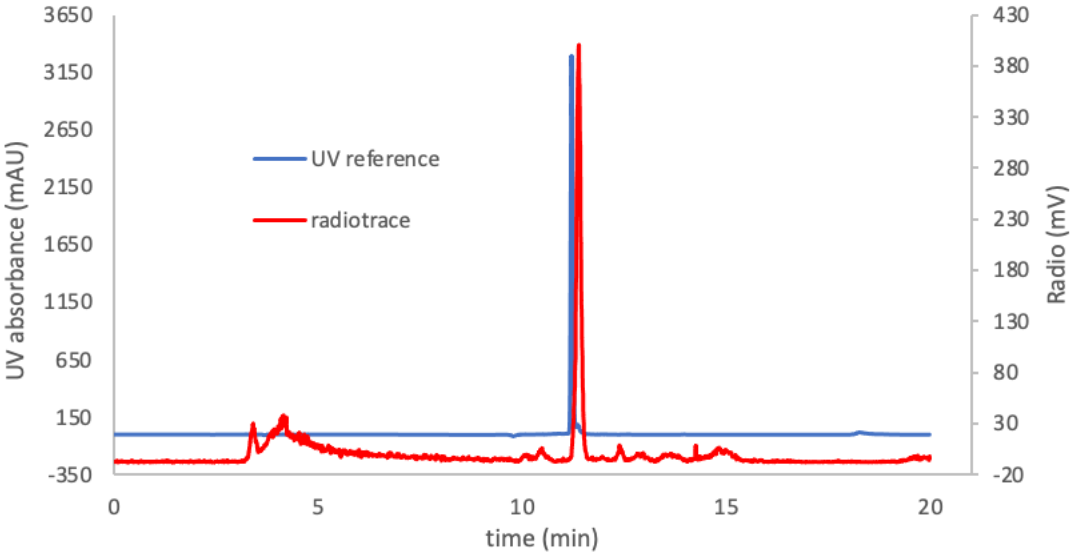

HPLC conditions A

[^{18}F]*tert*-Butyl 3,3-difluoropyrrolidine-1-carboxylate ([^{18}F]8)

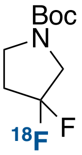

Entry	RCY (%)
**1**	20
**2**	20
**3**	10

Average RCY: 17 ± 5% (n = 3)

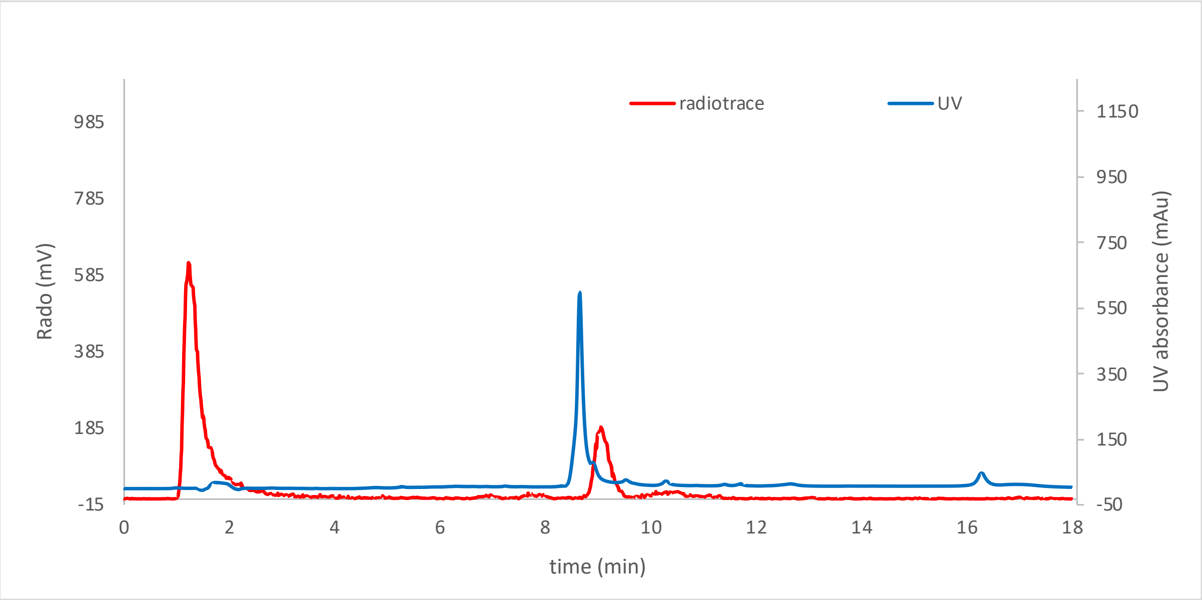

Note: for this run, the wavelength was adjusted to 200 nm.

HPLC conditions B

[^{18}F]*tert*-Butyl 3-(difluoromethyl)azetidine-1-carboxylate ([^{18}F]9)

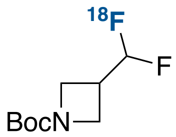

Entry	RCY (%)
1	12
2	8
3	5

Average RCY: 8 ± 3% (n = 3)

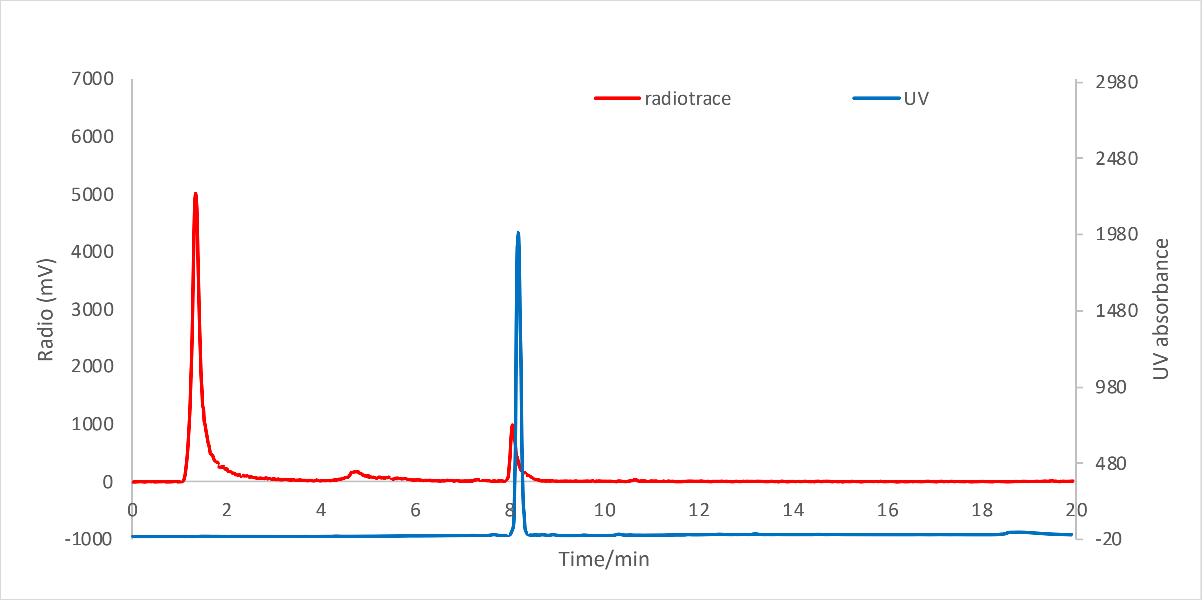

HPLC conditions C

[^{18}F]1-Chloro-4-(4,4-difluorocyclohexyl)benzene ([^{18}F]10)

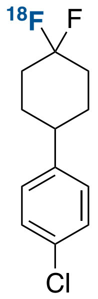

Entry	RCY (%)
**1**	39
**2**	37
**3**	46

Average RCY: 41 ± 4% (n = 3)

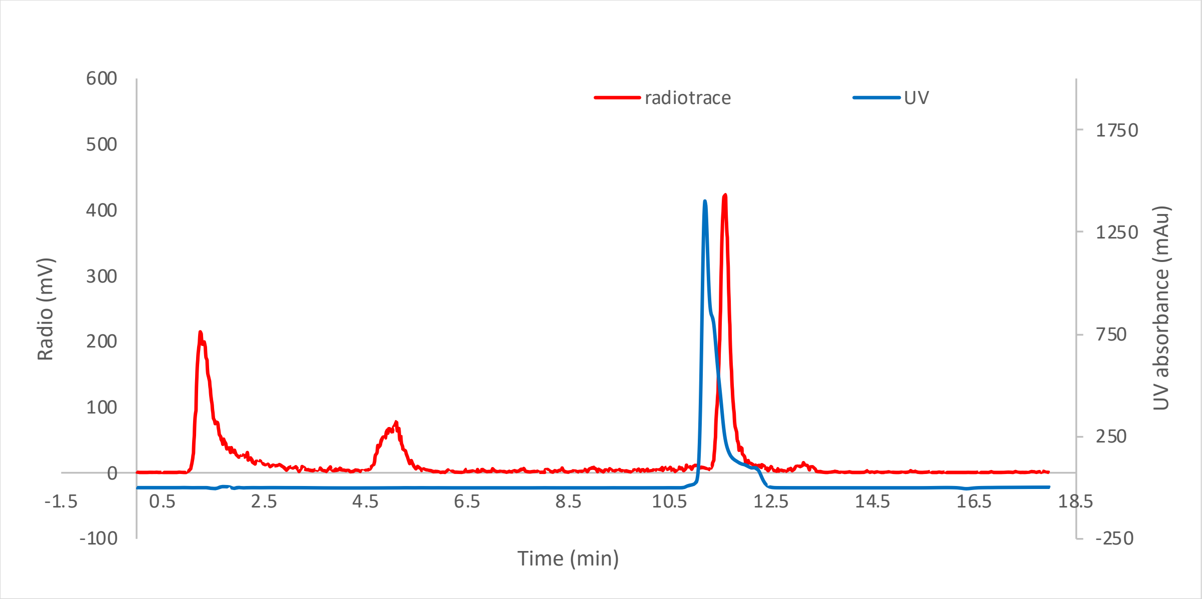

HPLC conditions B

[^{18}F](5,5-Difluoropentyl)benzene ([^{18}F]11)

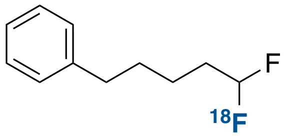

Entry	RCY (%)
**1**	12
**2**	10
**3**	9

Average RCY: 10 ± 1% (n = 3)

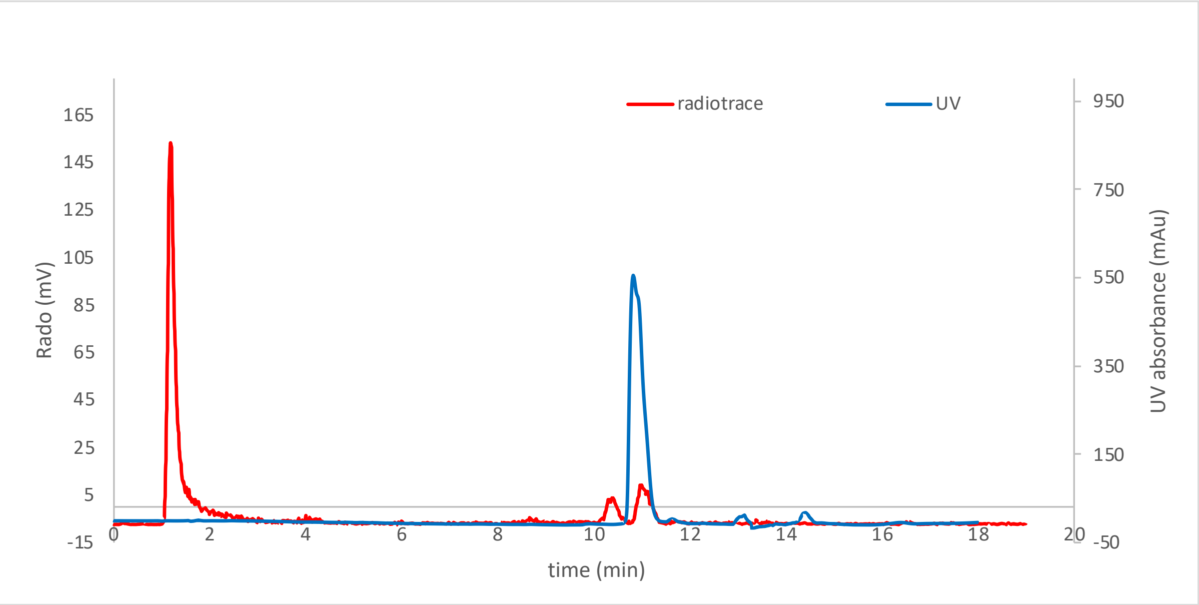

HPLC conditions B

[^{18}F](2,2-difluoropropyl)benzene ([^{18}F]12)

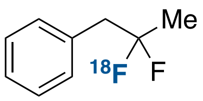

Entry	RCY (%)
1	9
2	20
3	36

Average RCY: 22 ± 11% (n = 3)

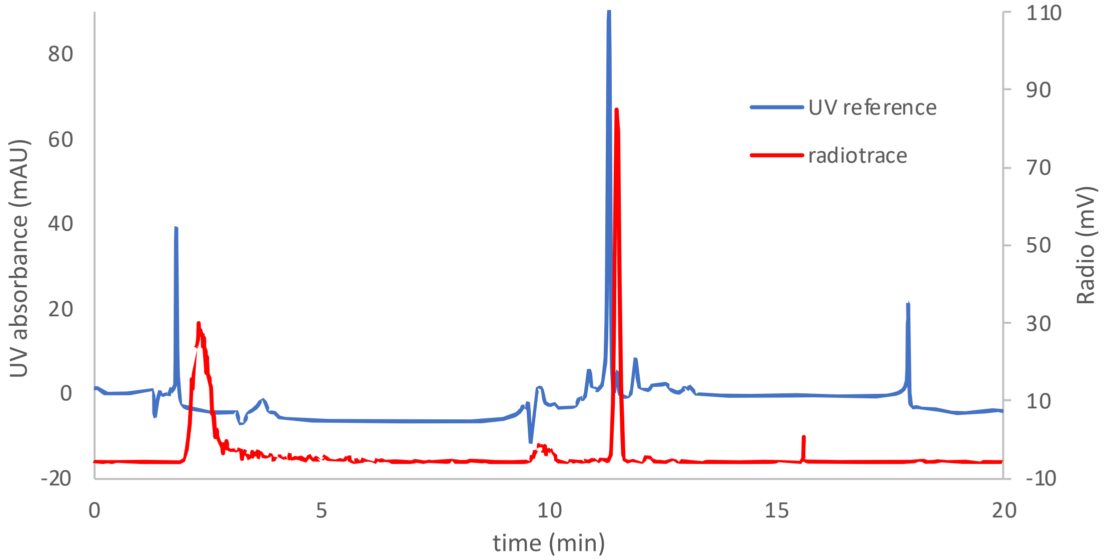

HPLC conditions A

[^{18}F]2-(3,3-Difluorobutyl)isoindoline-1,3-dione ([^{18}F]13)

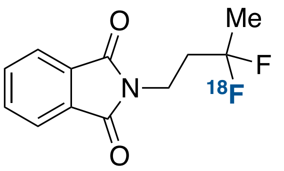

Entry	RCY (%)
**1**	40
**2**	32
**3**	59

Average RCY: 44 ± 11% (n = 3)

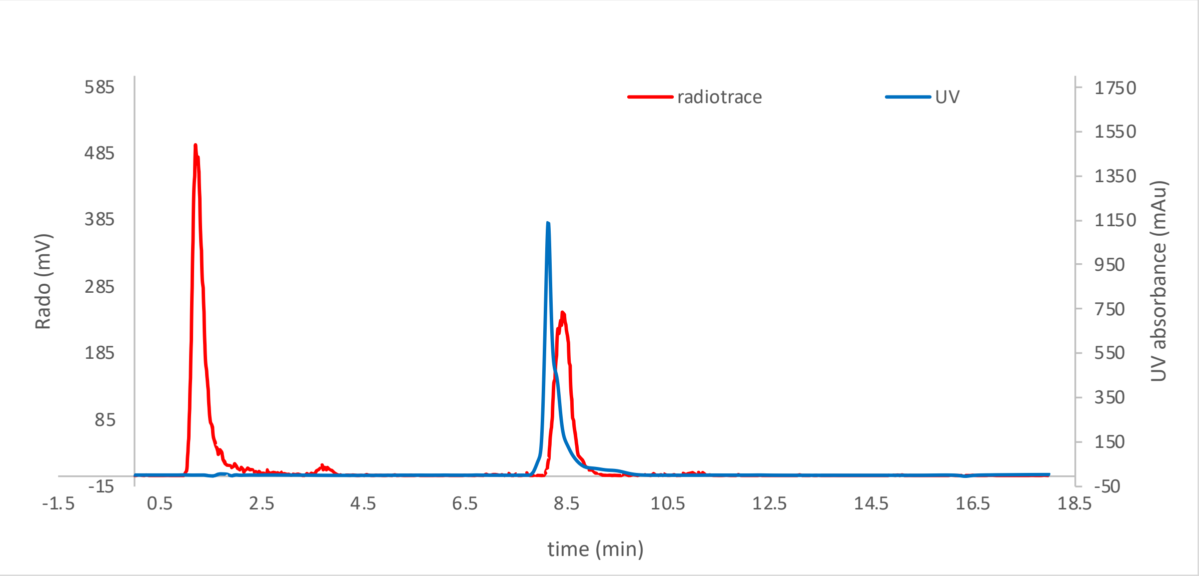

HPLC conditions B

[^{18}F](1*s*,3*r*,5*R*,7*S*)-1-(Difluoromethyl)-3-phenyladamantane ([^{18}F]14)

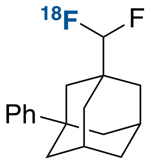

Entry	RCY (%)
**1**	34
**2**	37
**3**	43

Average RCY: 38 ± 4% (n = 3)

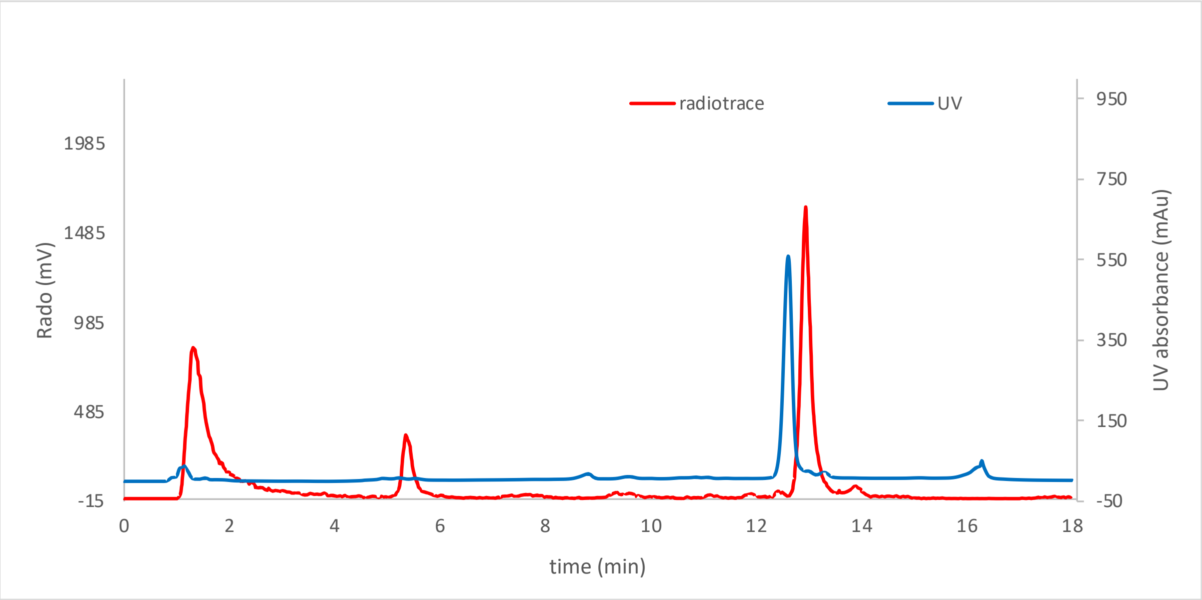

HPLC conditions B

[^{18}F]9,9-Difluoro-9*H*-fluorene ([^{18}F]15)

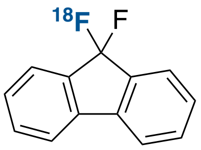

Entry	RCY (%)
**1**	30
**2**	10
**3**	20

Average RCY: 20 ± 8% (n = 3)

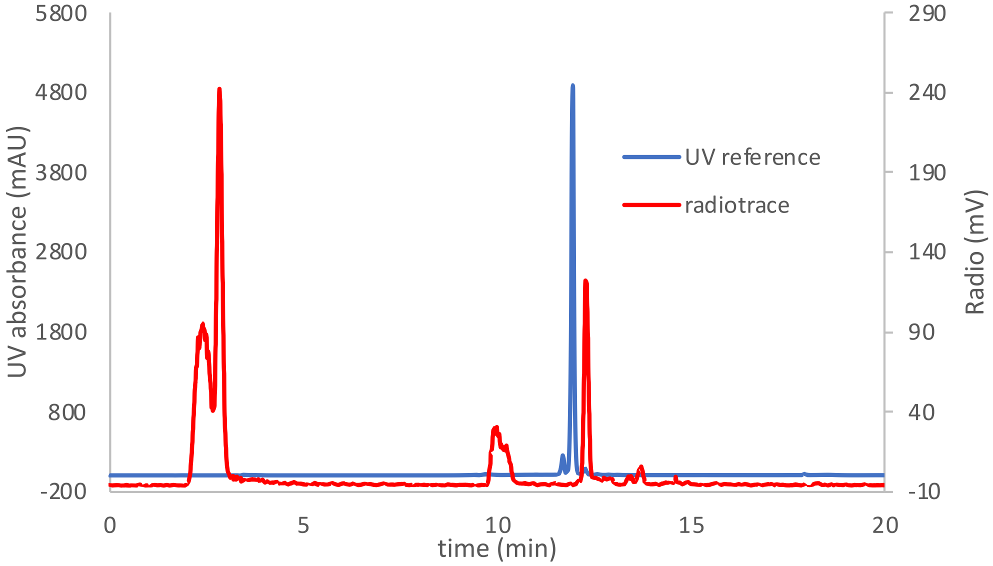

HPLC conditions A

[^{18}F]1,2-Dimethyl-4-(trifluoromethyl)benzene ([^{18}F]16)

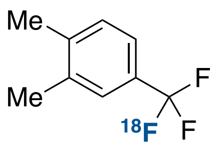

Entry	RCY (%)
**1**	19
**2**	22
**3**	30

Average RCY: 24 ± 5% (n = 3)

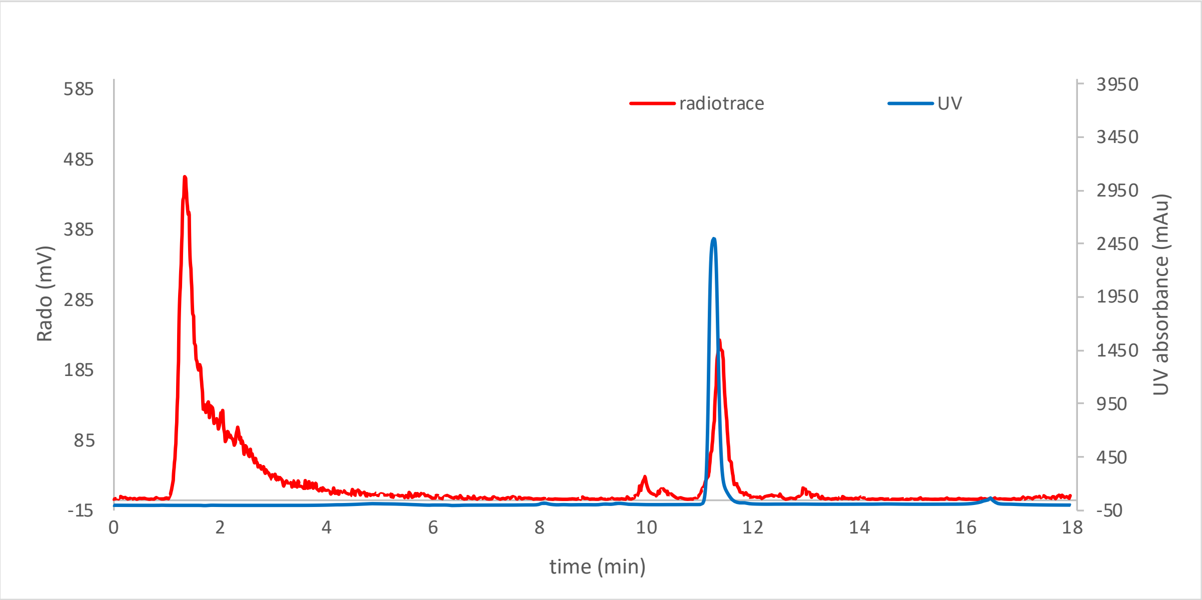

HPLC conditions B

Automation

The automated radiosynthesis of [^{18}F]**4** was performed with a Trasis AllInOne synthesizer using an automated program and pre-built cassette (**Table s2**, **Figure s2**). The vial in slot 2 was charged with a solution of Mn(tmp)Cl (1.8 mg, 2.1 μmol) in MeOH (1.0 mL). The vial in slot 9 was charged with 1-(*tert*-butoxycarbonyl)-4-fluoropiperidine-4-carboxylic acid (7.4 mg, 0.03 mmol), PIDA (5.8 mg, 0.018 mmol) and anhydrous 1,2-DCE (1.0 mL). The solvent reservoirs (slots 8 and 17) were filled with anhydrous MeCN (ca. 10 mL) and MeCN/H_2O (1:1 v/v, ca. 10 mL) (slot 10). The Waters Sep-Pak AccellPlus QMA Carbonate Plus Light Cartridge (slot 5) was activated with water (10 mL) prior to use. After [^{18}F]fluoride in [^{18}O]water was received from the cyclotron, it was trapped on a Waters Sep-Pak AccellPlus QMA Carbonate Plus Light cartridge. [^{18}F]Fluoride was then eluted with a solution of Mn(tmp)Cl (slot 2), which was taken up in a 3mL syringe (slot 3) and pushed through the QMA cartridge. [^{18}F]Fluoride was then dried under a flow of air at 120 °C. Once drying was complete, the reagent mixture (slot 9) was taken up in a 3 mL syringe (slot 11) and transferred to the reactor and the reaction mixture was heated at 80 °C for 10 min, then the reaction dried under a flow of air at 65 °C for 10 min. A solution of MeCN/H_2O (4 mL) was then taken up in a 3 mL syringe (slot 11) and transferred to the reactor. The resulting mixture was taken up in a 3 mL syringe (slot 11) and transferred back to the reactor for mixing. This step was repeated twice. The crude reaction mixture was then finally taken up in a 3 mL syringe (slot 11), loaded onto the HPLC from slot 12 and purified by reverse-phase semi-preparative HPLC (HPLC conditions D, MeCN/H_2O = 55/:45 (v/v) as eluent, Q = 4 ml/min, t_R([^{18}F]**4**) = 13-15 min) (**Figure s3**). The eluate was collected from slot 36 and diluted with water (20 mL) (slot 34). This mixture was then taken up in a 20 mL syringe (slot 15) and loaded as a 1:1 mixture with additional water (taken up in the syringe from the water bag at slot 14) onto a Waters Sep-Pak C18 Plus cartridge (slot 33) (preconditioned with EtOH (10 mL), then water (10 mL). Water (10 mL) from the water bag (slot 14) was taken up in a 20 mL syringe (slot 15) and pushed through the Waters Sep-Pak C18 Plus cartridge (slot 33), which was then purged with a flow of air. MeCN (2 mL) (slot 17) was then taken up in a 20 mL syringe (slot 15) and pushed through the Waters Sep-Pak C18 Plus cartridge (slot 33). The eluted radiofluorinated product was collected in a vial connected to slot 31. The activity of the collected product was measured in a dose calibrator. An aliquot (ca. 20 MBq) of the isolated product was analyzed by radio-HPLC to determine molar activity. HPLC analysis was performed under HPLC conditions C at 200 nm wavelength. The molar

activity was determined on a chemically and radiochemically pure sample. The same sample was injected in triplicate, with the injected activity and time of injection recorded. The UV response corresponding to the desired radiofluorinated product was then integrated, to give the amount of non-radioactive product that was detected. Molar activity was then calculated. Radiosynthesis data are summarized below (**Table s3**).

Table s2:

Entry	Slot	Item
**1**	1 side	line to TRASIS AllinOne (pressure)
**2**	1 top	line to target water recovery vial
**3**	2	QMA eluent (Mn(tmp)Cl (1.8 mg) in 1.0 mL MeOH)
**4**	3	3 mL syringe
**5**	4	line to Waters Sep-Pak AccellPlus QMA Carbonate Plus Light Cartridge (slot 5)
**6**	5	Waters Sep-Pak AccellPlus QMA Carbonate Plus Light Cartridge
**7**	6	activity plunger
**8**	7	line to reactor
**9**	8	anhydrous MeCN (10 mL)
**10**	9	reagent mixture (1-(*tert*-butoxycarbonyl)-4-fluoropiperidine-4-carboxylic acid (7.4 mg) and PIDA (5.8 mg) in anhydrous 1,2-DCE (1.0 mL)
**11**	10	quench solution (10 mL, MeCN/H_2O 1:1 v/v)
**12**	11	3 mL syringe
**13**	12	line to Trasis AllinOne (HPLC load)
**14**	13	empty
**15**	14	line to water bag
**16**	15	20 mL syringe
**17**	16	line to slot 35
**18**	17	anhydrous MeCN (10 mL)
**19**	18 top	line to TRASIS AllinOne (pressure)
**20**	18 side	line to TRASIS AllinOne (vacuum)
**21**	25 top	line to reactor
**22**	25 side	line to TRASIS AllinOne (vacuum)
**23**	26	empty
**24**	27	empty
**25**	28	empty
**26**	29	empty
**27**	30	empty
**28**	31	line to product collection vial
**29**	32	line to Waters Sep-Pak C18 Plus cartridge (slot 33)
**30**	33	Waters Sep-Pak C18 Plus cartridge
**31**	34	H_2O (20 mL)
**32**	35	line to position 16
**33**	36 top	line to TRASIS AllinOne (HPLC collect)
**34**	36 side	line to TRASIS AllinOne (pressure)

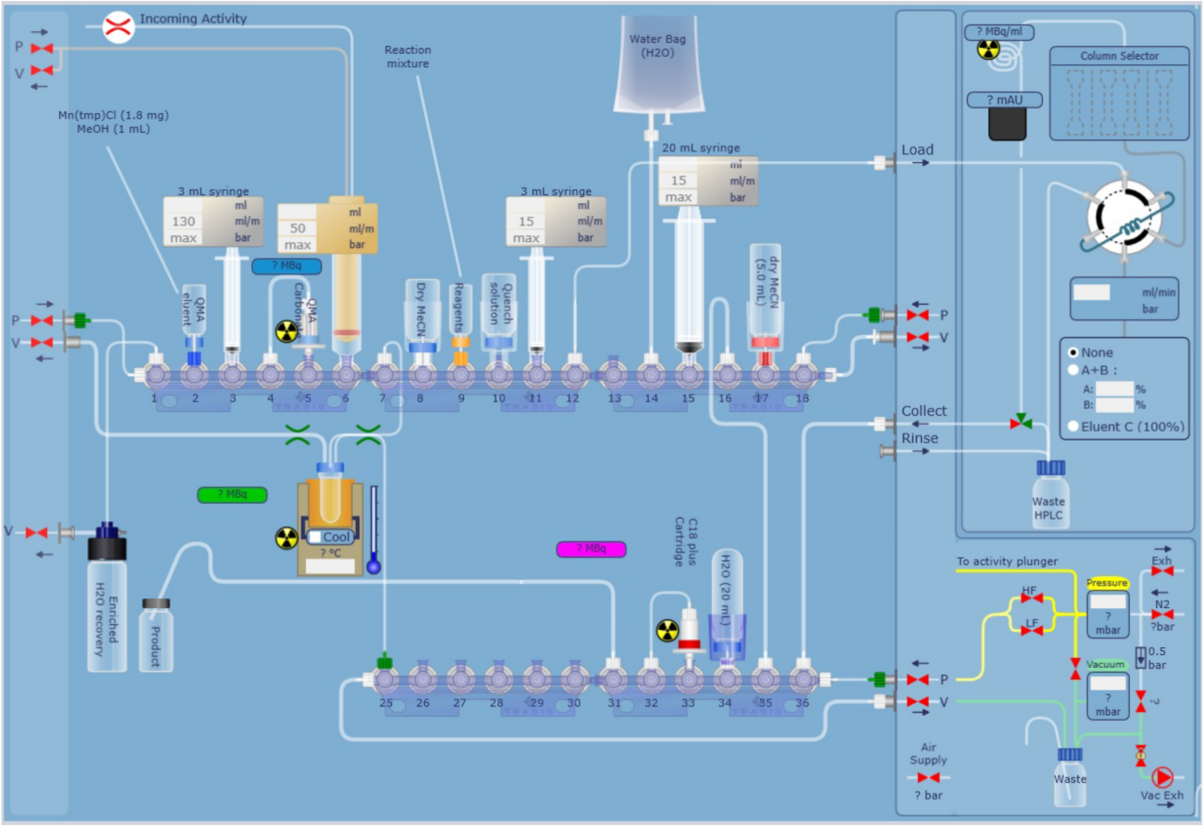

Figure s2: Cassette set-up for the automated ^{18}F-fluorodecarboxylation of 1-(*tert*-butoxycarbonyl)-4-fluoropiperidine-4-carboxylic acid using a Trasis AllinOne automated platform.

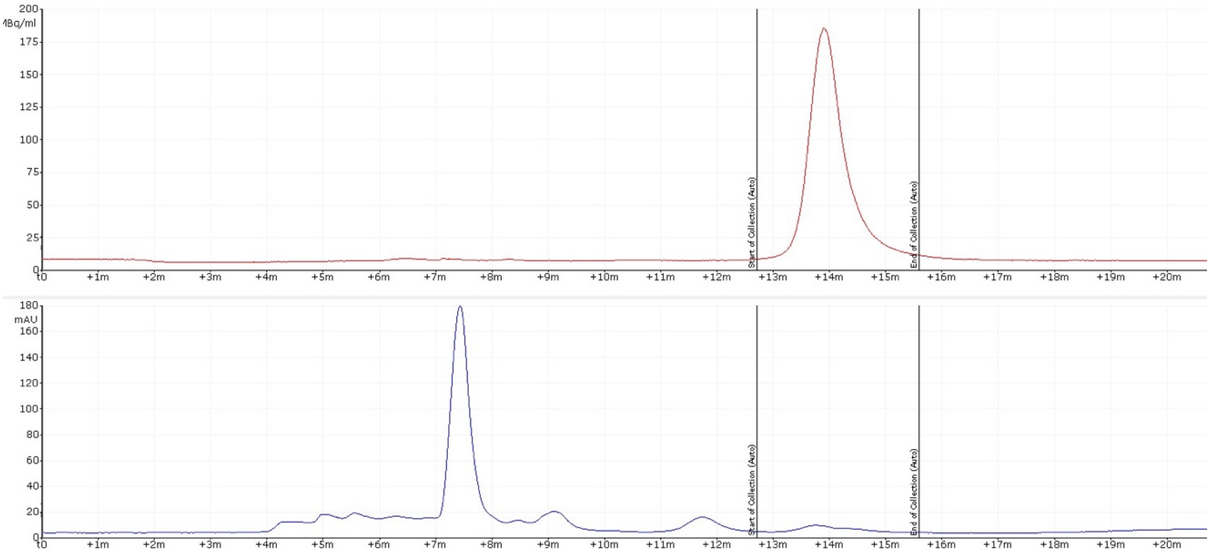

Figure s3: RadioHPLC chromatogram of the crude reaction mixture.

Table s3: Radiosynthesis of [^{18}F]**4**

Starting activity	AY	Radiosynthesis time	RCP	A_m
50 GBq	1.736 GBq	78 min	> 99%	6.35 GBq/µmol (d.c. EOS)

Procedure for the calibration curve of *tert*-butyl 4,4-difluoropiperidine-1-carboxylate (4): A calibration curve for authentic reference **4** was recorded by preparing samples of a range of concentrations by serial dilution, starting with a solution of **4** (2.0 mg) in MeCN (1.0 mL) (**Figure s4**). These were injected onto an HPLC (10 µL injection volume from a 1.0 mL stock, HPLC conditions C, 190 nm wavelength) and the UV response was measured by integrating the peak of interest.

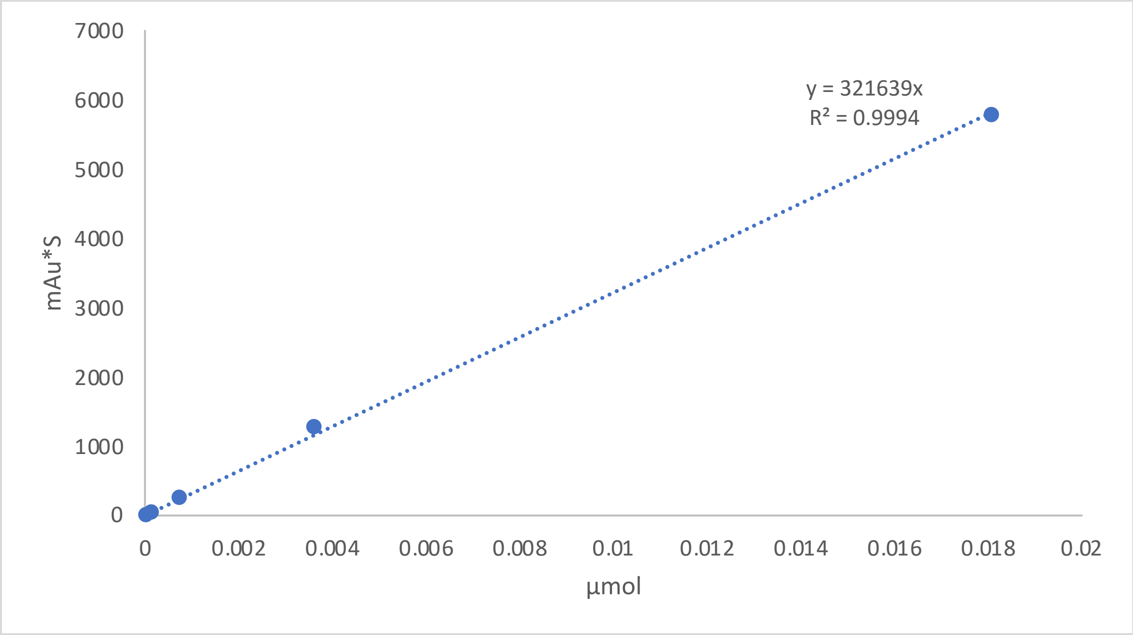

Figure s4: HPLC calibration curve for authentic reference *tert*-butyl 4,4-difluoropiperidine-1-carboxylate (**4**)

Molar activity was then calculated. These data are summarized in **Table s4**.

Table s4: Molar activity calculation of **[^{18}F]4**

Measurement	Activity injected (GBq, d.c.)	Peak area (mAu*s)	4 injected (µmol)	A_m (GBq/µmol)
1	2.740382 x 10^{-4}	12.4	3.855 x 10^{-5}	7.10
2	4.806043 x 10^{-4}	25.6	7.959 x 10^{-5}	6.04
3	3.998905 x 10^{-4}	21.8	6.778 x 10^{-5}	5.90

Average A_m: 6.35 ± 0.54 GBq/µmol (d.c. EOS)

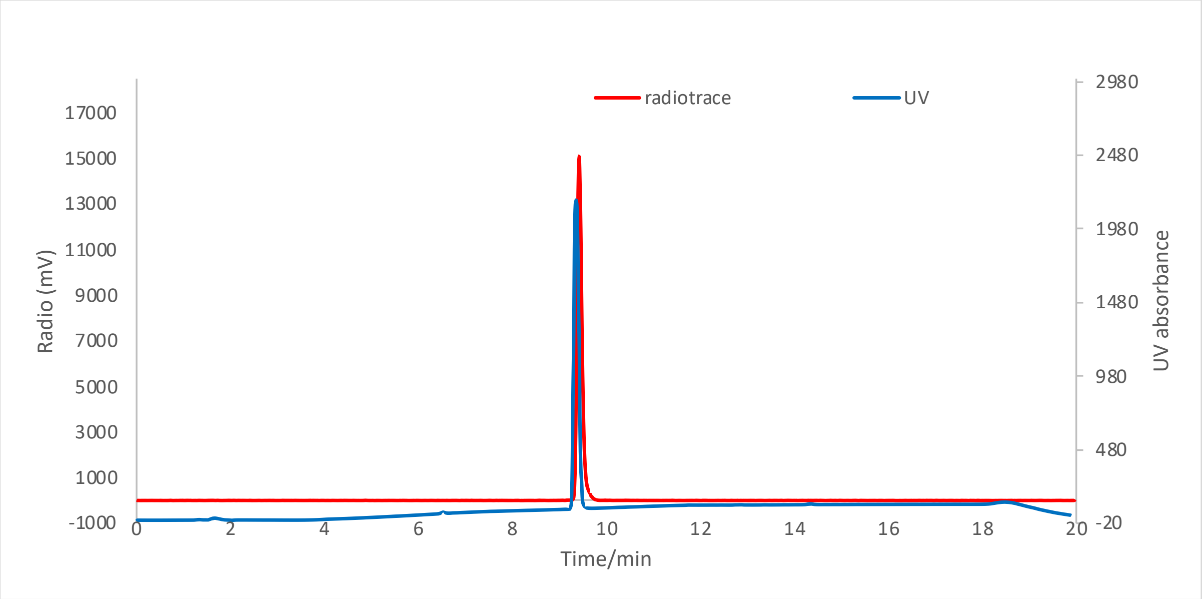

Note: for this run, the wavelength was adjusted to 190 nm.

HPLC conditions C

The automated radiosynthesis of [^{18}F]**4** was repeated. Radiosynthesis data are summarized below (**Tables s5**, **s7**).

Table s5: Radiosynthesis of [^{18}F]**4**

Starting activity	AY	Radiosynthesis time	RCP	A_m
10 GBq	861 MBq	78 min	> 99%	2.27 GBq/µmol (d.c. EOS)
10 GBq	499 MBq	78 min	> 99%	n.d.

Average AY: 680 ± 181 MBq (n = 2)

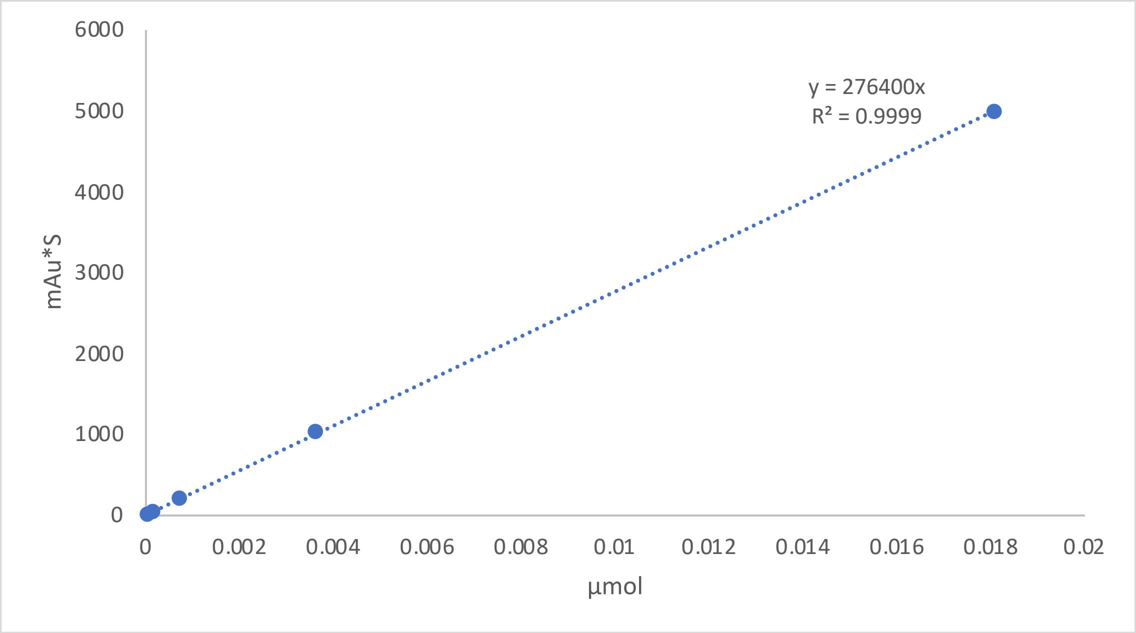

Figure s5: HPLC calibration curve for authentic reference *tert*-butyl 4,4-difluoropiperidine-1-carboxylate (**4**)

Table s6: Molar activity calculation of **[^{18}F]4**

Measurement	Activity injected (MBq, d.c.)	Peak area (mAu*s)	4 injected (µmol)	A_m (GBq/µmol)
1	0.9647	104.7	0.0003788	2.55
2	0.7390	103.4	0.0003741	1.98
3	0.8491	102.4	0.00037048	2.29

Average A_m: 2.27 ± 0.23 GBq/µmol (d.c. EOS)

Table s7: Radiosynthesis of [^{18}F]**4**

Starting activity	AY	Radiosynthesis time	RCP	A_m
5 GBq	328 MBq	80 min	> 99%	n.d.

The automated radiosynthesis of [^{18}F]**4** was also repeated with a lower substrate loading and reaction temperature to investigate the impact on A_m. Hence, the vial in slot 2 was charged with a solution of Mn(tmp)Cl (1.2 mg, 1.4 µmol) in MeOH (1.0 mL). The vial in slot 9 was charged with 1-(*tert*-butoxycarbonyl)-4-fluoropiperidine-4-carboxylic acid (5.0 mg, 0.02 mmol), PIDA (3.9 mg, 0.012 mmol) and anhydrous 1,2-DCE (1.0 mL). The reaction mixture was heated at 65 °C for 10 min.

Radiosynthesis data are summarized below (**Table s8**).

Table s8: Radiosynthesis of **[^{18}F]4**

Starting activity	AY	Radiosynthesis time	RCP	A_m
20 GBq	156 MBq	78 min	> 99%	4. 99 GBq/µmol (d.c. EOS)

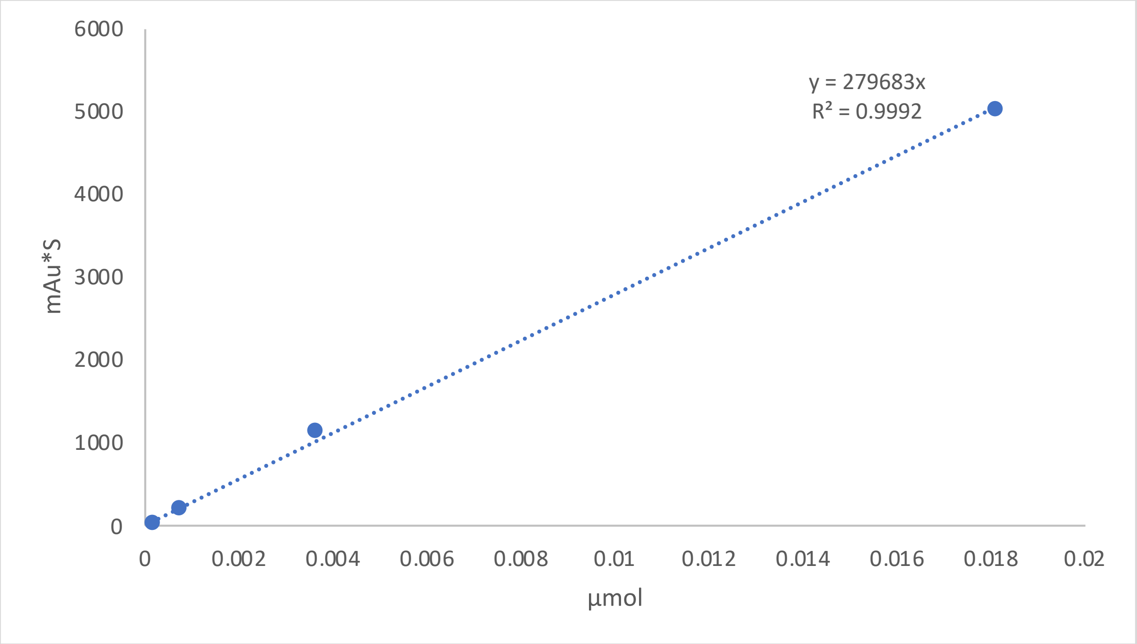

Figure s6: HPLC calibration curve for authentic reference *tert*-butyl 4,4-difluoropiperidine-1-carboxylate (**4**)

Table s9: Molar activity calculation of **[^{18}F]4**

Measurement	Activity injected (MBq, d.c.)	Peak area (mAu*s)	4 injected (µmol)	A_m (GBq/µmol)
1	0.4536	38.0	0.00013587	3.34
2	0.9348	34.2	0.00012228	7.65
3	0.5370	37.7	0.0001348	3.98

Average A_m: 4.99 ± 1.89 GBq/µmol (d.c. EOS)

ICP-MS

Following the automated synthesis and semi-preparative HPLC purification of [^{18}F]**4**, the full sample was allowed to decay and then analyzed by ICP-MS to determine the residual manganese content. This was done by comparison to a standard curve generated by a series of dilutions of known manganese concentration (**Figure s7**). From this method the quantity of ^{55}Mn was determined to be 4.07 µg/L, which is well below the threshold recommended by ICH guidelines for human injection.[9]

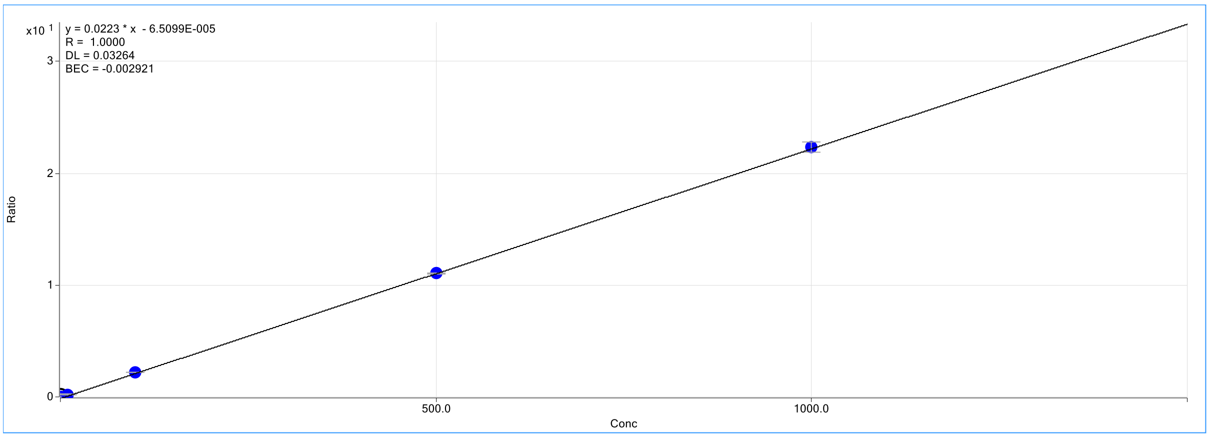

Figure s7: ICP-MS calibration curve for ^{55}Mn.

Synthesis of [^{18}F]4,4-difluoropiperidine ([^{18}F]18)

Following the automated radiosynthesis of [^{18}F]**4**, an aliquot of the product in MeCN (approximately 200 MBq) was diluted with 1 mL of a 4 M solution of HCl in 1,4-dioxane and the resulting mixture was heated at 60 °C for 15 min. An aliquot of the reaction mixture containing [^{18}F]4,4-difluoropiperidine hydrochloride ([^{18}F]**17**) was then filtered and analyzed by radioHPLC (conditions C). The radiochemical yield (RCY) of [^{18}F]**17** was determined by integration of the ^{18}F-product relative to the total peak area for all radioactive species observed. The reaction mixture was then diluted with 6 mL of a 1 M aqueous K_2CO_3 solution and loaded onto a Waters Oasis HLB cartridge, preconditioned with 10 mL of water (trapping efficiency = 34%). The cartridge was subsequently washed with 2 mL of water and purged with 10 mL of air, prior to the elution of [^{18}F]4,4-difluoropiperidine ([^{18}F]**18**) with 2 mL of MeCN (elution efficiency = 64%). An aliquot of the eluted product was filtered and analysed by radioHPLC (conditions C).

[^{18}F]4,4-Difluoropiperidine hydrochloride ([^{18}F]17)

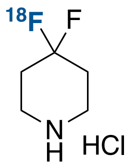

Entry	RCY (%)
**1**	> 99
**2**	> 99
**3**	> 99

Average RCY: > 99% (n = 3)

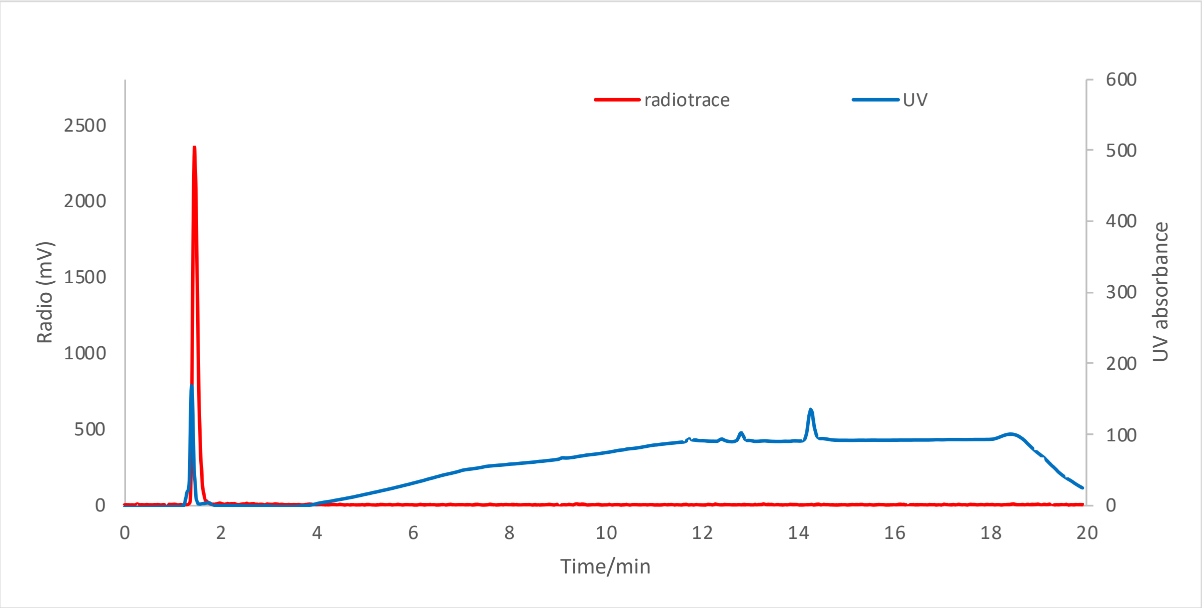

Note: for this run, the wavelength was adjusted to 190 nm.

HPLC conditions C

Post-labeling reactions

[18F]6-(4,4-difluoropiperidin-1-yl)-9-ethyl-9*H*-purine ([18F]19)

An oven-dried 3 mL v-vial equipped with a magnetic stirrer bar and capped with a septum was charged with 6-chloro-9-ethyl-9*H*-purine (9 mg, 0.05 mmol) and K_2CO_3 (7 mg, 0.05 mmol) in DMSO (0.5 mL). [^{18}F]**18** (approximately 5 MBq) in MeCN (approximately 100 µL) was subsequently added to the vial and the reaction mixture was stirred at 110 °C for 20 min. The reaction mixture was then diluted with MeCN (0.5 mL) and an aliquot was filtered and analysed by radioHPLC. The radiochemical yield (RCY) was determined by integration of the ^{18}F-product relative to the total peak area for all radioactive species observed.

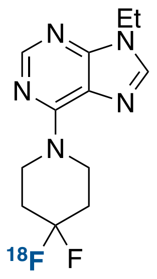

Entry	RCY (%)
1	85
2	98

Average RCY: 92 ± 7% (*n* = 2)

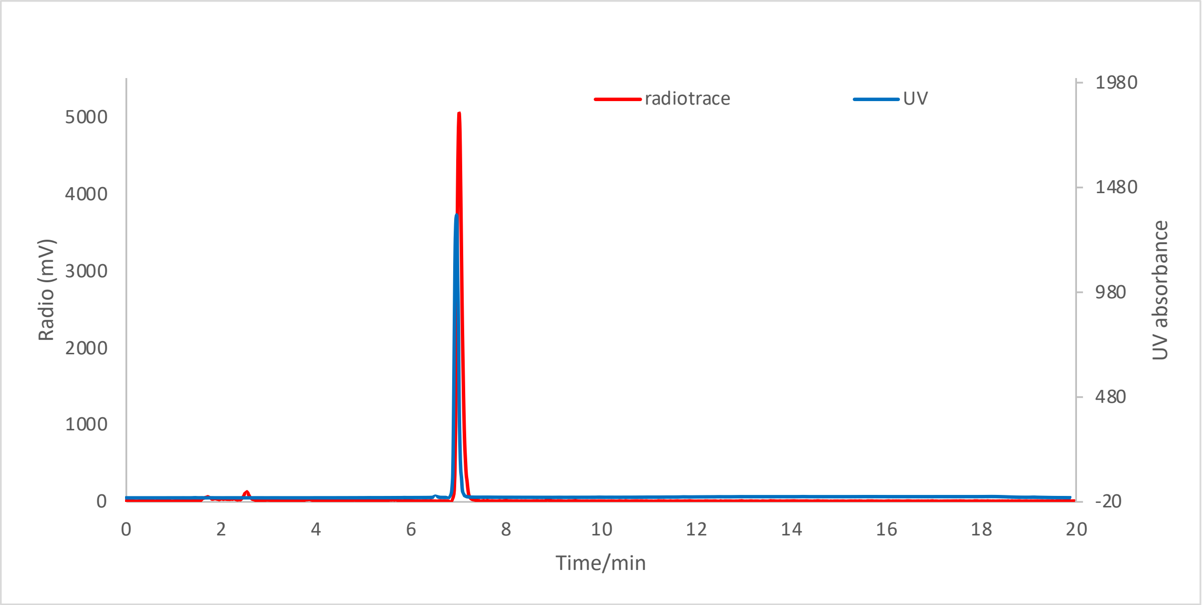

HPLC conditions C

[^{18}F](*E*)-3-(benzo[*d*][1,3]dioxol-5-yl)-1-(4,4-difluoropiperidin-1-yl)prop-2-en-1-one ([^{18}F]20)

An oven-dried 3 mL v-vial equipped with a magnetic stirrer and capped with a septum was charged with 2,5-dioxopyrrolidin-1-yl (*E*)-3-(benzo[*d*][1,3]dioxol-5-yl)acrylate (15 mg, 0.05 mmol) and K_2CO_3 (7 mg, 0.05 mmol) in DMA (0.5 mL). [^{18}F]**18** (approximately 5 MBq) in MeCN (approximately 100 µL) was subsequently added to the vial and the reaction mixture was stirred at 40 °C for 20 min. The reaction mixture was then diluted with MeCN (0.5 mL) and an aliquot was filtered and analysed by radioHPLC. The radiochemical yield (RCY) was determined by integration of the ^{18}F-product relative to the total peak area for all radioactive species observed.

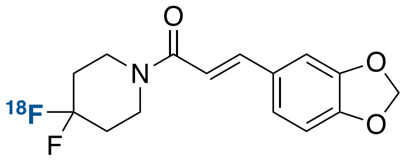

Entry	RCY (%)
1	65
2	64

Average RCY: 65 ± 1% (*n* = 2)

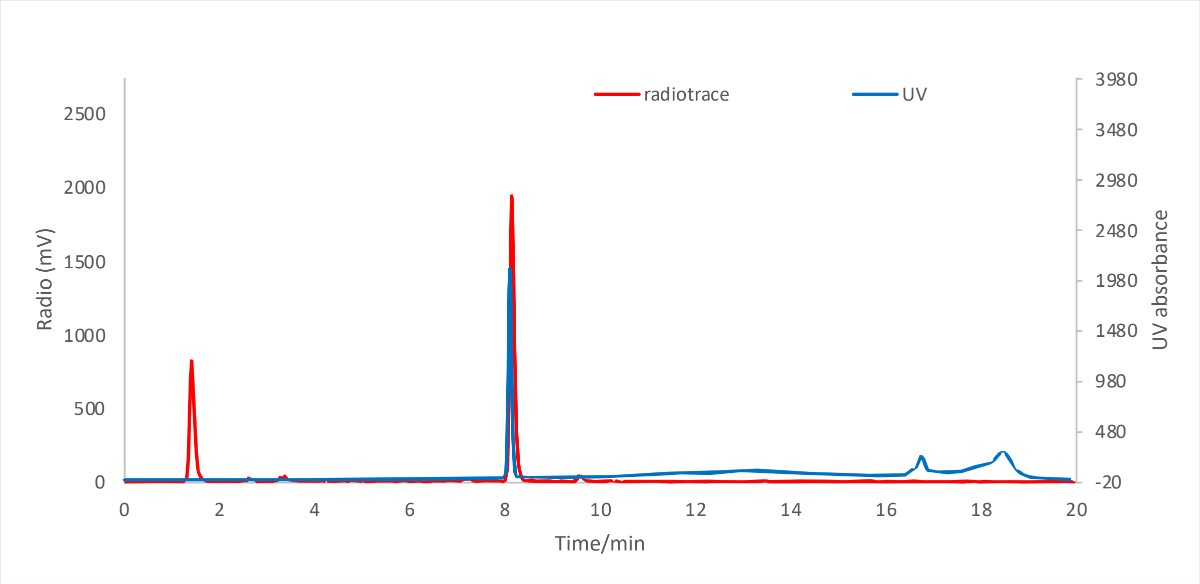

HPLC conditions C

[^{18}F]2-(5-((4,4-difluoropiperidin-1-yl)sulfonyl)-2-ethoxyphenyl)-5-methyl-7-propyl-3,4a,5,7a-tetrahydro-4*H*-pyrrolo[3,2-*d*]pyrimidin-4-one ([^{18}F]21)

An oven-dried 3 mL v-vial equipped with a magnetic stirrer bar and capped with a septum was charged with 4-ethoxy-3-(1-methyl-7-oxo-3-propyl-6,7-dihydro-1*H*-pyrazolo[4,3-*d*]pyrimidin-5-yl)benzene-1-sulfonyl chloride (21 mg, 0.05 mmol) and NEt_3 (14 µL, 0.1 mmol) in THF (0.5 mL). [^{18}F]**18** (approximately 5 MBq) in MeCN (approximately 100 µL) was subsequently added to the vial the reaction mixture was stirred at 40 °C for 20 min. The reaction mixture was then diluted with MeCN (0.5 mL) and an aliquot was filtered and analysed by radioHPLC. The radiochemical yield (RCY) was determined by integration of the ^{18}F-product relative to the total peak area for all radioactive species observed.

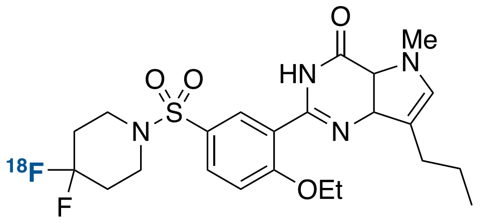

Entry	RCY (%)
**1**	90
**2**	93

Average RCY: 92 ± 2% (n = 2)

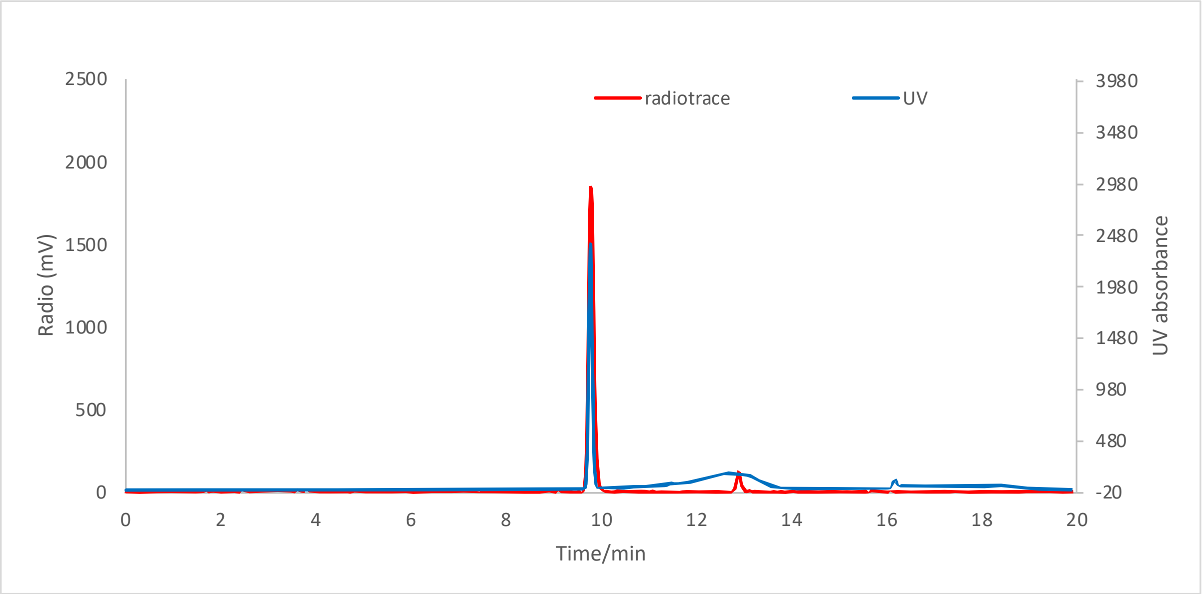

HPLC conditions C

Unsuccessful substrates

Selected substrates were found to be unsuccessful in our ^{18}F-fluorodecarboxylation protocol. These are shown below (**Figure s8**).

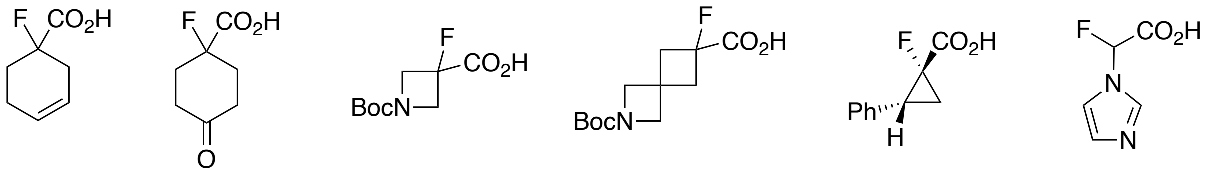

22a

Figure s8: Unsuccessful substrates.

References

1. Wang, Z.; Guo, C.-Y.; Yang, C.; Chen, J.-P. Ag-Catalyzed Chemoselective Decarboxylative Mono- and *gem*-Difluorination of Malonic Acid Derivatives. *J. Am. Chem. Soc*. **2019**, *141*, 5617–5622.
2. Melnykov, K. P.; Nazar, K.; Smyrnov, O.; Skreminskyi, A.; Pavlenko, S.; Klymenko-Ulianov, O.; Shishkina, S.; Volochnyuk, D. M.; Grygorenko, O. O. Mono- and Difluorinated Saturated Heterocyclic Amines for Drug Discovery: Systematic Study of Their Physicochemical Properties. *Chem. Eur. J.* **2023**, *29*, e202301383.
3. Zhao, H.; Lu, C.; Herbert, S.; Zhang, W.; Shen, Q. Difluoromethylation of Alkyl Bromides and Iodides with $TMSCF_2H$. *J. Org. Chem*. **2021**, *86*, 2854–2865.
4. Reddy, G. L.; Dar, M. I.; Abhinandan D. H.; Mahajan, P.; Nargotra, A.; Baba, A. M.; Nandi, U.; Wazir, P.; Singh, G.; Vishwakarmaa, R. A.; Syed, S. H.; Sanghapal, D. S. Design, synthesis and biological evaluation of pyrazolopyrimidinone based potent and selective PDE5 inhibitors for treatment of erectile dysfunction. *Bioorg. Chem*. **2019**, *89*, 103022.
5. Webb, E. W.; Park, J. B.; Cole, E. L.; Donnelly, D. J.; Bonacorsi, S. J.; Ewing, W. R.; Doyle, A. G. Nucleophilic (Radio)Fluorination of Redox-Active Esters via Radical-Polar Crossover Enabled by Photoredox Catalysis. *J. Am. Chem. Soc.* **2020**, *142*, 9493–9500.
6. Jiang, X.; Gandelman, M. Enantioselective Suzuki Cross-Couplings of Unactivated 1-Fluoro-1-haloalkanes: Synthesis of Chiral β-, γ-, δ-, and ε-Fluoroalkanes. *J. Am. Chem. Soc*. **2015**, *137*, 2542–2547.
7. Zeng, X.; Yan, W.; Zacate, S. B.; Cai, A.; Wang, Y.; Yang, D.; Yang, K.; Liu, W. Copper-Catalyzed Deaminative Difluoromethylation. *Angew. Chem. Int. Ed.* **2020**, *59*, 16398–16403.
8. International Council For Harmonisation Of Technical Requirements For Pharmaceuticals For Human Use, *Guideline On The Specification Limits For Residues Of Metal Catalysts Or Metal Reagents.* International Council for Harmonisation of Technical Requirements for Pharmaceuticals for Human Use, 2008. https://www.ema.europa.eu/en/documents/scientific-guideline/guideline-specification-limits-residues-metal-catalysts-or-metal-reagents_en.pdf (accessed 2024-09-25).

NMR spectra

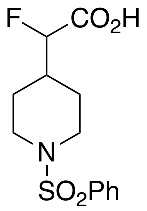

^{1}H NMR (400 MHz, DMSO-d_6)
7a

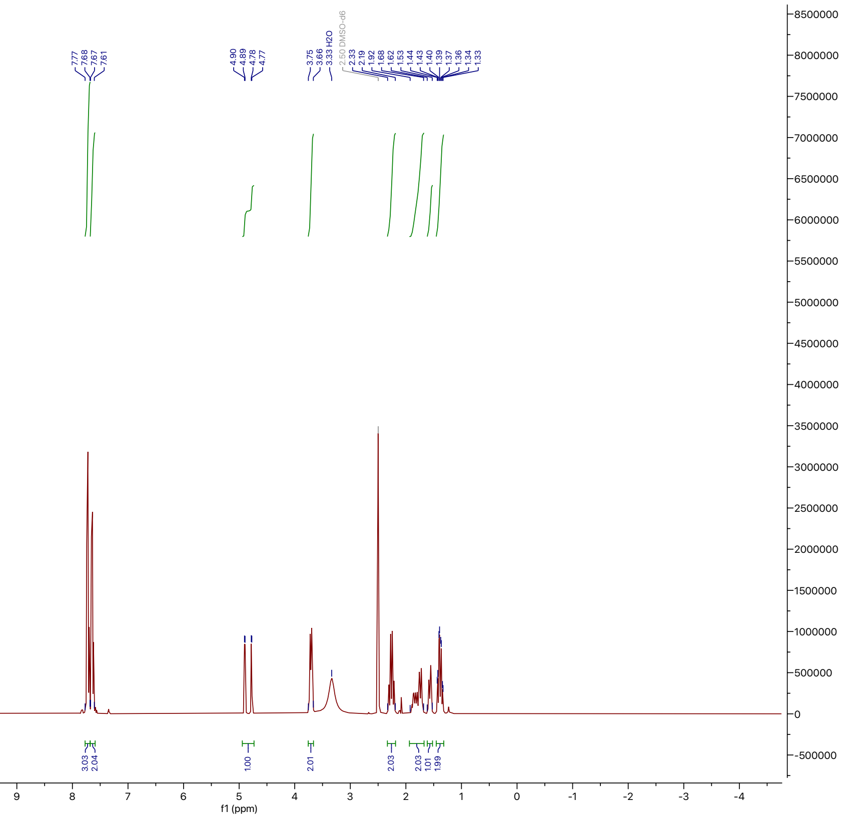

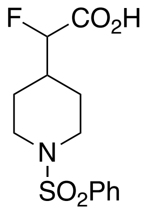

^{19}F NMR (377 MHz, DMSO-d_6)
7a

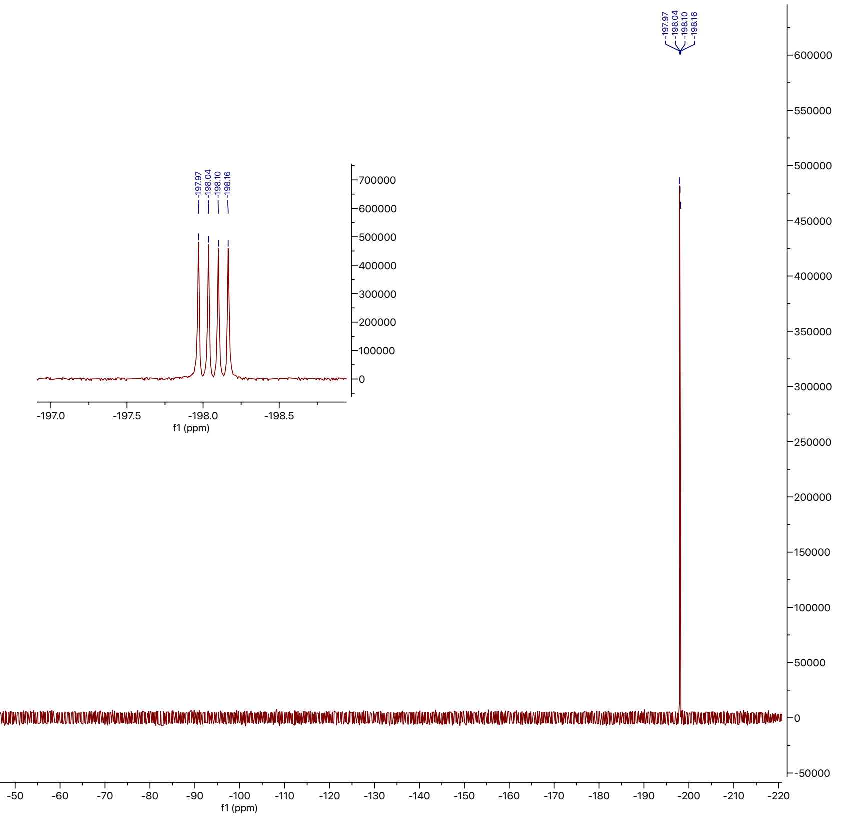

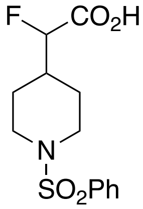

^{13}C NMR (101 MHz, DMSO-d_6)
7a

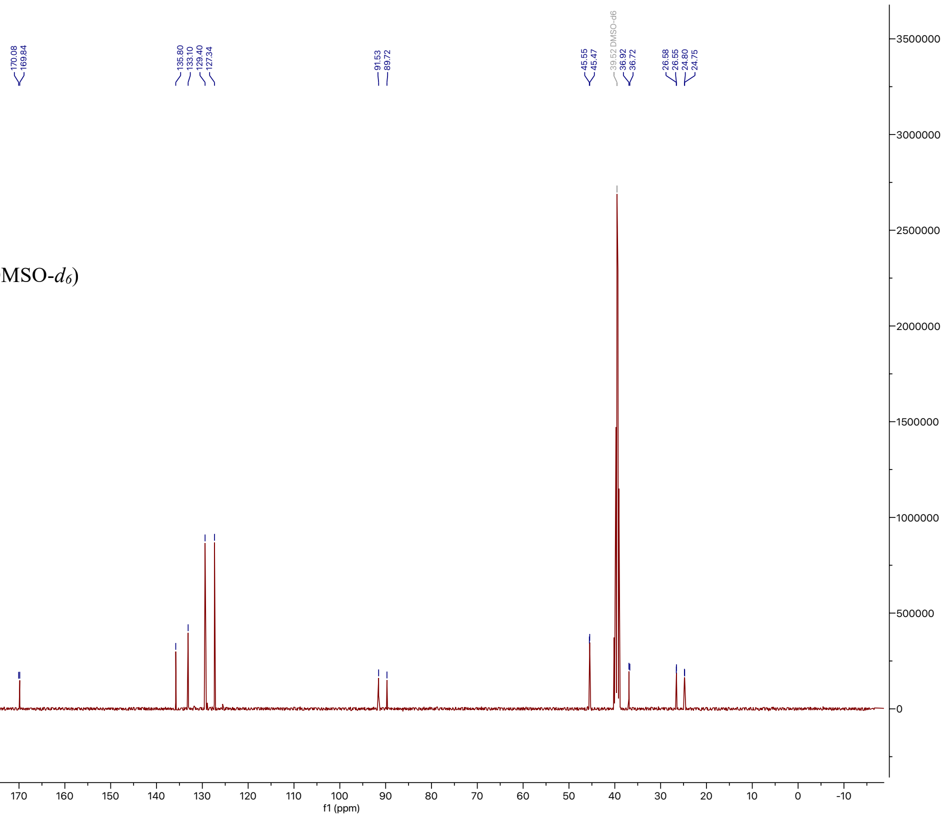

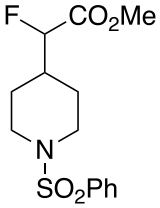

^1H NMR (400 MHz, $CDCl_3$)
7b

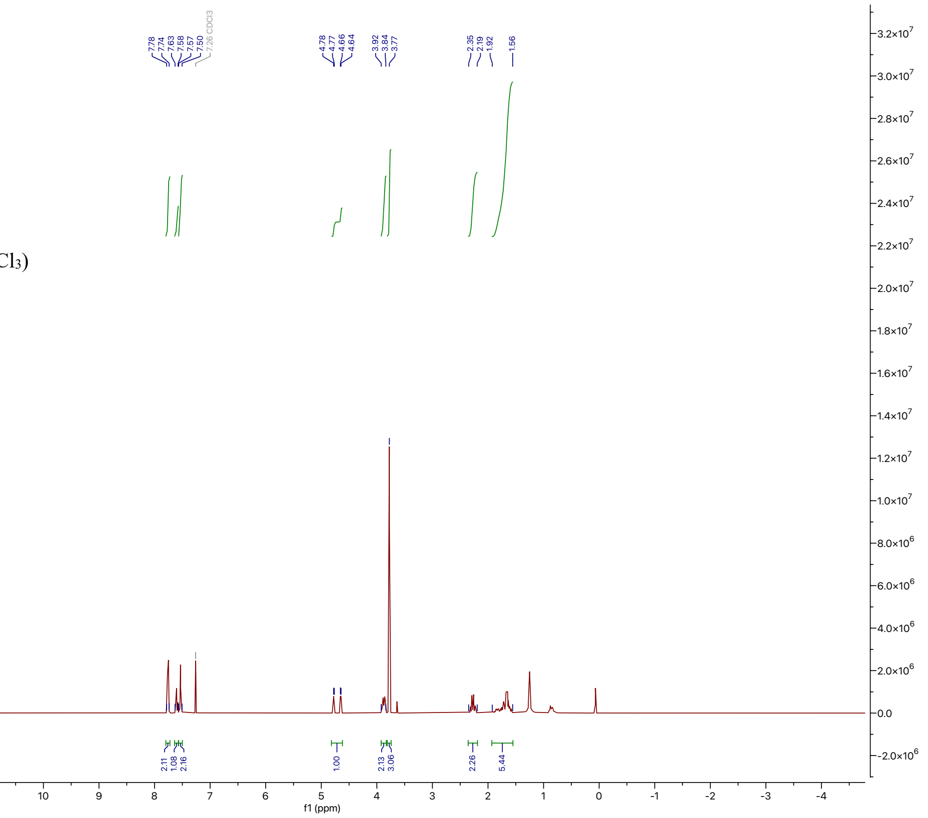

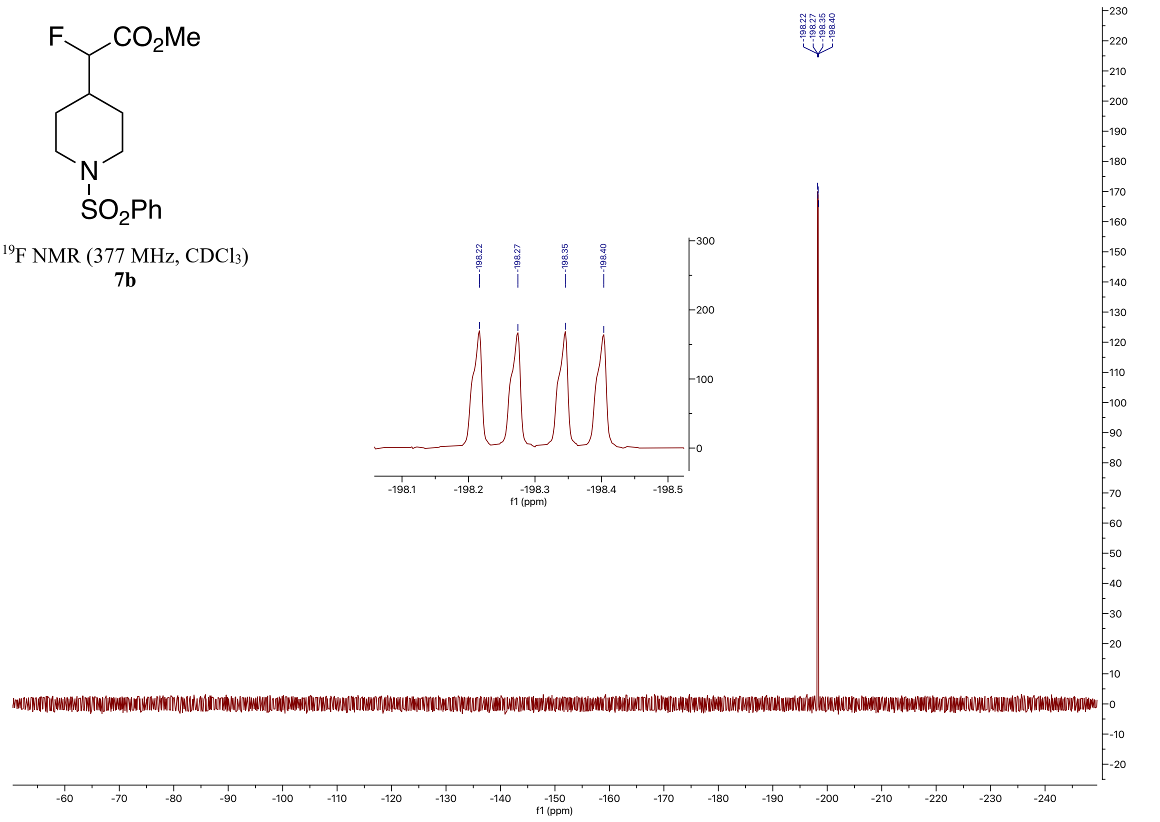

^{19}F NMR (377 MHz, $CDCl_3$)
7b

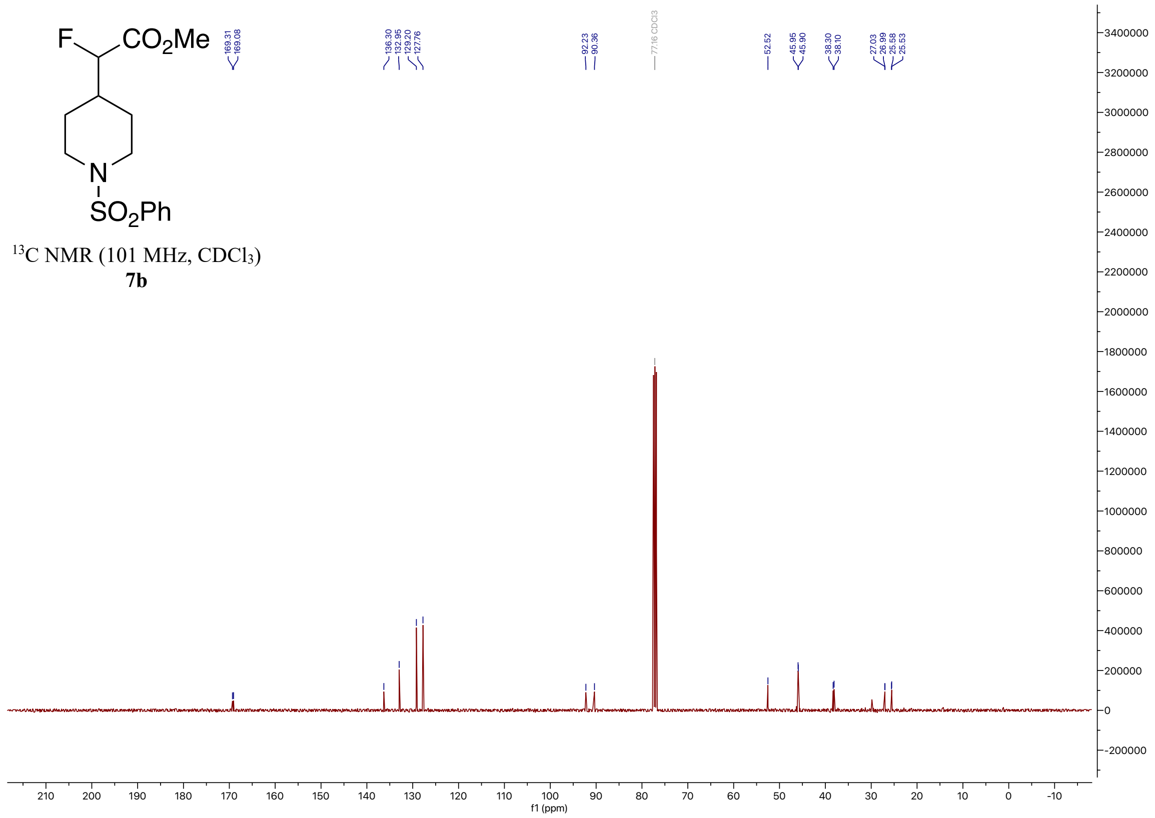

^{13}C NMR (101 MHz, $CDCl_3$)
7b

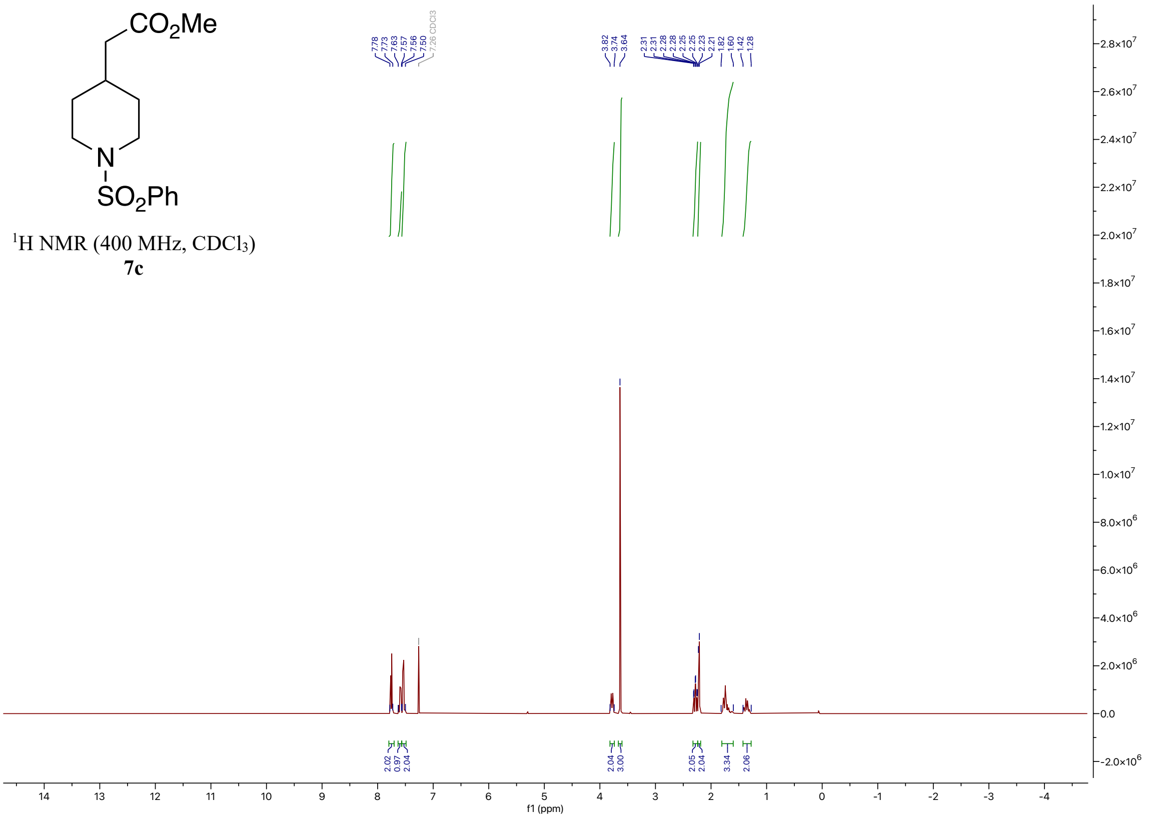

^{1}H NMR (400 MHz, $CDCl_3$)
7c

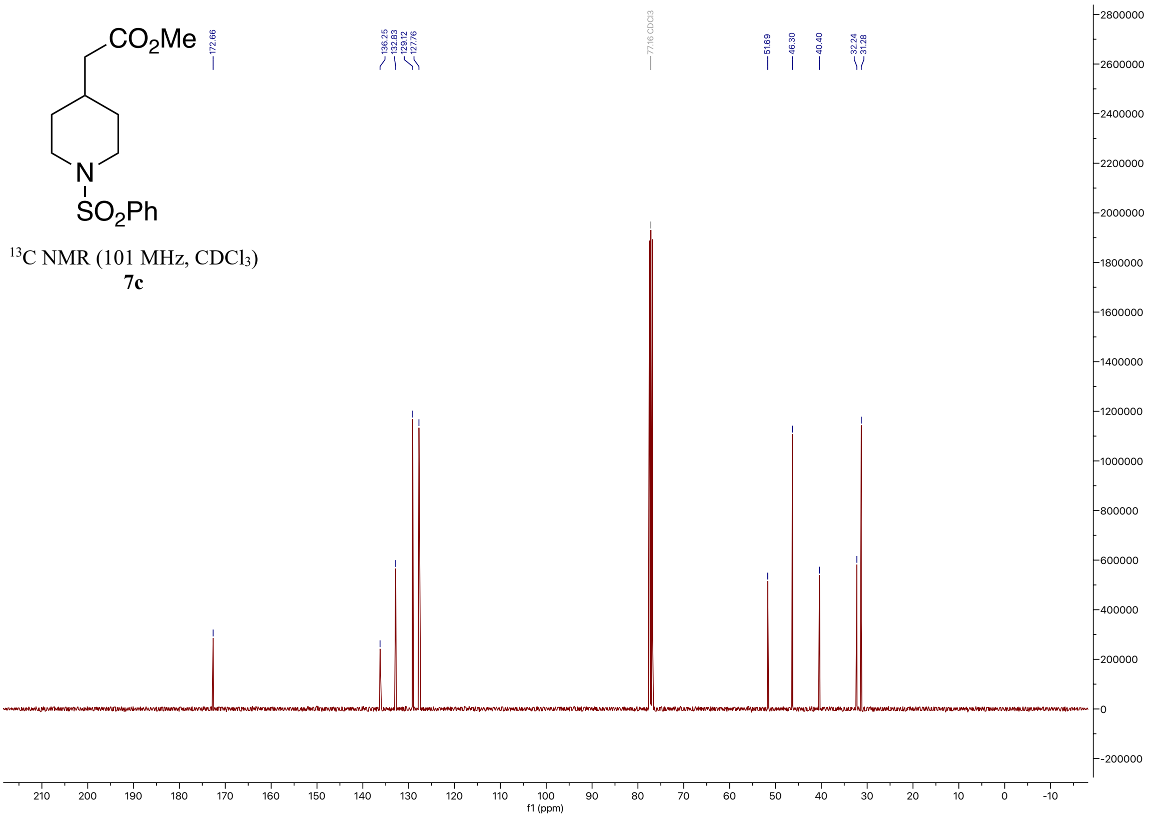

^{13}C NMR (101 MHz, $CDCl_3$)
7c

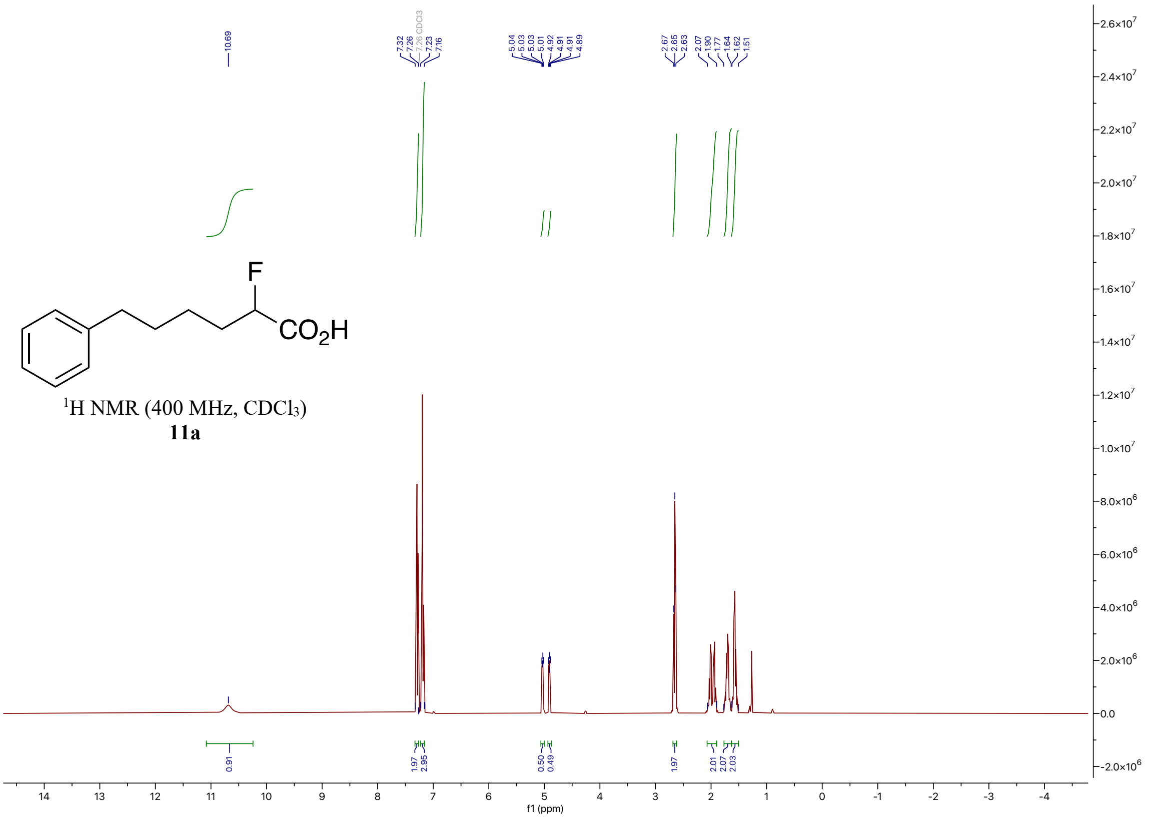

^{1}H NMR (400 MHz, $CDCl_3$)
11a

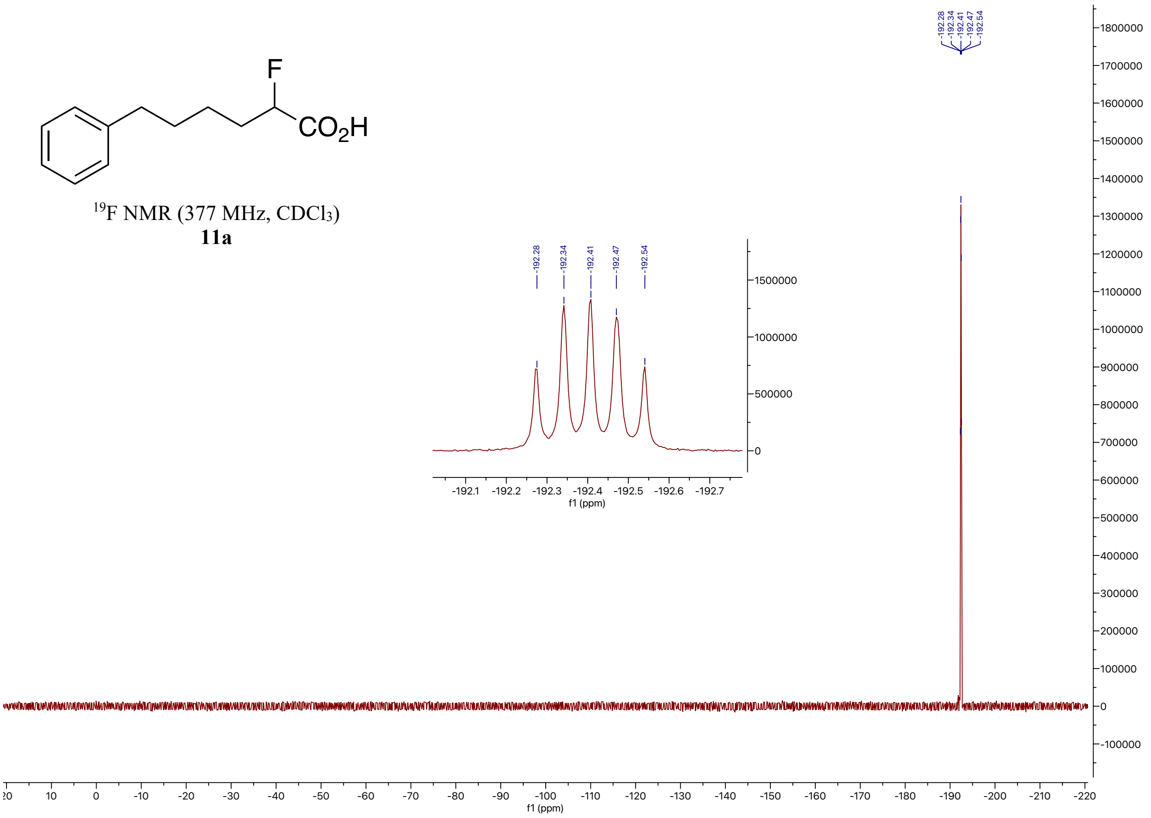

^{19}F NMR (377 MHz, $CDCl_3$)
11a

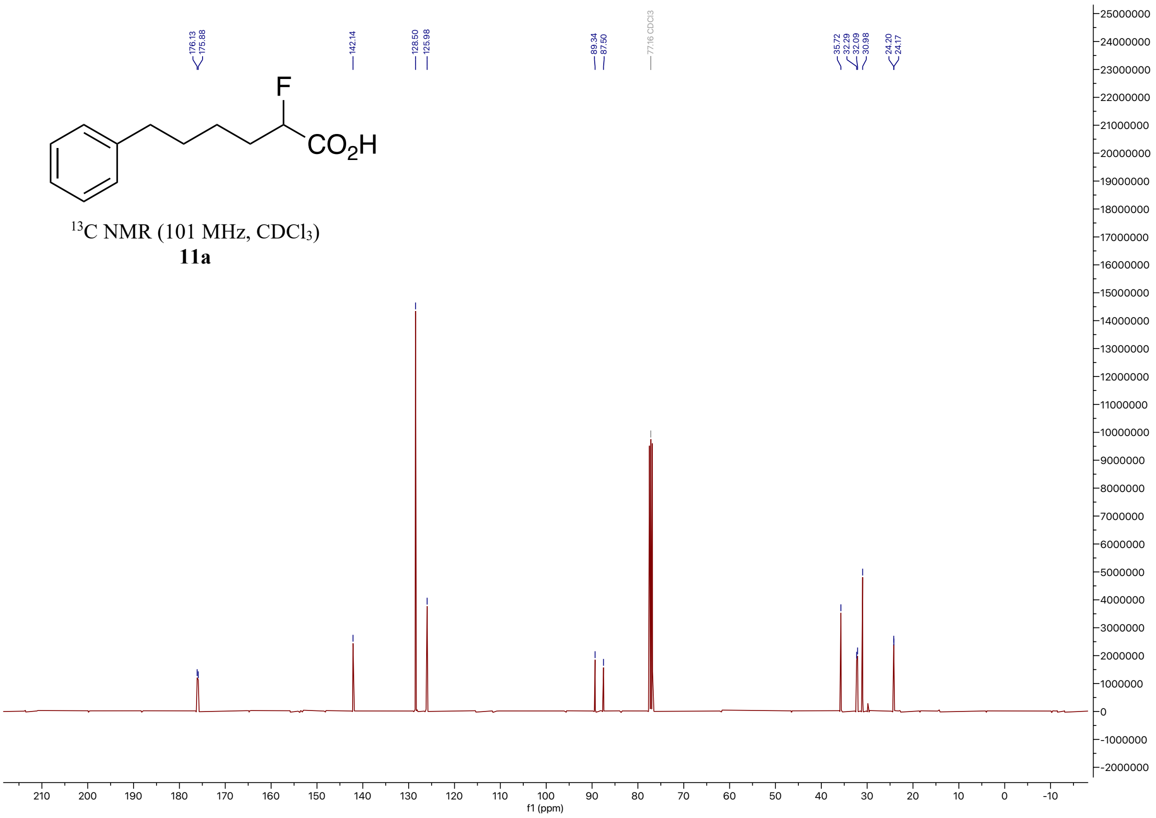

^{13}C NMR (101 MHz, $CDCl_3$)

11a

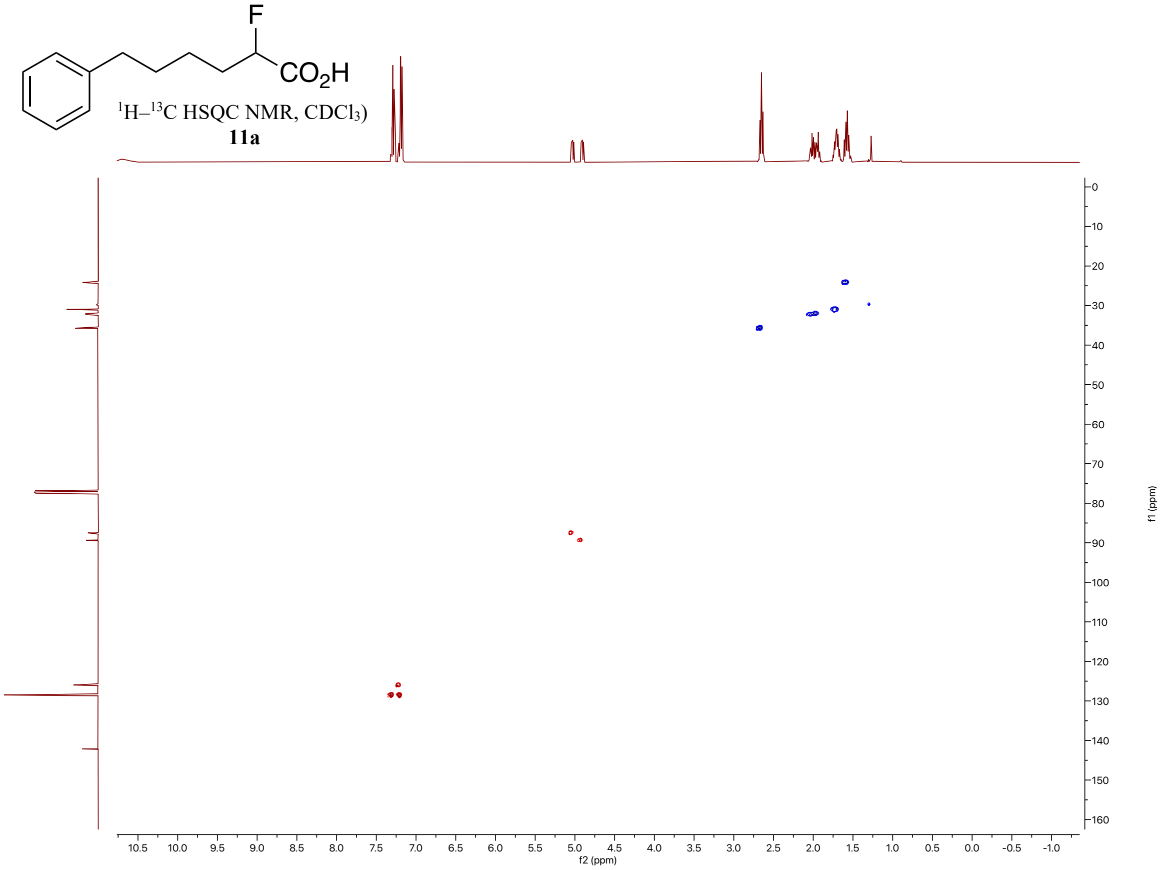

^{1}H–^{13}C HSQC NMR, $CDCl_3$)

11a

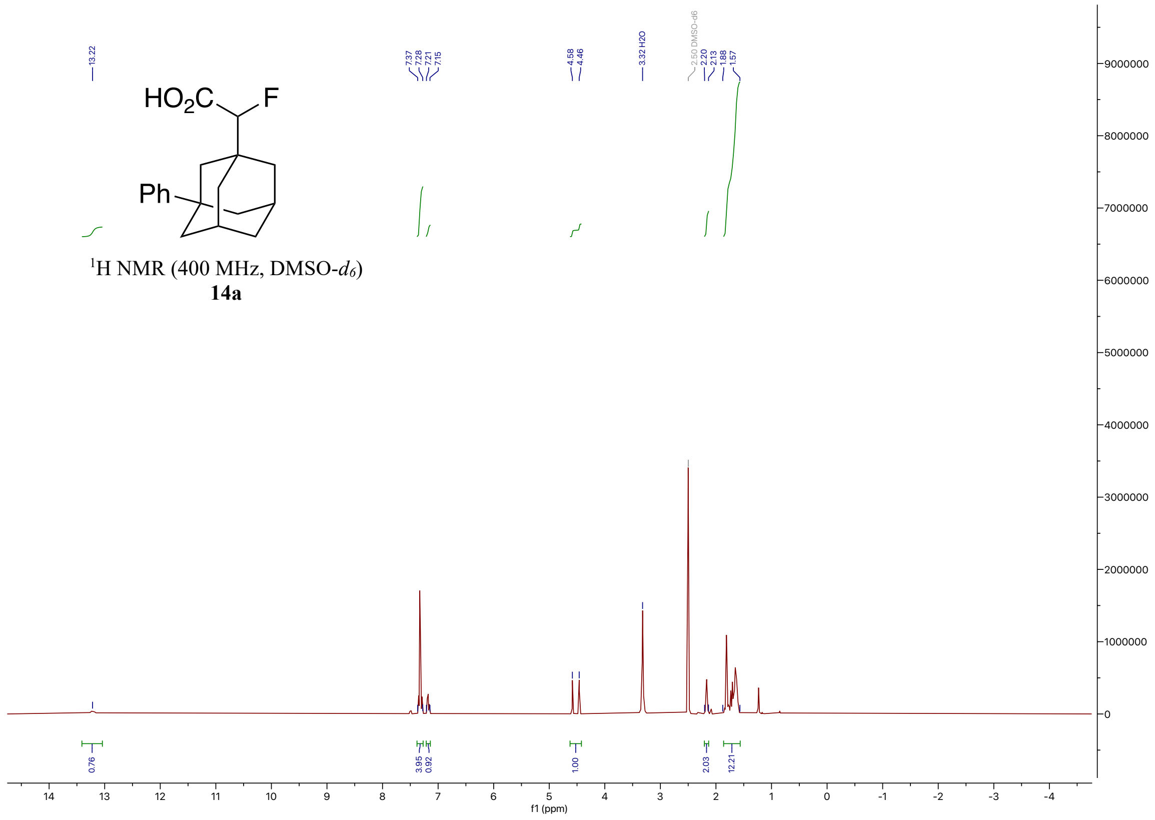

^{1}H NMR (400 MHz, DMSO-d_6)
14a

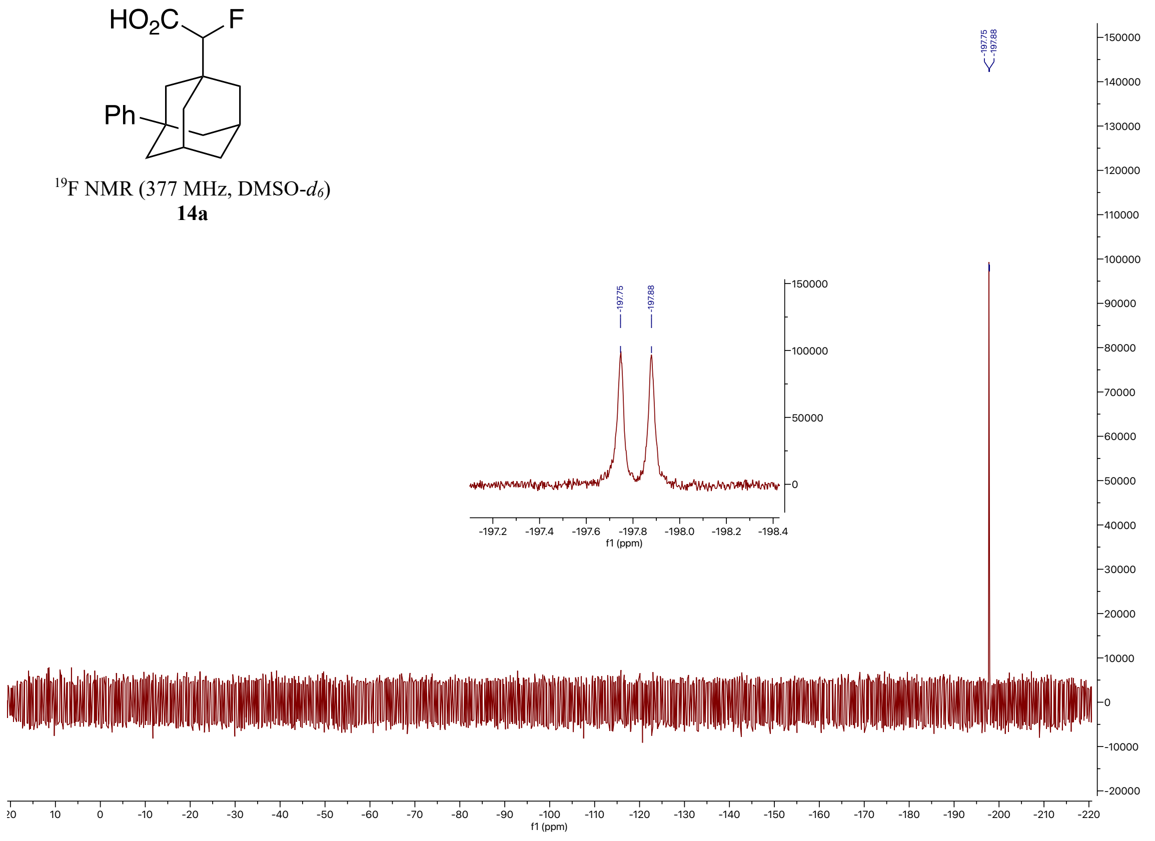

^{19}F NMR (377 MHz, DMSO-d_6)
14a

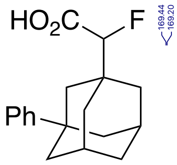

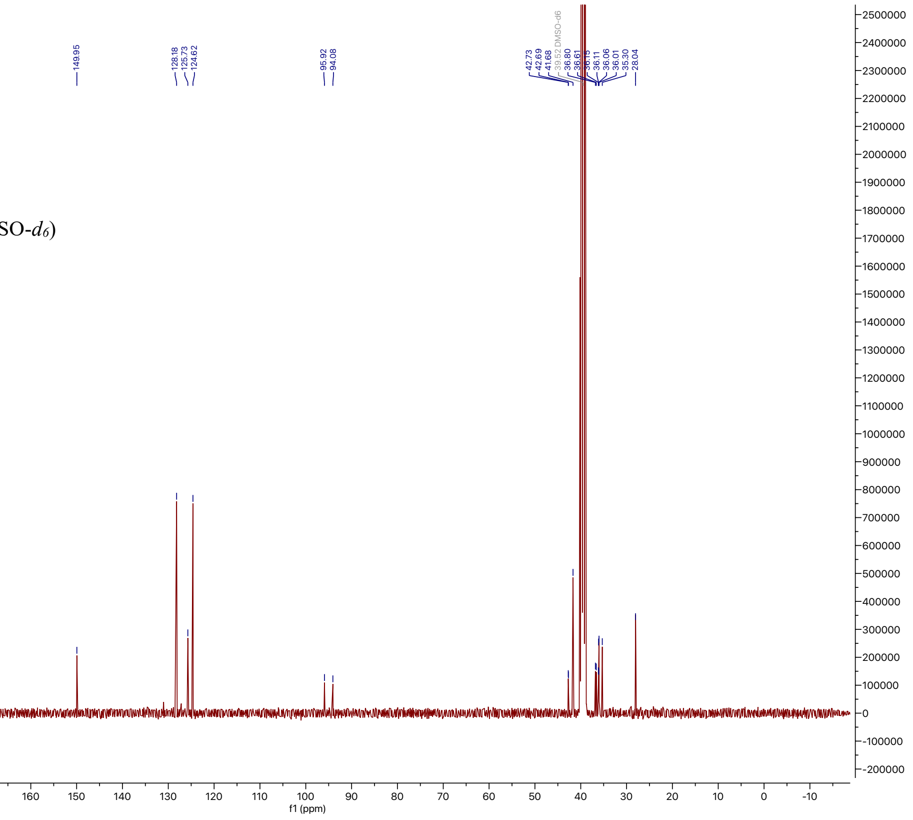

^{13}C NMR (101 MHz, DMSO-d_6)

14a

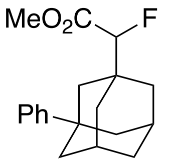

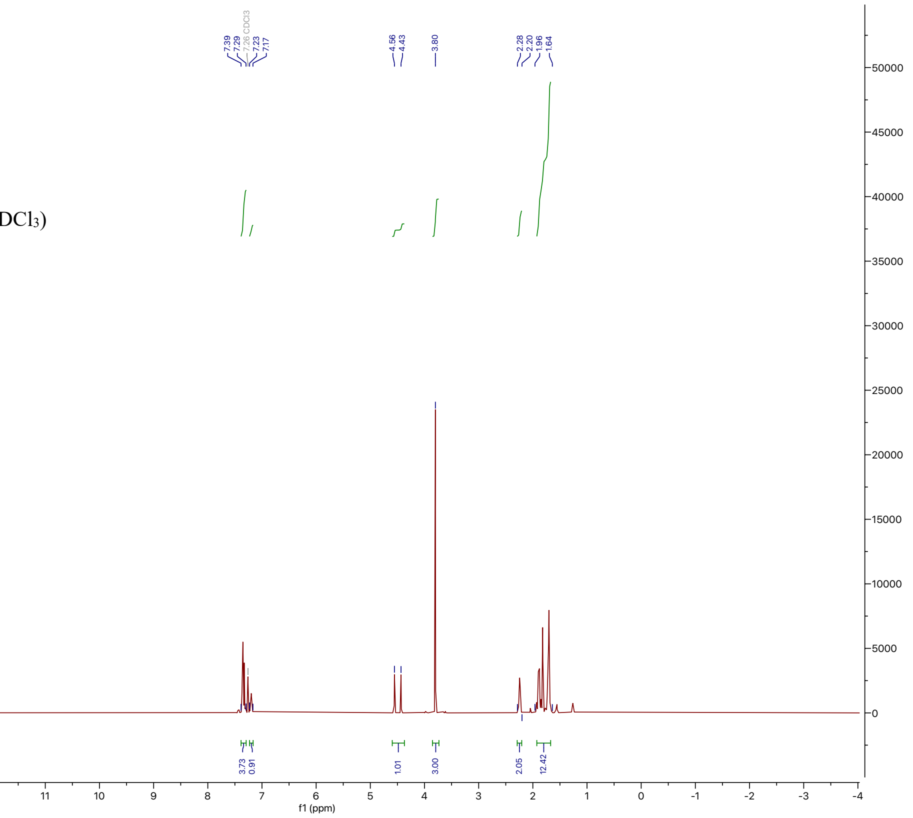

^{1}H NMR (400 MHz, $CDCl_3$)

14b

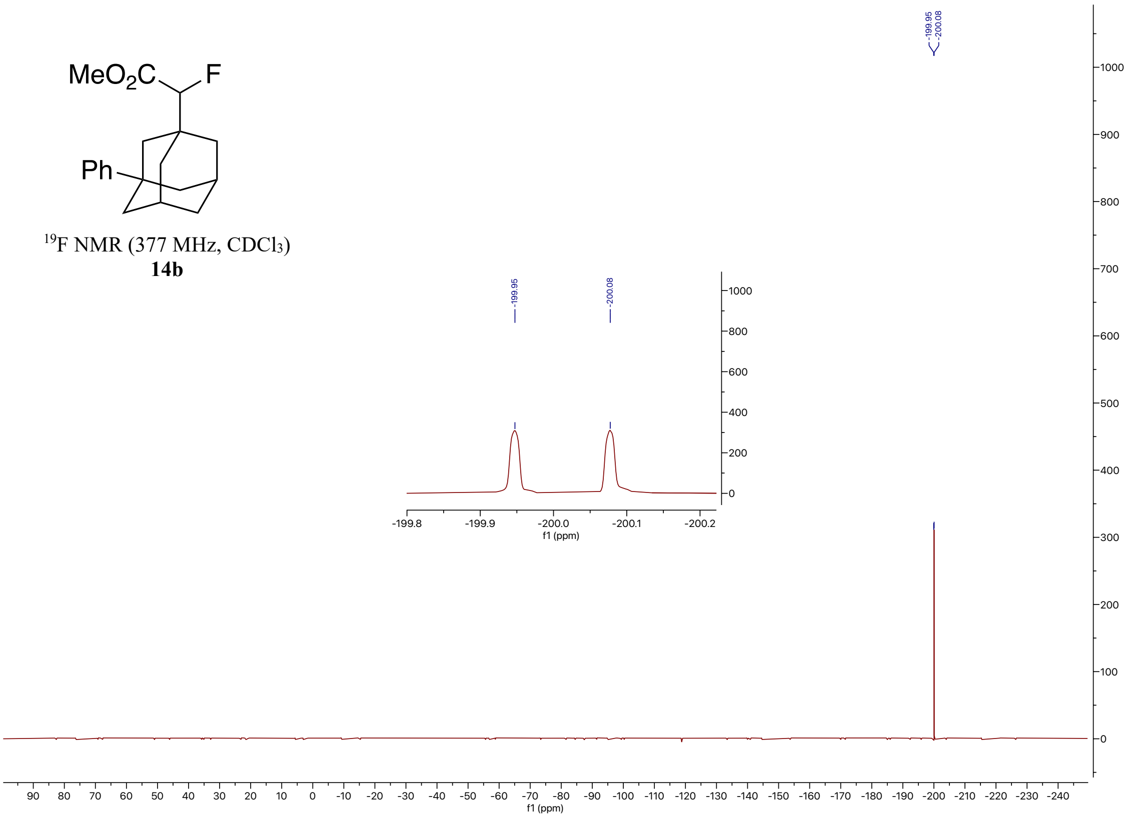

^{19}F NMR (377 MHz, $CDCl_3$)
14b

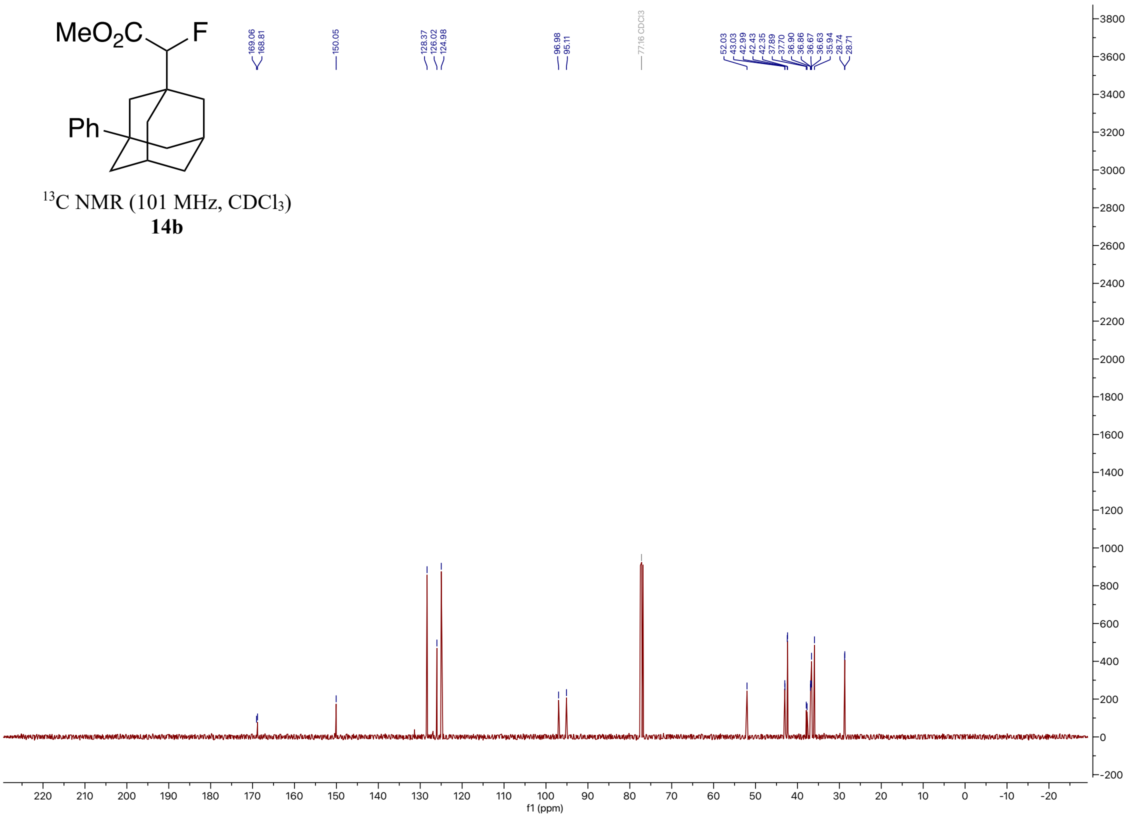

^{13}C NMR (101 MHz, $CDCl_3$)
14b

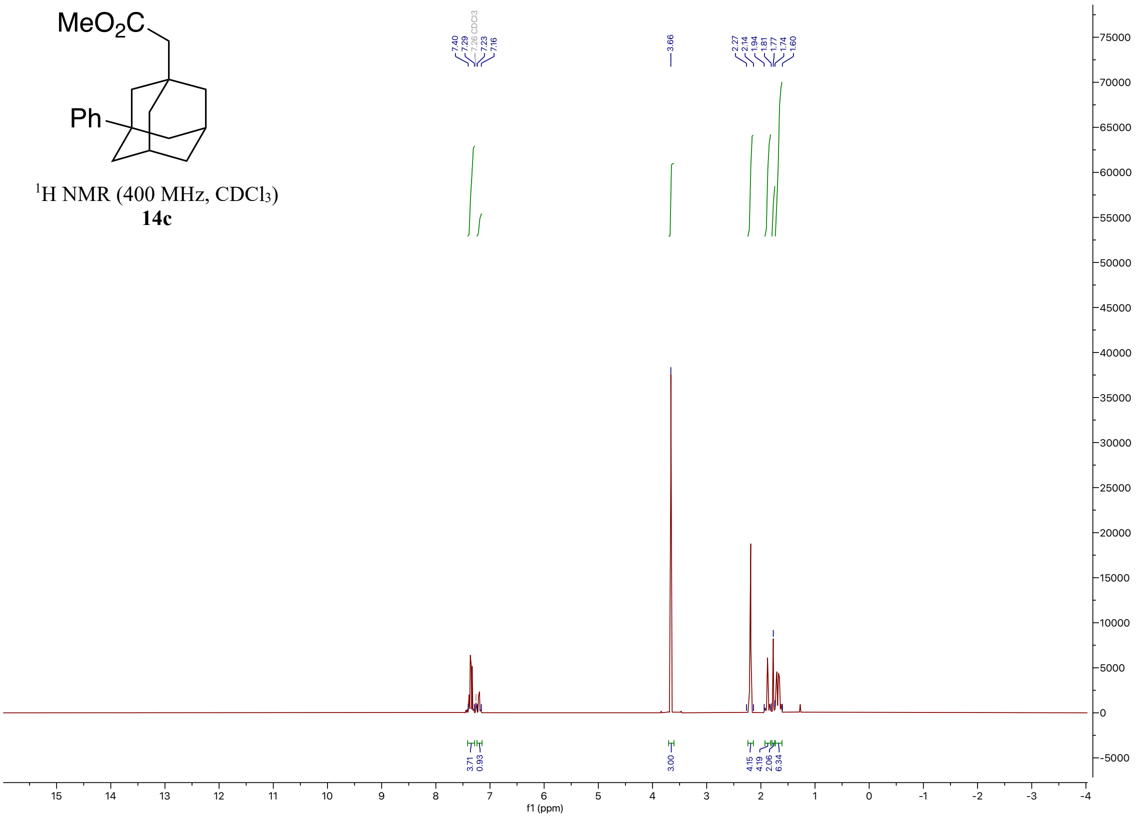

^{1}H NMR (400 MHz, $CDCl_3$)
14c

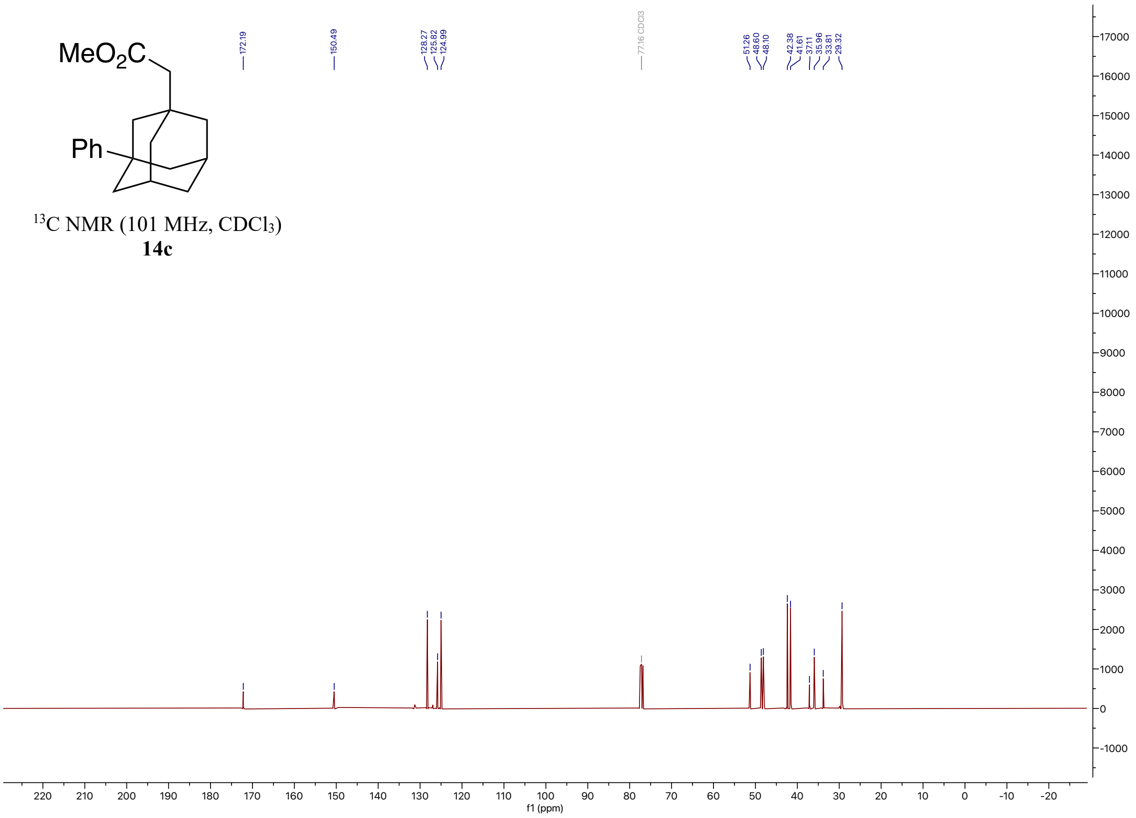

^{13}C NMR (101 MHz, $CDCl_3$)
14c

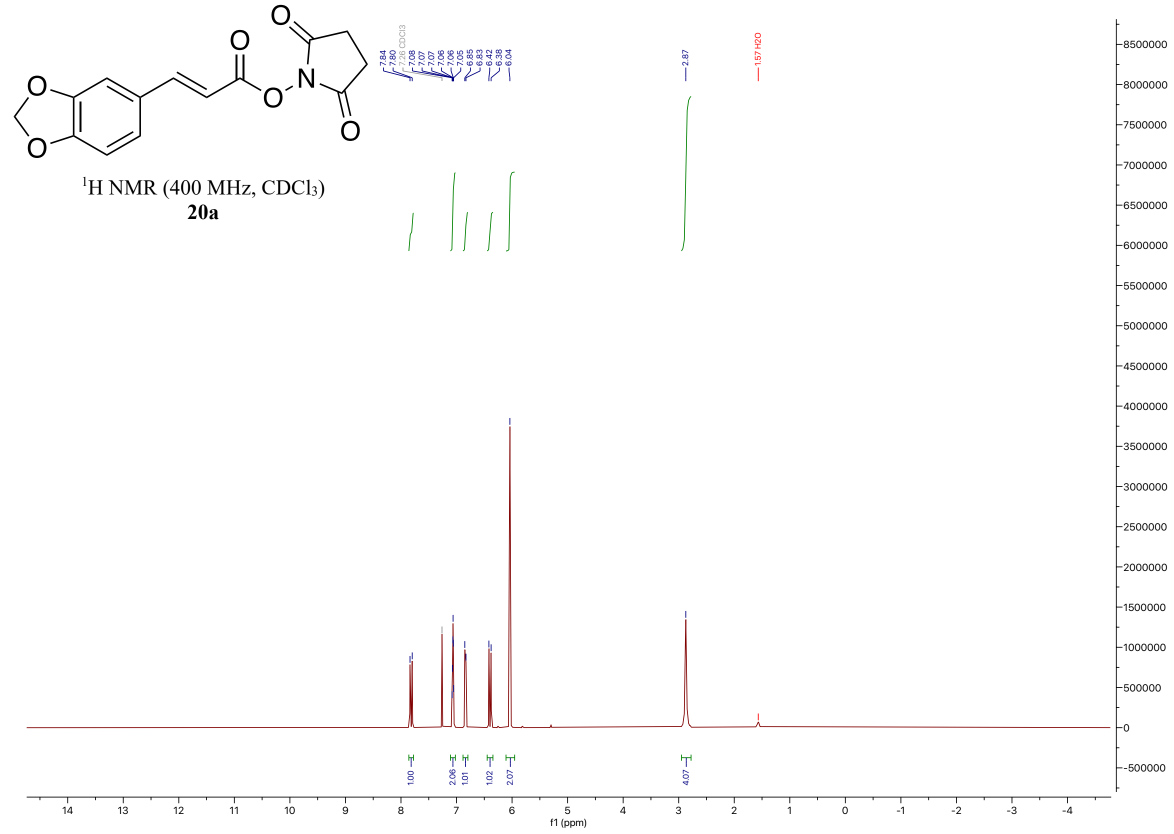

^{1}H NMR (400 MHz, $CDCl_3$)
20a

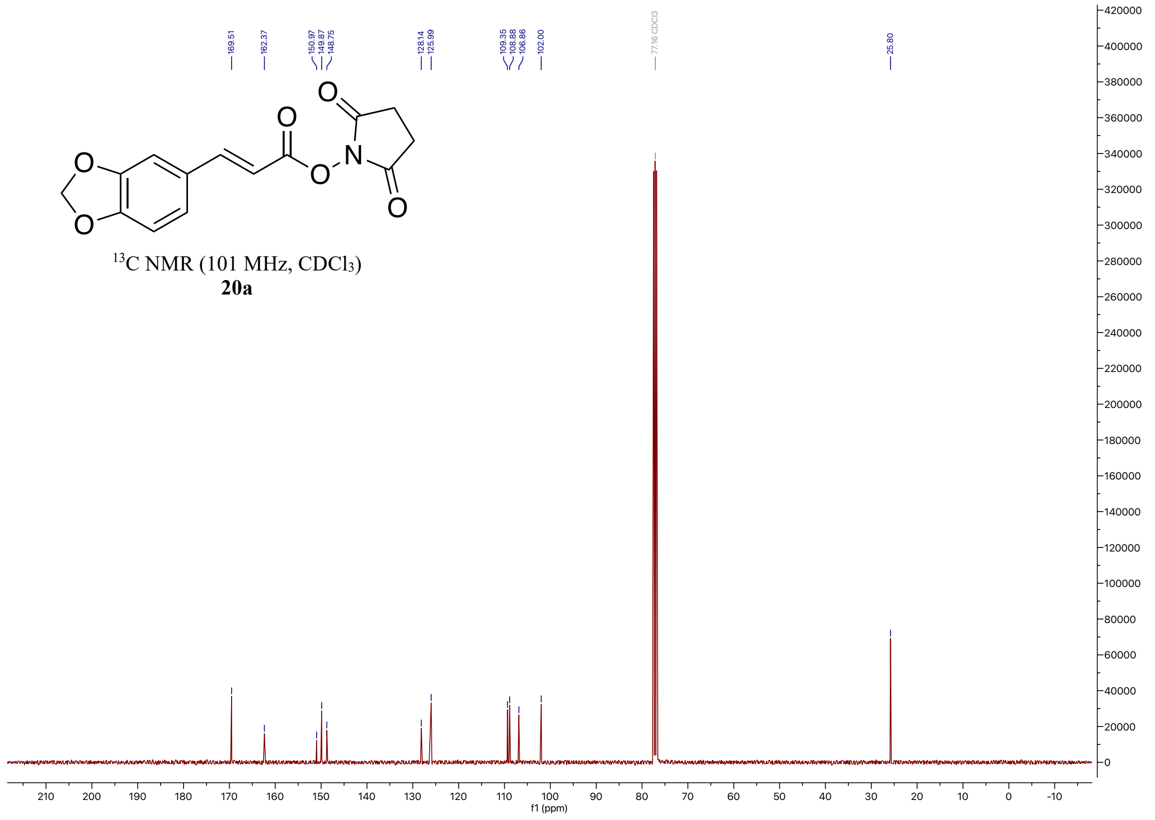

^{13}C NMR (101 MHz, $CDCl_3$)
20a

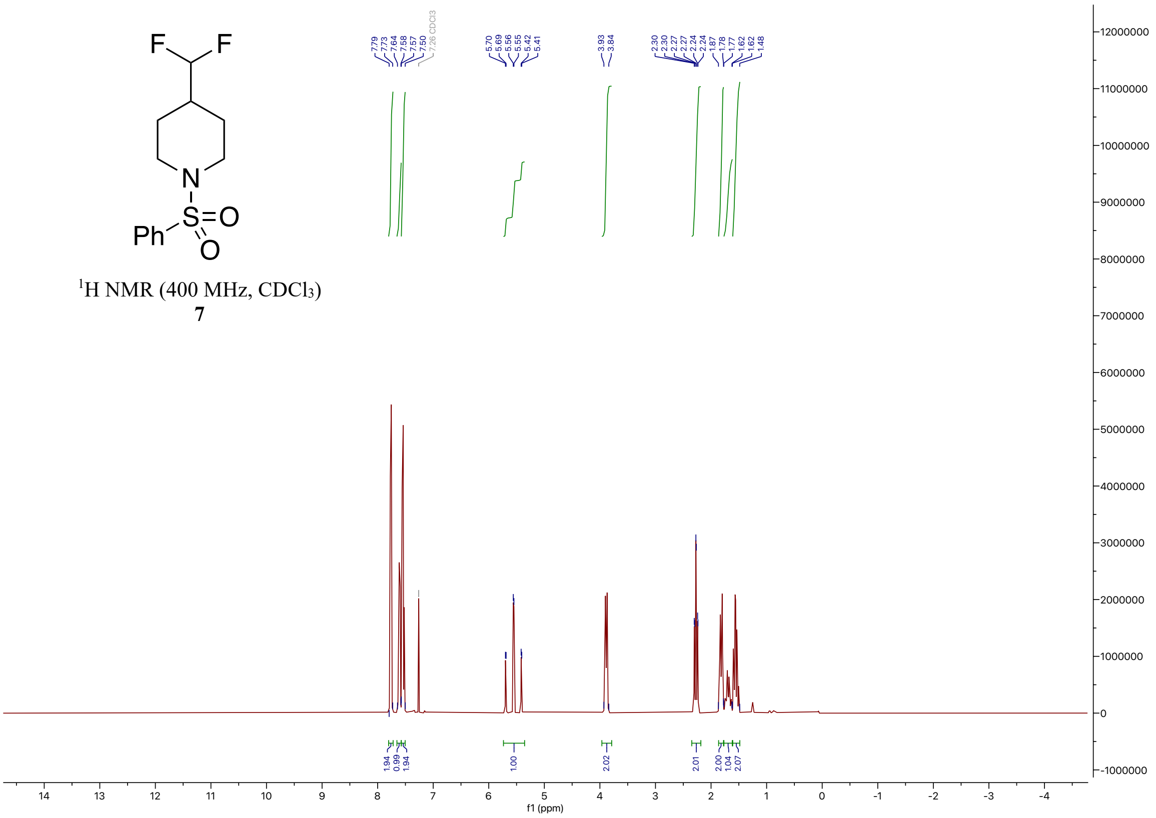

^{1}H NMR (400 MHz, $CDCl_3$)

7

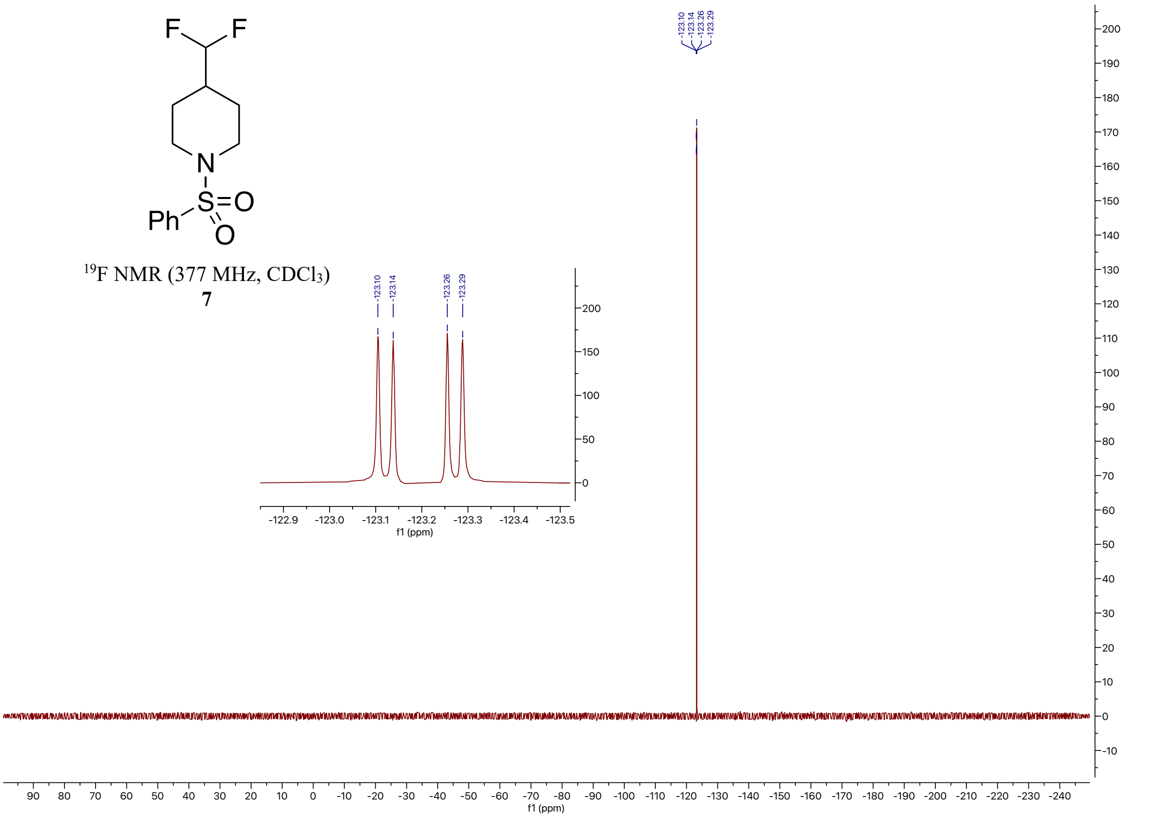

^{19}F NMR (377 MHz, $CDCl_3$)

7

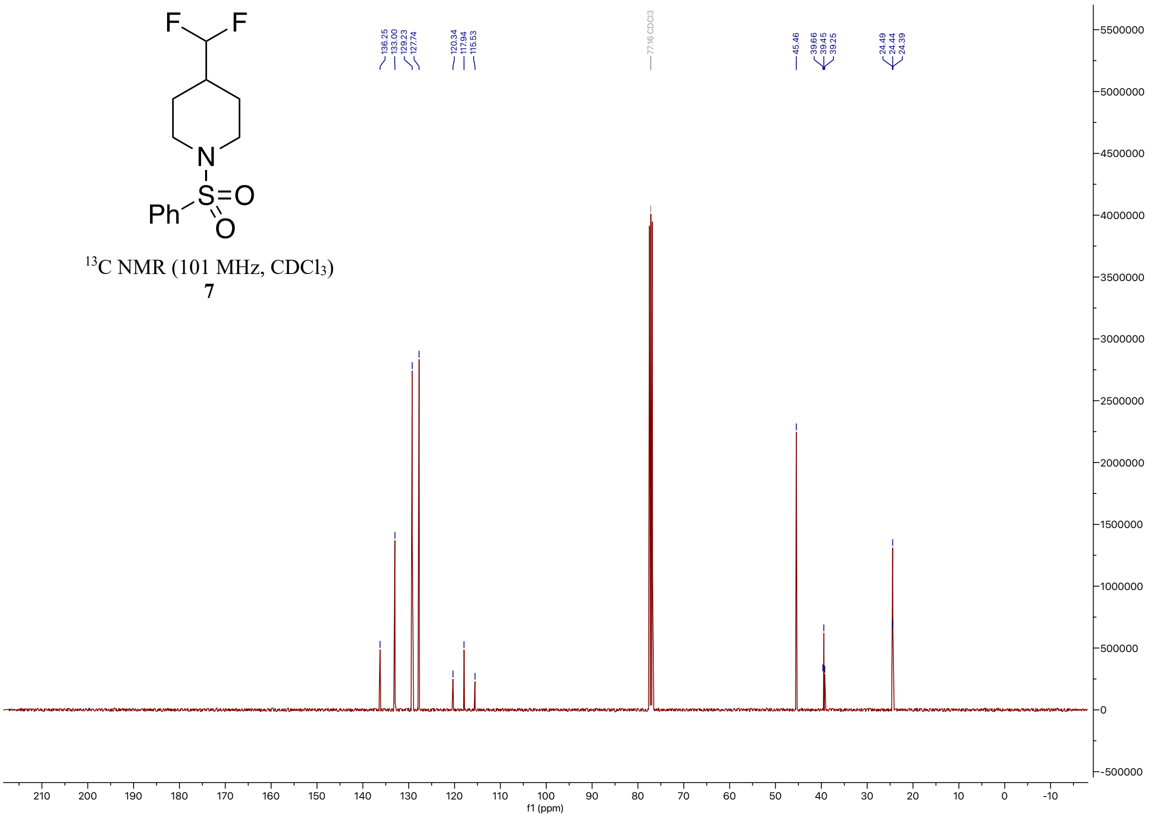

^{13}C NMR (101 MHz, $CDCl_3$)
7

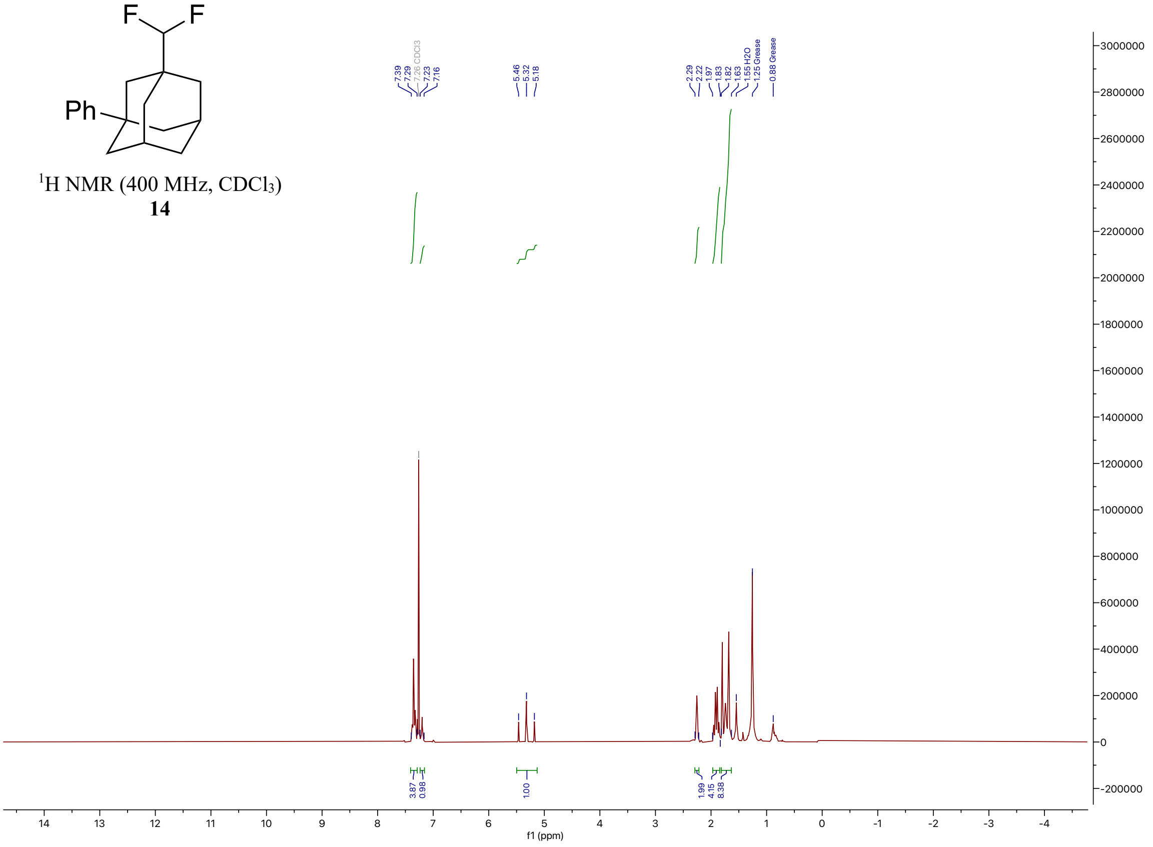

^{1}H NMR (400 MHz, $CDCl_3$)
14

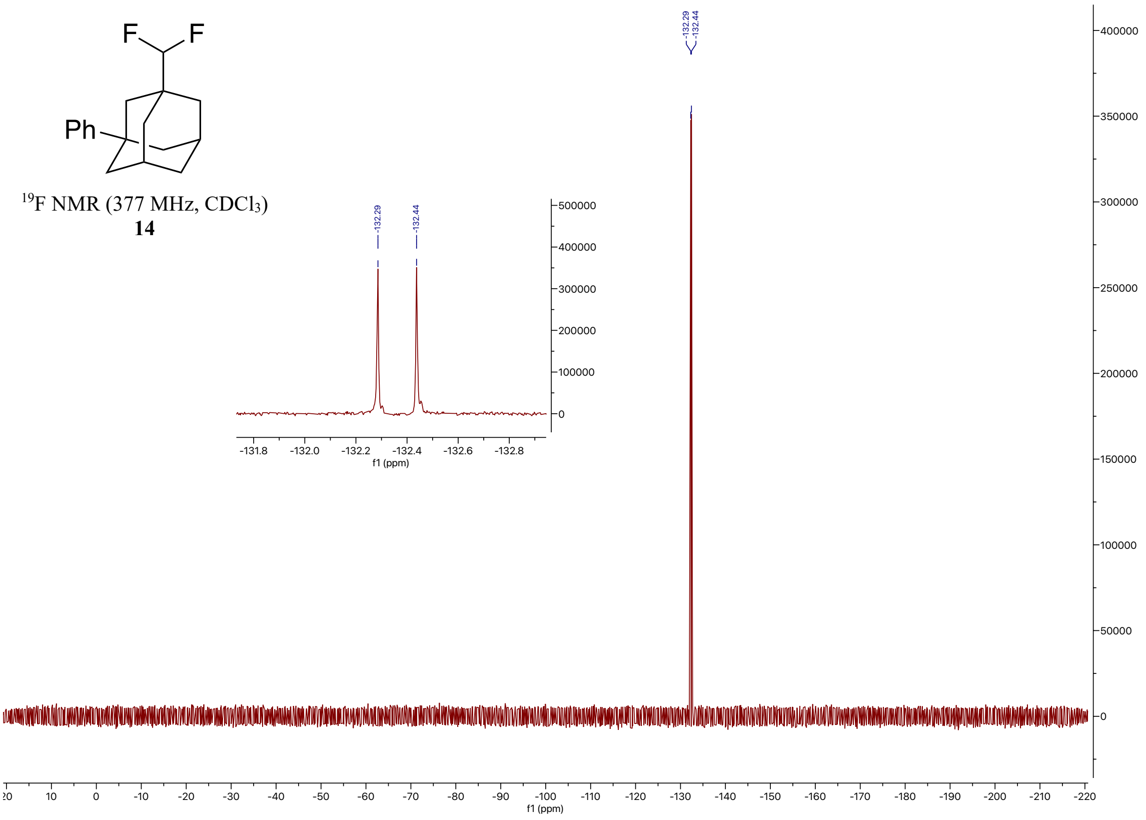

^{19}F NMR (377 MHz, $CDCl_3$)
14

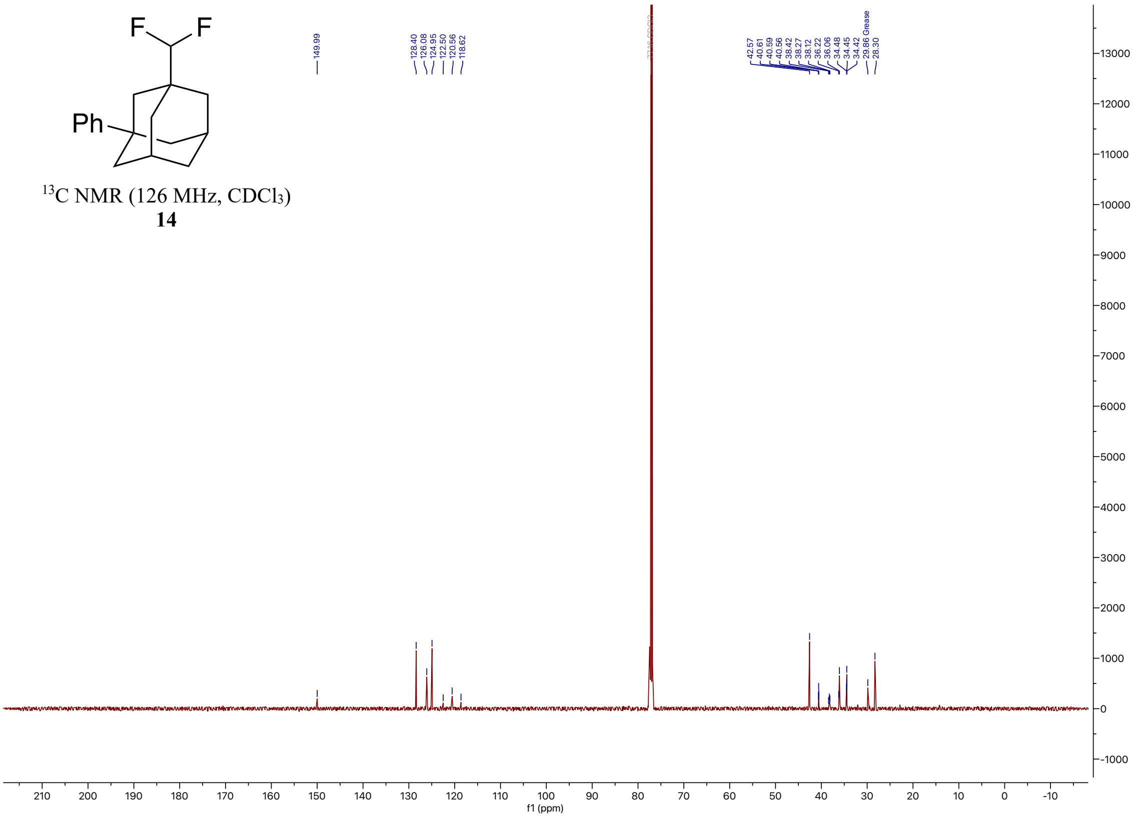

^{13}C NMR (126 MHz, $CDCl_3$)
14

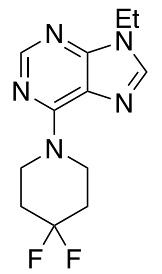

^{1}H NMR (400 MHz, $CDCl_3$)
19

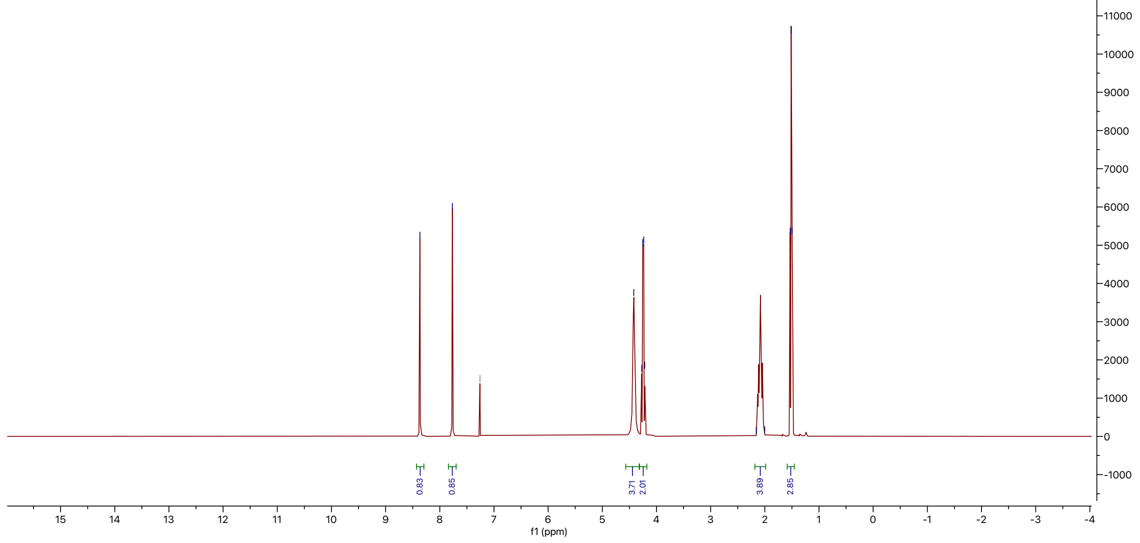

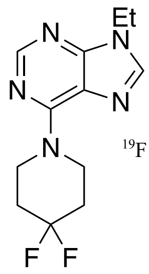

^{19}F NMR (377 MHz, $CDCl_3$)
19

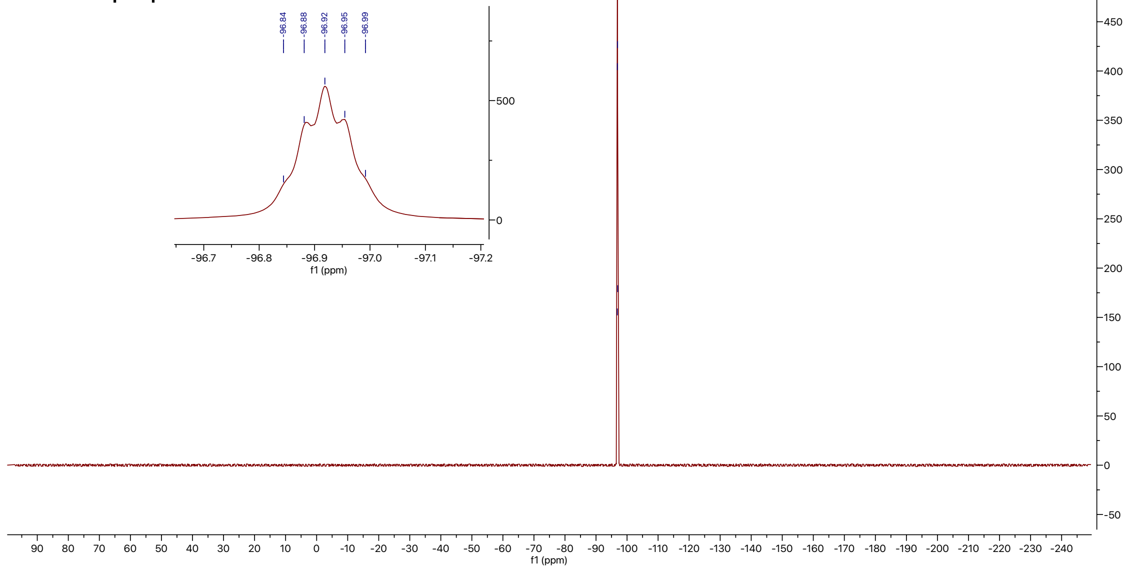

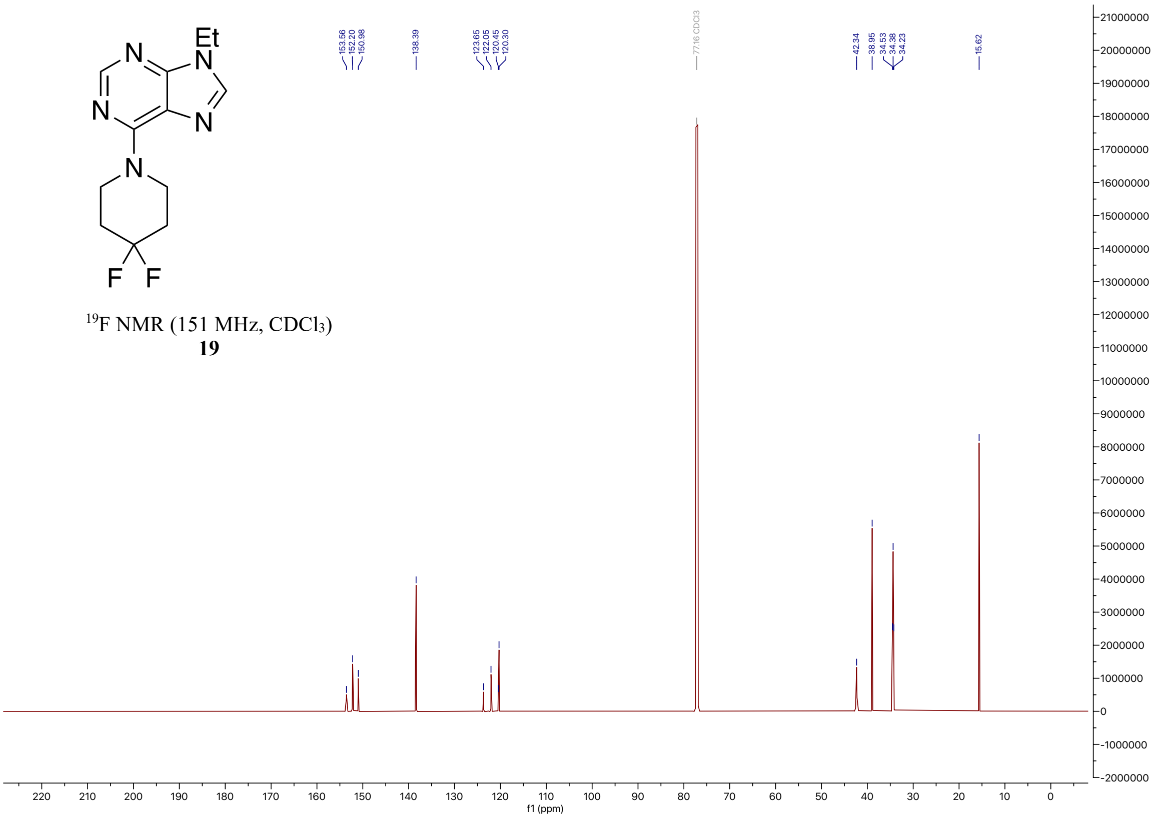

^{19}F NMR (151 MHz, $CDCl_3$)
19

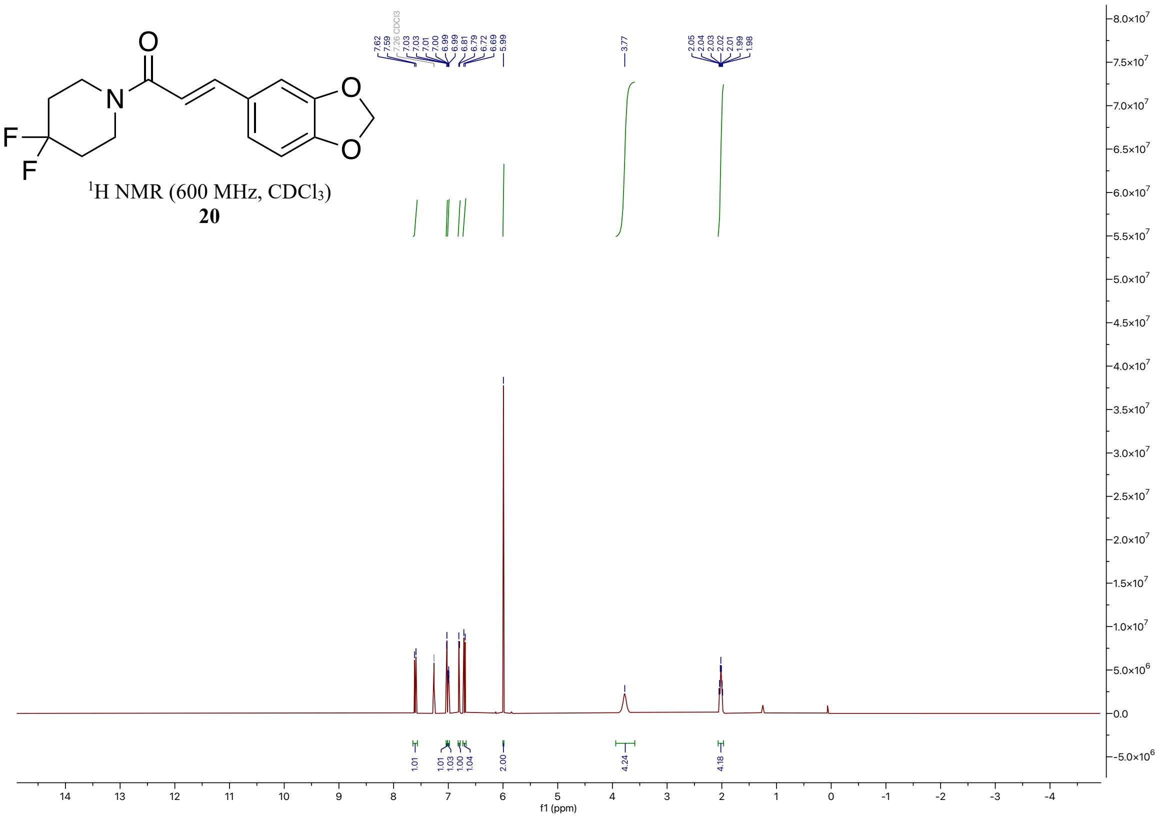

^{1}H NMR (600 MHz, $CDCl_3$)
20

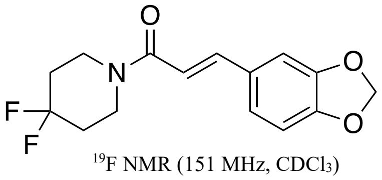

^{19}F NMR (151 MHz, $CDCl_3$)
20

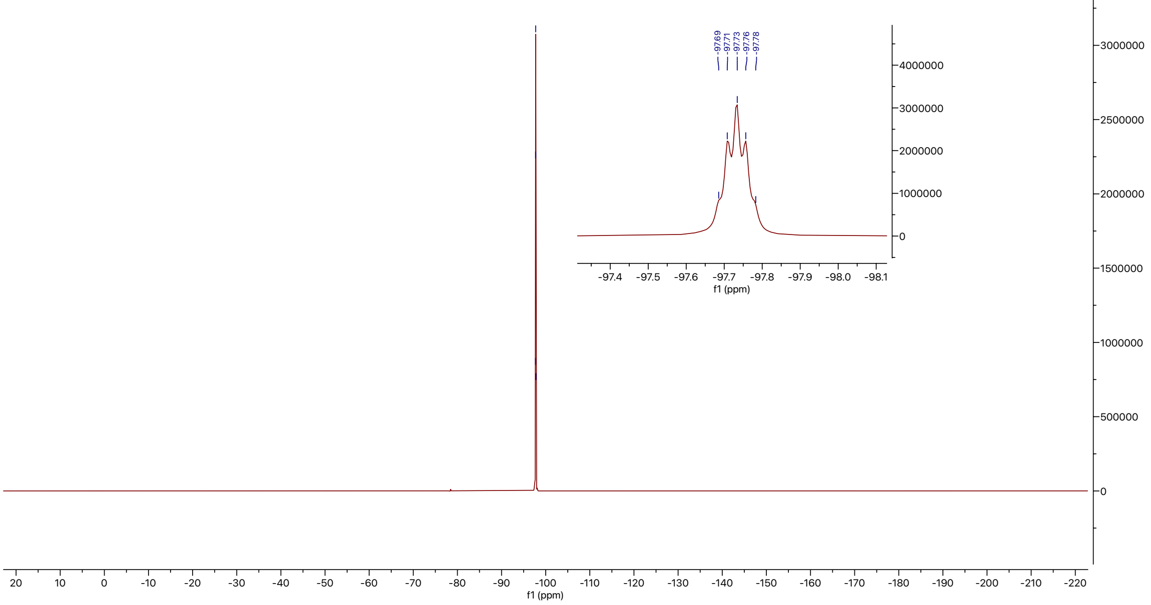

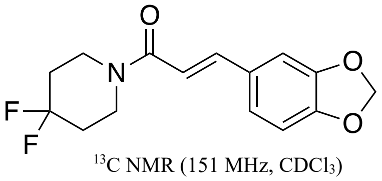

^{13}C NMR (151 MHz, $CDCl_3$)
20

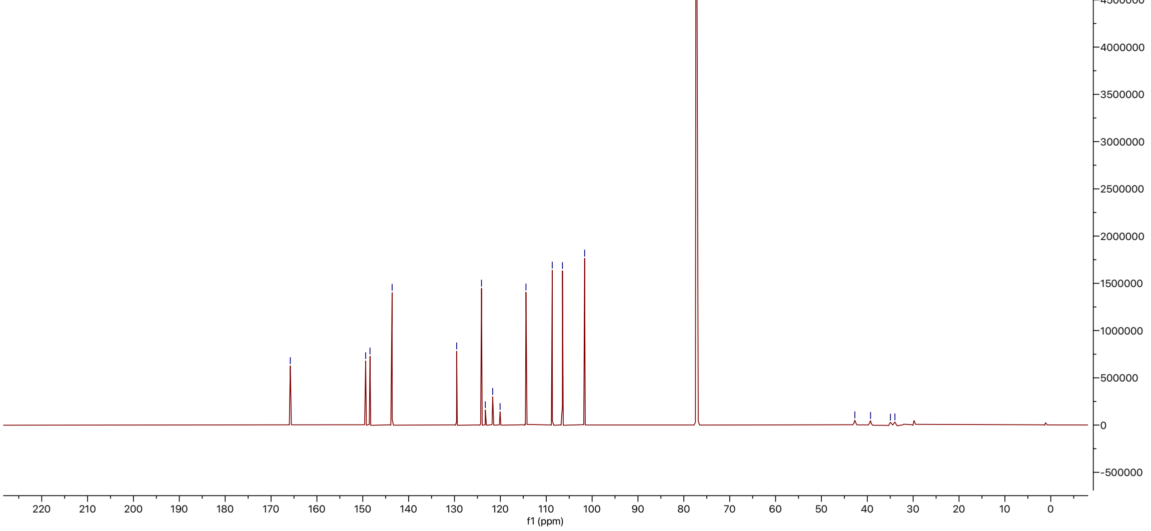

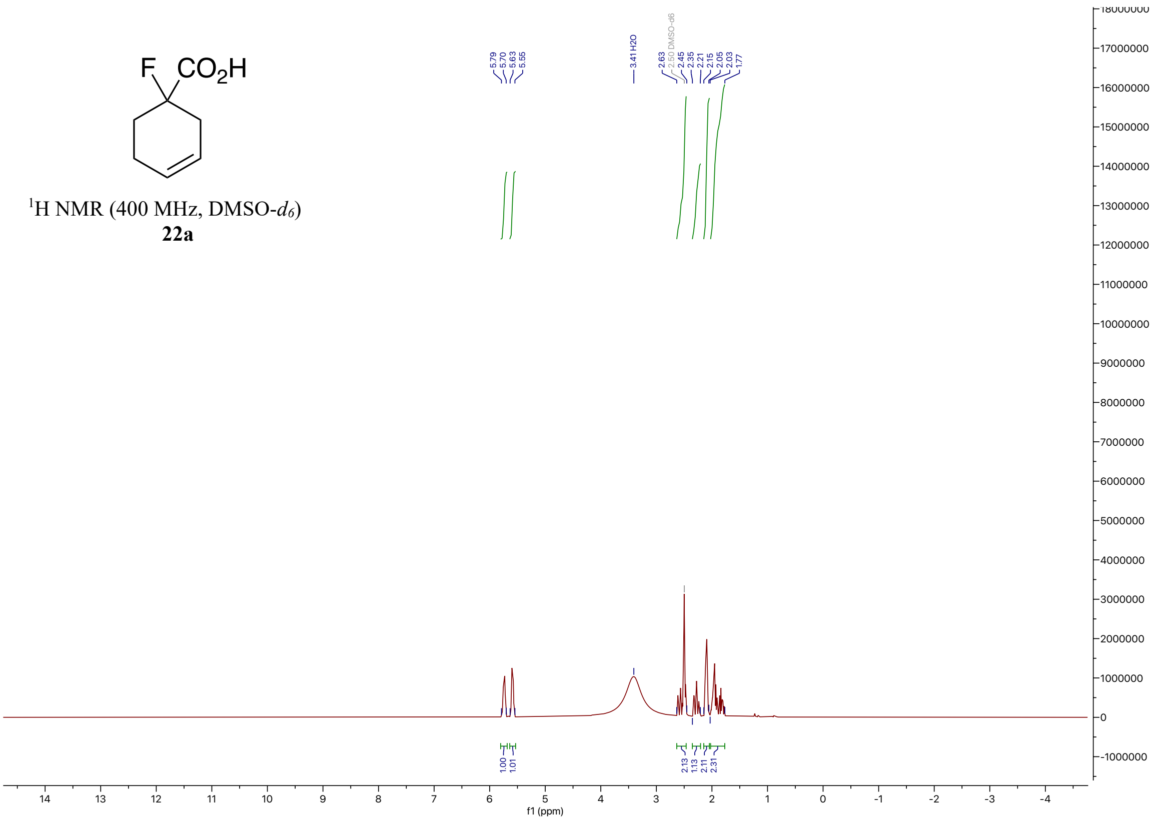

^{1}H NMR (400 MHz, DMSO-d_6)
22a

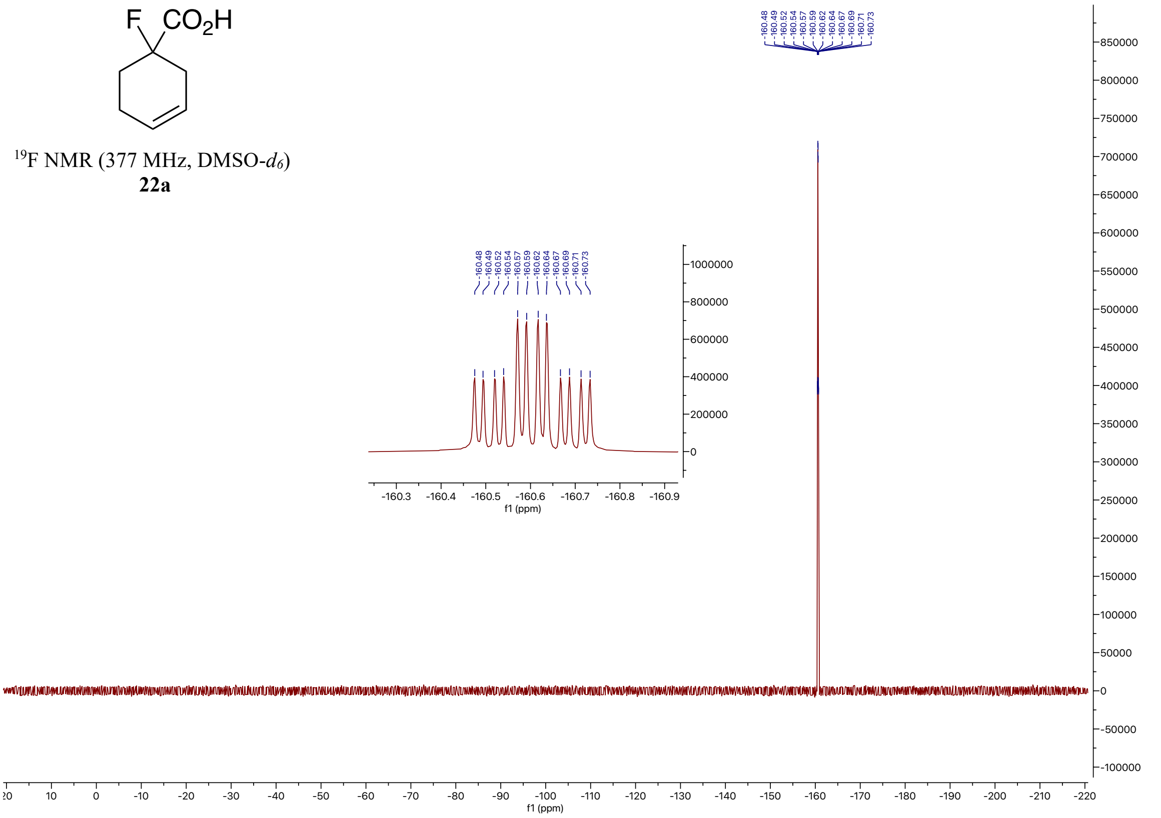

^{19}F NMR (377 MHz, DMSO-d_6)
22a

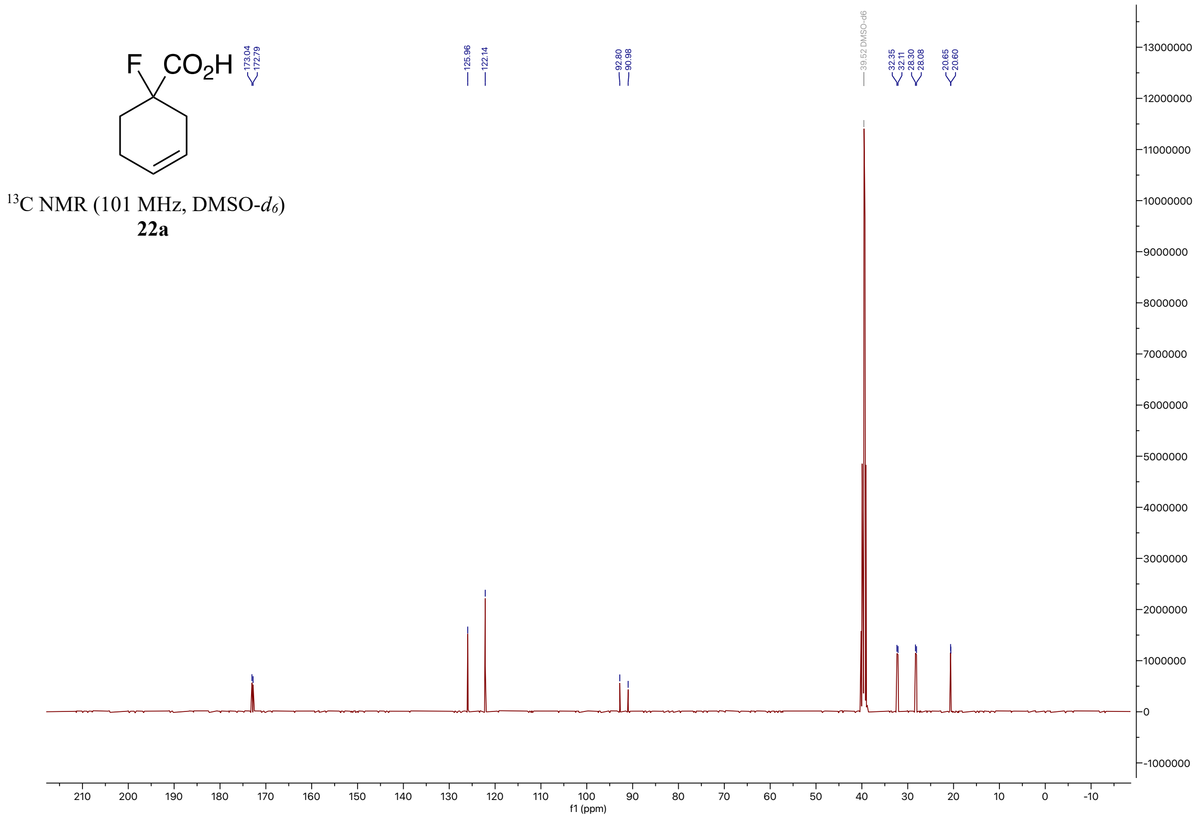

F
CO2H
173.04
172.79
125.96
122.14
92.80
90.98
39.52 DMSO-d6
32.35
32.11
28.30
28.08
20.65
20.60
f1 (ppm)

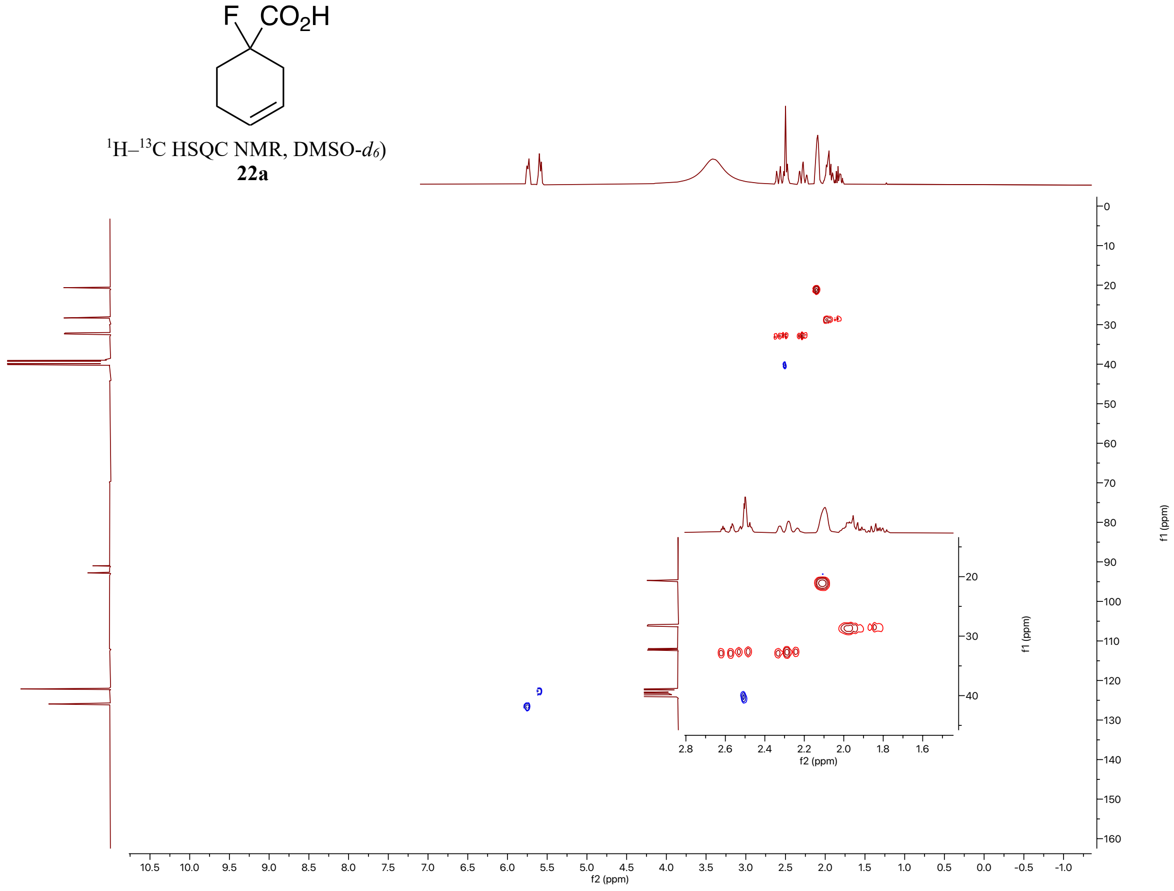

F
CO2H
f2 (ppm)
f1 (ppm)
